# Prediction of the Mechanisms by Which Quercetin Enhances Cisplatin Action in Cervical Cancer: A Network Pharmacology Study and Experimental Validation

**DOI:** 10.3389/fonc.2021.780387

**Published:** 2022-01-06

**Authors:** Huihui Ji, Kehan Li, Wenbin Xu, Ruyi Li, Shangdan Xie, Xueqiong Zhu

**Affiliations:** Center of Uterine Cancer Diagnosis and Therapy Research of Zhejiang Province, Department of Obstetrics and Gynecology, The Second Affiliated Hospital of Wenzhou Medical University, Wenzhou, China

**Keywords:** quercetin, cisplatin, cervical cancer, network pharmacology, treatment

## Abstract

Yimucao has been used as an herbal medicine to treat gynecological diseases. Common genes of Yimucao active compounds were investigated using network pharmacology. The components and targets of Yimucao were retrieved from the TCMSP database. Cervical cancer targets were collected from GeneCards, TTD, DisGeNET, and KEGG. Cisplatin-related genes were downloaded from GeneWeaver. The protein-protein interaction (PPI) network was created using the STRING database. A drug-bioactive compound-disease-target network was constructed using Cytoscape. GO and KEGG analyses were performed to investigate common targets of quercetin and cisplatin in cervical cancer. We found that quercetin was the highly bioactive compound in Yimucao. The drug-bioactive compound-disease-target network contained 93 nodes and 261 edges. Drug-related key targets were identified, including *EGFR*, *IL6*, *CASP3*, *VEGFA*, *MYC*, *CCND1*, *ERBB2*, *FOS*, *PPARG*, and *CASP8*. Core targets were primarily related to the response to metal ions, cellular response to xenobiotic stimulus, and transcription factor complex. The KEGG pathway analysis revealed that quercetin and cisplatin may affect cervical cancer through platinum drug resistance and the p53 and HIF-1 pathways. Furthermore, quercetin combined with cisplatin downregulated the expression of EGFR, MYC, CCND1, and ERBB2 proteins and upregulated CASP8 expression in HeLa and SiHa cells. Functionally, quercetin enhanced cisplatin-induced anticancer activity in cervical cancer cells. Our results indicate that quercetin can be used to overcome cisplatin resistance in cervical cancer cells.

## Introduction

More than 500,000 women are diagnosed with cervical cancer each year, which causes more than 300,000 deaths worldwide (1). The standardized incidence rate of cervical cancer is expected to be 13.1 cases per 100,000 women worldwide (2). Advanced or recurrent cervical cancer conveys a poor prognosis with a 10–20% 1-year relative survival rate (3). Current treatment approaches for locally advanced cervical cancer include brachytherapy, external beam radiotherapy, and concurrent chemotherapy based on cisplatin (4). Among them, cisplatin is one of the most widely used chemotherapeutic agents. However, 40% of locally advanced cervical cancer patients still experience disease recurrence (5). An economic cost estimate based on cervical cancer demonstrated that per capita expenditures of cervical cancer treatment ranged from US$368 to 11,400 (6). Cervical cancer places heavy burdens on both families and society. Traditional Chinese medicine (TCM) has been applied to treat cancer, having the advantage of acting on a variety of cancer-related signaling pathways and molecular targets without severe side effects (7).

Yimucao has been used for the uterotonic action, dysmenorrhea, lochia, and other gynecological disorders in China for thousands of years (8). Quercetin is a key compound of Yimucao that exhibits good antitumor activity (9, 10), especially for cervical cancer (11–13). One study used HPLC analysis and reported that Yimucao contents primarily include leonurine hydrochloride, quercetin, rutin, kaempferol, and apigenin (14). Many studies have investigated the effect of quercetin on cervical cancer cell apoptosis. For example, Bishayee and colleagues found that quercetin promotes cervical cancer cell apoptosis and inhibits cell cycle by inducing cytochrome c release and ROS accumulation in G2/M (15). One group confirmed that quercetin nanoparticles play a role in inhibiting cervical cancer progression by inhibiting JAK2, which induces autophagy, apoptosis, and antiproliferative activities (16). Furthermore, previous experiments have shown that quercetin promotes G2 phase arrest and apoptosis by activating the p53 signaling pathway (17). Quercetin might also reduce the MMP2, Ezrin, METTL3, and P-Gp expression in cancer cells, enhancing the antitumor effect of cisplatin by inhibiting the proliferation, migration, and invasion and potentiating apoptosis of cervical cancer cells (18). Most connections between Yimucao and the other therapeutic were obtained from previous experience and long-term clinical observations (8). However, the mechanism for the synergy between Yimucao and cisplatin for the treatment of cervical cancer is largely unexplored. Thus, it is necessary to extend the knowledge regarding the synergistic effects of Yimucao chemical compounds with cisplatin in treating cervical cancer using technological approaches.

Recently, the prediction of effective components and potential targets of Chinese medicine using network pharmacology has become increasingly popular for TCM modernization and internationalization (19–21). Network pharmacology enables a more complete understanding of the mechanism of medicine and disease using multiple authoritative databases (22). Chinese medicine treat many complicated diseases, including cancer, as a disturbance of interlinked complex biological networks and determines the drug mechanism of action in accordance with the network topology (23). For instance, Zhu and colleagues demonstrated that β-elemene inhibits peritoneal effusion production in pancreatic cancer by inhibiting the HIF1α-VEGFA pathway using the network pharmacology (24). They studied the molecular biological mechanism of Kushen compound injection and its anticancer effect using network pharmacology and found that the active ingredients of Kushen induced cancer cell apoptosis through the p53 and PI3K-Akt signaling pathways (25). Network pharmacology was also implemented to predict the mechanism of Fuzhengkangai in *EGFR* mutation-positive lung adenocarcinomas, and two gene targets, *BCL2* and *PRKCA*, were identified (26). These studies have demonstrated that network pharmacology is a useful predictive tool for exploring the chemical components of Yimucao and its effects on cervical cancer.

**Figure 1:**
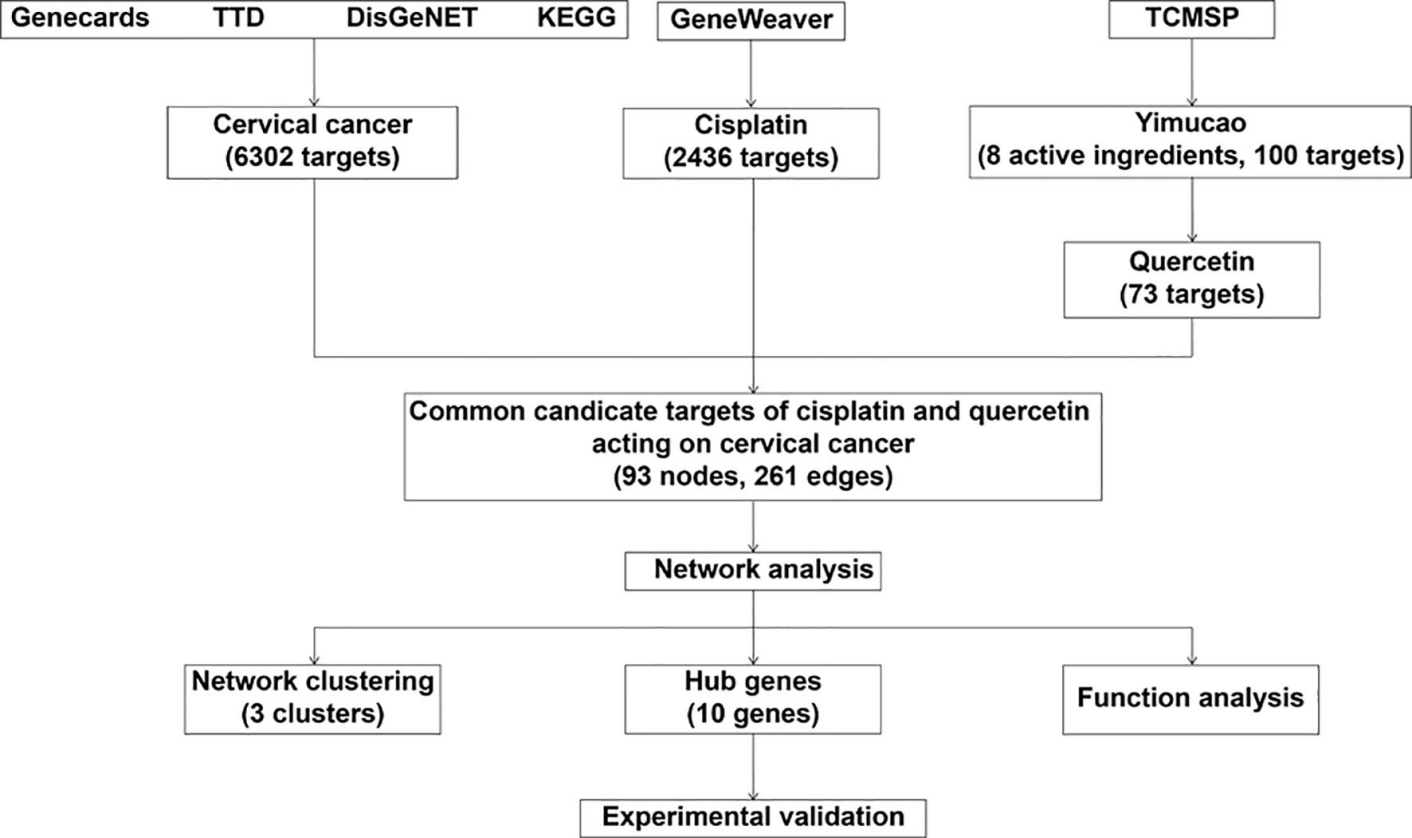
Schematic of the network pharmacological analysis used to identify the potential mechanism by which quercetin enhances cisplatin action in cervical cancer. TTD, Therapeutic Target Database; KEGG, Kyoto Encyclopedia of Genes and Genomes; TCMSP, Traditional Chinese Medicine Systems Pharmacology Database (TCMSP) database.

In the present study, a network pharmacology method was applied to identify the potential biologically active ingredients in Yimucao and to clarify the mechanism by which the active ingredients of Yimucao, particularly quercetin, enhance the effect of cisplatin on cervical cancer and related key proteins (**Figure 1**).

## Materials and Methods

### Screening and Identification of Yimucao’s Active Ingredients

Information about compounds from Yimucao was identified by searching the Traditional Chinese Medicine Systems Pharmacology Database and Analysis Platform (TCMSP, http://tcmspw.com/). TCMSP identifies compound–target networks (27). The herbs were input as the keywords to determine all components in TCMSP. Absorption, distribution, metabolism, and excretion (ADME) are strategic processes in drug discovery and development that were used to select the compounds of Yimucao (28). The effect of the compound on the disease is largely determined by the degree of oral bioavailability (OB) (29), and high OB is usually an essential indicator for determining the drug-likeness (DL) index of active substances. Substances with OB ≥ 30% were regarded to have high OB. The first-rank compounds and improved candidate compounds were selected using DL in the initial period of drug development (30). The average DL index in the DrugBank database was 0.18. Substances with a DL index ≥0.18 were regarded to have high druggability. Therefore, compounds with OB ≥ 30% and DL ≥ 0.18 were selected for further analysis in the present study (31).

### Prediction of Drug Targets for Yimucao and Cisplatin

The potential targets of active components in Yimucao were also obtained from the TCMSP database (27). The species of target proteins was limited to *Homo sapiens*, and their corresponding official symbols were obtained from UniProtKB (http://www.uniprot.org) (32). In addition, cisplatin-related genes were identified using the GeneWeaver database (33), which comprises a number of gene sets from multiple functional genomic data resources, curated publications, and user submissions. A large dataset consisting of 2,436 (GS125959) genes from humans was selected for further analysis.

### Predicting the Target Genes of Cervical Cancer

Cervical cancer-related genes were identified by searching the following databases: GeneCards (https://www.genecards.org/) (34), Therapeutic Target Database (TTD, https://db.idrblab.org/ttd/) (35), DisGeNET (https://www.disgenet.org/) (36), and Kyoto Encyclopedia of Genes and Genomes (KEGG, https://www.genome.jp/kegg/) (37), using the keyword “cervical cancer”. In brief, online databases were searched using the disease names to obtain corresponding target genes, and then the targets of cervical cancer were determined by removing duplicate targets.

### Construction of a Component–Target Gene Network

Cytoscape 3.8.0 was used to construct a component–target network to reflect the complex relationships between hypothetical bioactive ingredients and their targets (38). Cytoscape 3.8.0 is an open-source software platform that is applicable to exhibit complex networks and integrate different types of attribute data. The nodes of the network represent the screened active components and potential targets, while the connection between these nodes represents the biological interactions (39).

### Protein–Protein Interaction Construction

Common targets of Yimucao, cisplatin, and cervical cancer were collected as the core targets of Yimucao and cisplatin for cervical cancer. Core genes were identified by constructing a PPI. There are various databases related to PPIs, and the STRING database (https://string-db.org/) covers the greatest number of species and contains more interactive information (40). In this study, the core targets were submitted to STRING, with the species limited to *Homo sapiens* and confidence score limited to those > 0.4, to obtain PPI data. The value of “degree”, the number of correlations of the node in the whole network, was used as a reference to the importance of the core target.

“Molecular Complex Detection” (MCODE: a plug-in of Cytoscape) was applied to determine the relationships between network clusters to identify hub genes with a high degree of connectivity (41). According to previous literature, advanced options were set with a degree cutoff = 2, a node score cutoff = 0.2, and a K-Core = 2 (42–44).

### Gene Ontology and KEGG Pathway Enrichment Analysis

GO and KEGG pathway enrichment analyses were performed using the R package “ClusterProfifiler” to explore the gene functions (45, 46). The Benjamini Hochberg method was utilized to adjust the *P-*value. Only functional annotations with an enrichment *P-*value < 0.05 were chosen for further analyses.

### Cell Culture

Human cervical cancer cells (HeLa and SiHa) were purchased from the European Collection of Authenticated Cell Cultures. Dulbecco’s modified Eagle’s medium (DMEM, Gibco, USA) supplemented with 10% fetal bovine serum (Gibco, USA) was used to grow cells, at 37°C with 5% CO2 and 95% air.

In our preliminary experiment, the IC30 of quercetin in HeLa cells was 15 μM, and the IC30 of cisplatin was 10 μM, and the IC30 of quercetin and cisplatin in SiHa cells was 25 and 12 μM, respectively. Quercetin (≥95%, HPLC) was obtained from Sigma-Aldrich Company (CAS: 117-39-5, USA). Quercetin was prepared into a 200 nM stock using dimethyl sulfoxide and maintained at −20°C. Then, quercetin was prepared in complete culture medium at 15 and 25 μM to treat cervical cancer cells. Cisplatin (Sigma, USA) was prepared in phosphate-buffered saline (PBS) at a concentration of 10 mM, stored at −20°C, and then used with complete culture medium to prepare 10 and 12 μM to act on cervical cancer cells. Quercetin combined with cisplatin was used to treat cervical cancer Hela and SiHa cells: 15 μM quercetin combined with 10 μM cisplatin treated Hela cells for 24 h, and 25 μM quercetin combined with 12 μM cisplatin affected on SiHa cells for 48 h.

### Western Blot Analysis

HeLa and SiHa cells were seeded into a six-well plate at a density of 2.5×10^5^ cells/well and divided into four groups (blank control, quercetin, cisplatin, quercetin and cisplatin). Radioimmunoprecipitation assay (RIPA) lysis buffer (60 mM Tris-HCl, pH 6.8, 5% glycerol, 2% SDS) and bicinchoninic acid (BCA) protein determination kits (Thermo, USA) were applied to extract and quantify the total protein in the cells after incubation (24 h for HeLa; 48 h for SiHa cells). Proteins were electrophoresed in polyacrylamide gels and transferred to polyvinylidene fluoride (PVDF) membranes (Millipore, USA). Membranes were incubated with primary antibodies at 4°C overnight. Rabbit anti-human EGFR, rabbit anti-human IL-6, rabbit anti-human caspase-3, rabbit anti-human VEGFA, rabbit anti-human c-Myc, rabbit anti-human Cyclin D1, rabbit anti-human HER2/ErbB2, rabbit anti-human c-Fos, rabbit anti-human PPARγ, and mouse anti-human caspase-8 antibodies were purchased from Cell Signaling (Danvers, MA, USA) and used at a 1:1,000 dilution. Rabbit anti-human vinculin antibody (1:3,000; Proteintech, USA) was used as a control. After washing three times with Tris-buffered saline Tween (TBST), PVDF membranes and horseradish peroxidase-conjugated secondary antibody were incubated for 1.5 h at ambient temperature. Vinculin was employed as a loading control. Peroxidase-conjugated goat antirabbit antibody (1:5,000; Biosharp, China) and anti-mouse antibody (1:5,000; Biosharp, China) were used as secondary antibodies. Finally, the bound antibodies were identified using Immobilon Western chemiluminescent HRP substrate (Millipore, USA). Immunoblots were imaged using a Bio-Rad ChemiDoc TM XRS system (Bio-Rad, USA). Image J software version 1.8.0 (National Institutes of Health, USA) was applied to quantify the intensity of the target western blot.

### Cell Viability Assay

HeLa and SiHa cells were seeded in 96-well plates overnight. HeLa cells were treated with 15 μM quercetin and 15 μM cisplatin for 24 h, and SiHa cells were treated with 25 μM quercetin and 15 μM cisplatin for 48 h. Then, Cell Counting Kit-8 (CCK-8) was used to measure cell viability by a Microplate Reader as described previously (18).

### Flow Cytometry Analysis for Apoptosis

HeLa and SiHa cells were seeded in 6-well plates overnight. HeLa cells were treated with 15 μM quercetin and 10 μM cisplatin for 48 h, and SiHa cells were treated with 30 μM quercetin and 12 μM cisplatin for 48 h. Cell apoptosis was measured by Annexin V-FITC/propidium iodide (PI) method as described previously (18).

### Statistical Analysis

Data were entered and analyzed using SPSS 22.0 software (Chicago, USA). The results are expressed as the mean ± standard deviation (SD). Multiple groups were calculated using one-way analysis of variance (ANOVA) followed by Tukey’s posttest. A two-tailed *p-*value < 0.05 was regarded as statistically significant.

## Results

### The Construction of an Herb–Compound–Target Network

**Figure 2:**
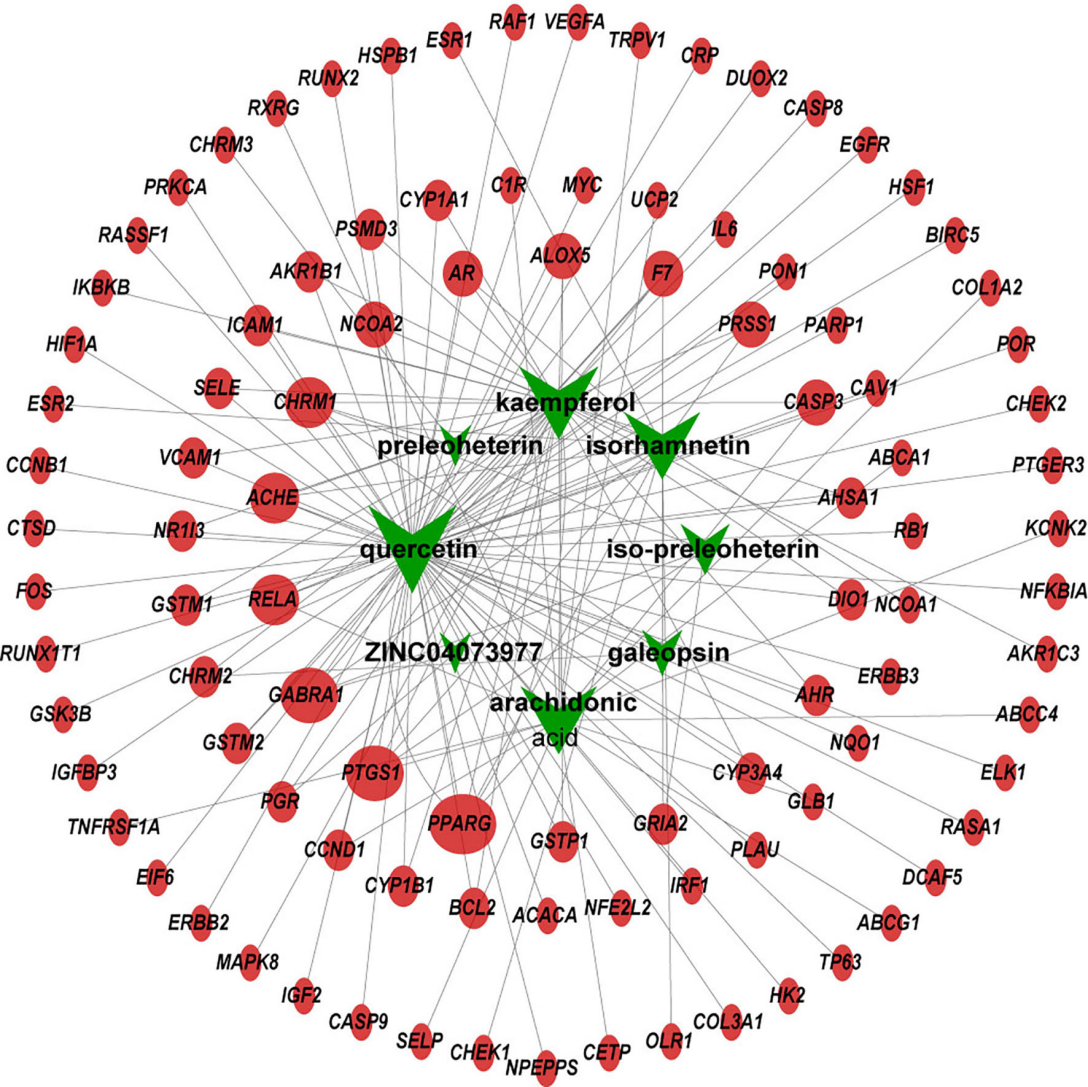
Compound-target network of Yimucao. Green nodes are presented to compounds in Yimucao. Red circle nodes represent for the target genes. The size of the node indicates the size of the degree.

**Figure 3:**
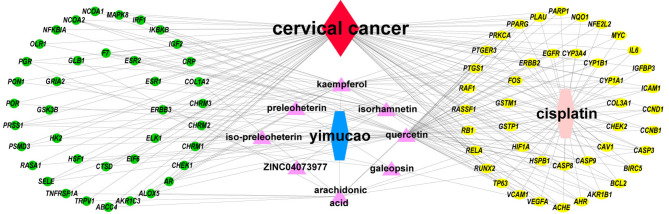
Drug-bioactive compound-disease-target network of combined treatment with Yimucao and cisplatin for cervical cancer. The green circle represents the gene, the yellow circle represents overlapping genes of cisplatin, quercetin, and cervical cancer target genes, and the pink triangle represents compounds of Yimucao. The red diamond indicates cervical cancer. The blue and pink hexagons represent Yimucao and cisplatin, respectively.

**Figure 4:**
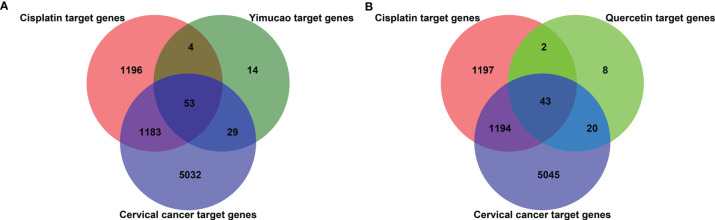
Quercetin and cisplatin target gene prediction for cervical cancer treatment. The overlapping number of cisplatin, Yimucao **(A)** or quercetin **(B)**, and cervical cancer target genes. The red circle represents cisplatin target genes, the purple circle represents cervical cancer target genes, and the dark green and bright circles represent Yimucao and quercetin target genes, respectively.

Using TCMSP, a total of 42 active compounds in Yimucao were identified based on the predefined criteria (OB ≥ 30% and DL ≥0.18) (**Supplementary Table 1**), and 8 active compounds, including quercetin, were identified from the herb. The details of the active compounds and their structures are described in **Table 1** and **Supplementary Figure 1**. Based on the target prediction system in the TCMSP database, 100 putative target genes were identified for Yimucao (**Supplementary Table 2**). In addition, a total of 2,436 cisplatin-related genes were collected from GeneWeaver. Cervical cancer target genes were retrieved from GeneCards, TTD, DisGeNET, and OMIM. In total, 6,302 potential target genes related to cervical cancer were collected. Eight active compounds and 100 Yimucao target proteins were used to construct a network consisting of 108 nodes and 154 edges (**Figure 2**). As showed in this compound-target network, nodes with a greater number of edges were more important within the network and are presented as larger in size were those on which to focus. The network with drug-bioactive compounds and disease targets contained 93 nodes (including 1 disease, 1 chemotherapy drug, 1 herb, 8 compounds, and 82 genes) and 261 edges (**Figure 3**). By intersecting 100 target genes of Yimucao, and 2,436 target genes of cisplatin with the 6,302 potential target genes related to cervical cancer, a total of 53 intersections were obtained (**Figure 4A**).

**Table 1:** Bioactive compounds of Yimucao.

Mol ID	Compound name	OB	DL
MOL000098	quercetin	46.43	0.28
MOL001418	galeopsin	61.02	0.38
MOL001420	ZINC04073977	38	0.76
MOL001421	preleoheterin	85.97	0.33
MOL001422	iso-preleoheterin	66.29	0.33
MOL001439	arachidonic acid	45.57	0.2
MOL000354	isorhamnetin	49.6	0.31
MOL000422	kaempferol	41.88	0.24

OB, oral bioavailability; DL, drug-likeness.

### Network Construction of the Synergy Between Quercetin and Cisplatin Anti-Cervical Cancer Genes

Quercetin was the top ingredient node with the greatest number of edges. The intersection of 73 quercetin targets, 2,436 cisplatin targets, and 6,302 potential target genes related to cervical cancer revealed 43 genes. These intersections were considered potential candidate targets of the synergistic effect of quercetin and cisplatin against cervical cancer (**Supplementary Table 3** and **Figure 4B**).

**Figure 5:**
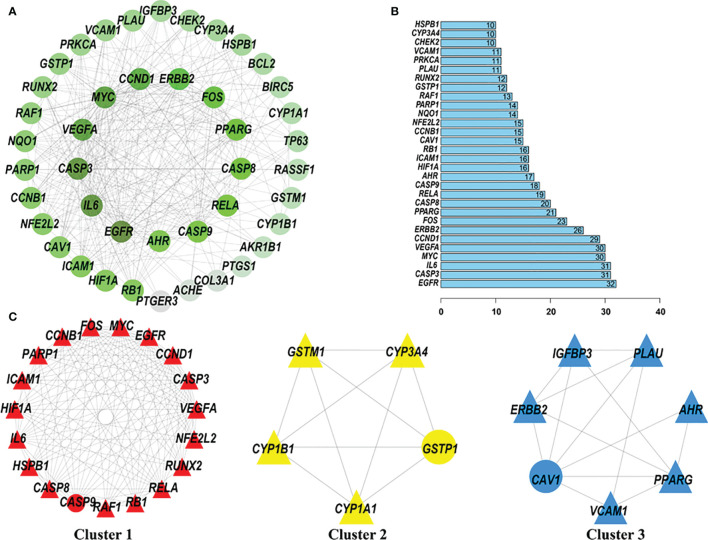
Protein-protein interaction (PPI) network of quercetin and cisplatin anti-cervical cancer genes. The MCODE algorithm was applied to this network to identify neighborhoods where proteins were densely connected. The gradient from dark green to light green represents the change in the number of edges from more to less; three significant modules with a score >4.5 were selected. **(A)** PPI network of quercetin and cisplatin anti-cervical cancer genes. **(B)** Histogram of key proteins. The y-axis represents the name of genes, the x-axis represents the number of adjacent genes, and height is the number of gene connections. **(C)** Module 1, MCODE score = 14.111; Module 2, MCODE score = 5; and Module 3, MCODE score = 4.667.

After extracting information regarding the relationships between potential anti-cervical cancer target genes of quercetin and cisplatin from the STRING website, a gene-gene interaction network with 43 nodes and 317 edges was constructed (**Figure 5A**). In the PPI network, the core genes with higher degree values were *EGFR*, *IL6*, *CASP3*, *VEGFA*, *MYC*, *CCND1*, *ERBB2*, *FOS*, *PPARG*, and *CASP8*. The number of edges connected to these nodes was relatively high (32, 31, 31, 30, 30, 29, 26, 23, 21, and 20, respectively) (**Figure 5B**).

Then, three subclusters within the PPI network were identified using the MCODE tool (**Figure 5C** and **Table 2**). The highest-scoring cluster, cluster 1, contained 19 nodes and 127 edges, including *IL6, PARP1, RUNX2, CCND1, RB1, EGFR, CASP9, HIF1A, VEGFA, NFE2L2, CCNB1, MYC, CASP3, CASP8, HSPB1, RAF1, ICAM1, FOS*, and *RELA*. Cluster 2 contained 5 nodes (*CYP1B1, GSTM1, CYP1A1, CYP3A4*, and *GSTP1*) and 10 edges. Cluster 3 contained 7 nodes and 14 edges.

**Table 2:** Cluster of target genes of quercetin and cisplatin anti-cervical cancer protein-protein interaction network.

Cluster	Score	Nodes	Edges	Node IDs
1	14.111	19	127	*IL6, PARP1, RUNX2, CCND1, RB1, EGFR, CASP9, HIF1A, VEGFA, NFE2L2, CCNB1, MYC, CASP3, CASP8, HSPB1, RAF1, ICAM1, FOS, RELA*
2	5	5	10	*CYP1B1, GSTM1, CYP1A1, CYP3A4, GSTP1*
3	4.667	7	14	*IGFBP3, PPARG, AHR, CAV1, PLAU, ERBB2, VCAM1*

### GO and KEGG Enrichment Analysis of Candidate Target Genes

**Figure 6:**
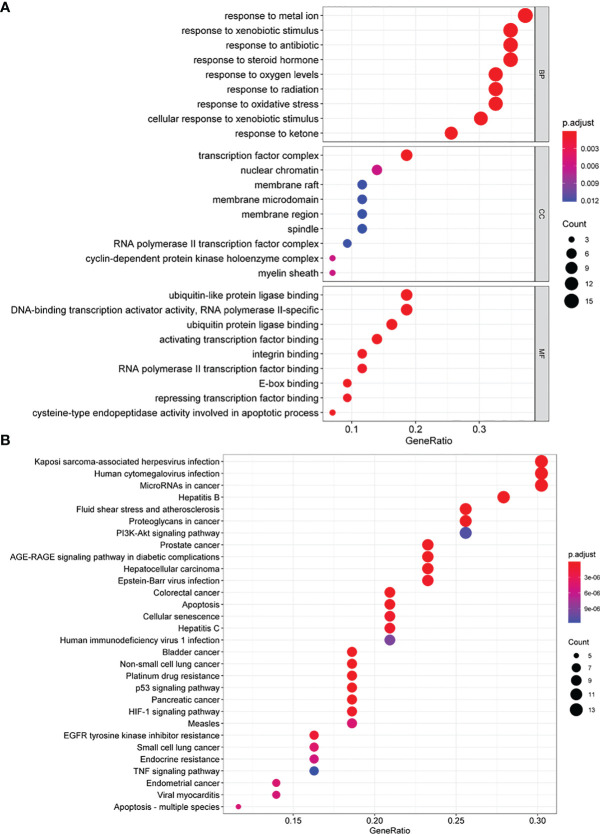
GO and KEGG enrichment analysis of quercetin synergism with cisplatin anti-cervical cancer genes. **(A)** The GO enrichment analysis of quercetin synergizing with cisplatin anti-cervical cancer genes. Ontology covers three domains: biological process (BP), cellular component (CC), and molecular function (MF). The abscissa GeneRatio represents the proportion of genes of interest in the entry, and the ordinate represents each entry. The size of the dots represents the number of genes annotated in the entry, and the color of the dots represents the corrected *P*-value of the hypergeometric test. **(B)** The KEGG enrichment analysis of quercetin synergizing with cisplatin to target anti-cervical cancer genes. The abscissa represents the number of genes annotated in the pathway, the ordinate represents the pathway, and the color of the column represents the corrected *P*-value. KEGG, Kyoto Encyclopedia of Genes and Genomes; GO, Gene Ontology.

To further understand the identified candidate target genes, GO enrichment analysis, including biological process (BP), cell composition (CC), and molecular function (MF), was conducted. Enriched GO terms are shown in **Figure 6A** and **Supplementary Table 4**. In the BP group, the active targets were essentially connected to “response to metal ion”, “cellular response to xenobiotic stimulus”, “response to radiation”, and “response to oxidative stress”. In the CC group, the genes were primarily enriched in “transcription factor complex”, “cyclin-dependent protein kinase holoenzyme complex”, “nuclear chromatin”, and “RNA polymerase II transcription factor complex”. In the MF group, the majority of genes were enriched in “activating transcription factor binding”, “E-box binding”, “DNA-binding transcription activator activity, RNA polymerase II-specific”, and “repressing transcription factor binding”.

KEGG pathway enrichment was used to explore the major effects that associated with the quercetin. A total of 102 signaling pathways (**Figure 6B** and **Supplementary Table 5**) were selected (*P* < 0.05). The principal pathways were related to cancer, including “platinum drug resistance”, “p53 signaling pathway”, “HIF-1 signaling pathway”, “proteoglycans in cancer”, “apoptosis”, “PI3K-Akt signaling pathway”, and “TNF signaling pathway”.

### Using Western Blotting to Analyze the Related Proteins by Which Quercetin Promotes the Effect of Cisplatin on Cervical Cancer Cells

**Figure 7:**
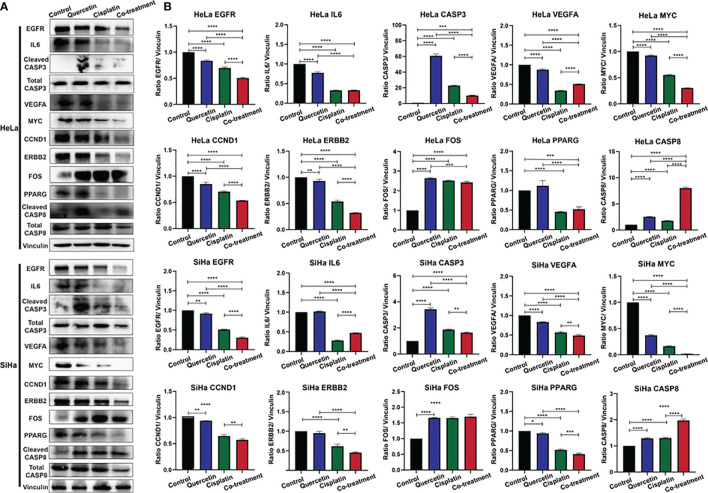
Western blotting was used to detect the relationship between the target protein and cervical cancer. **(A)** Immunoblot of EGFR, IL6, CASP3, VEGFA, MYC, CCND1, ERBB2, FOS, PPARG, and CASP8 in HeLa cells and SiHa cells. Vinculin was used as a loading control. **(B)** Densitometry for quantitation of relative differences in band intensity for of EGFR, IL6, CASP3, VEGFA, MYC, CCND1, ERBB2, FOS, PPARG, and CASP8 normalized to vinculin in **(A)**. *P < 0.05, **P < 0.01, ***P < 0.001, ****P < 0.0001.

Changes in 10 corresponding key protein levels in cervical cancer cells (HeLa or SiHa cells) in response to different drug combinations were detected by western blot analysis (**Figure 7**). After treatment with quercetin or cisplatin, the protein expression of EGFR, VEGFA, MYC, and CCND1 in HeLa and SiHa cell lines was significantly decreased, and the expression levels of CASP3, FOS, and CASP8 were significantly increased. In both HeLa and SiHa cell lines, IL6, ERBB2, and PPARG protein levels were significantly decreased after treatment with cisplatin. However, significant downregulation of IL6 and ERBB2 in response to treatment with cisplatin was only observed in SiHa cells, and significant downregulation of PPARG was only found in HeLa cells.

To explore the synergistic effect of quercetin on cisplatin in cervical cancer, a combination treatment with quercetin and cisplatin was next explored. Protein levels of EGFR, MYC, CCND1, and ERBB2 in HeLa and SiHa cells were significantly decreased compared to those of HeLa and SiHa cells treated with both drugs. In the combination group, a significant increase in CASP8 protein levels was observed compared to either a single drug in HeLa and SiHa cells. Compared to the control group or quercetin treatment group, significant reductions in the IL6 and PPARG protein levels were observed in the combination group in HeLa and SiHa cells, but increased and decreased levels of IL6 and PPARG in the combination group were only observed in SiHa cells compared to cisplatin treatment alone. In HeLa and SiHa cells, significantly higher CASP3 protein expression was observed in the combination group than in the control group, and decreased levels of CASP3 were observed in the combination group compared to the single drug groups. Compared to the control or quercetin treatment group, VEGFA protein levels were significantly decreased in the combination group in the two cell lines, but increased and decreased levels in the combination group compared to the quercetin treatment group were only found in HeLa cells and SiHa cells, respectively. Compared to the control group, FOS levels were significantly increased in the combination group in the two cell lines, but were significantly decreased in the combination group compared to the quercetin treatment group in HeLa cell.

### Quercetin Enhanced the Anticancer Effect of Cisplatin on Cell Viability and Apoptosis in Cervical Cancer Cells

**Figure 8:**
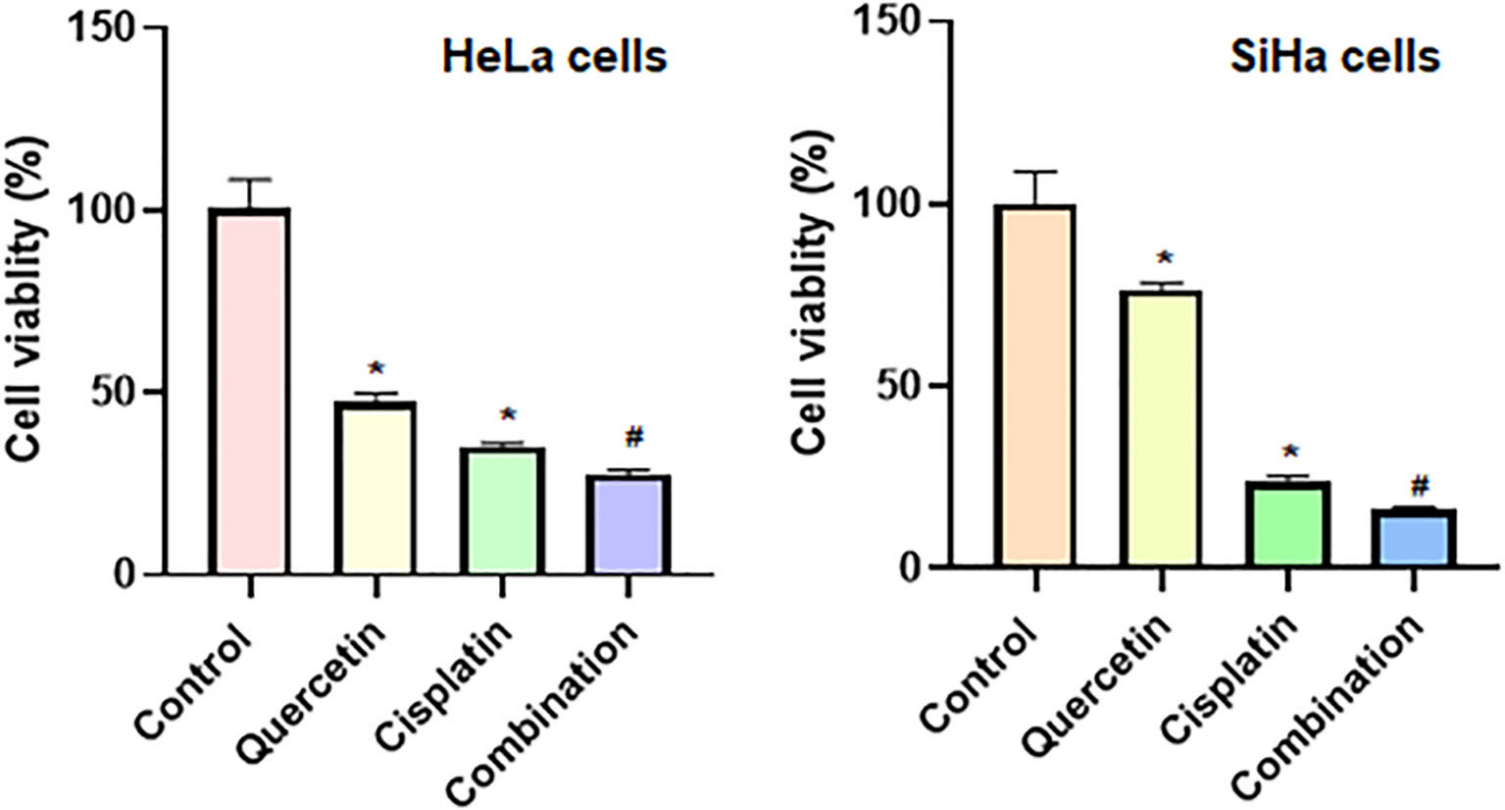
Quercetin enhances cisplatin-induced inhibition of viability of cervical cancer cells. CCK-8 assay was utilized to measure the cell viability in HeLa and SiHa cells after treatments with quercetin or cisplatin or combination. HeLa cells were treated with 15 μM quercetin and 15 μM cisplatin for 24 h, and SiHa cells were treated with 25 μM quercetin and 15 μM cisplatin for 48 h. *P < 0.05 *vs* control group; #*P* < 0.05 *vs* quercetin alone or cisplatin alone.

**Figure 9:**
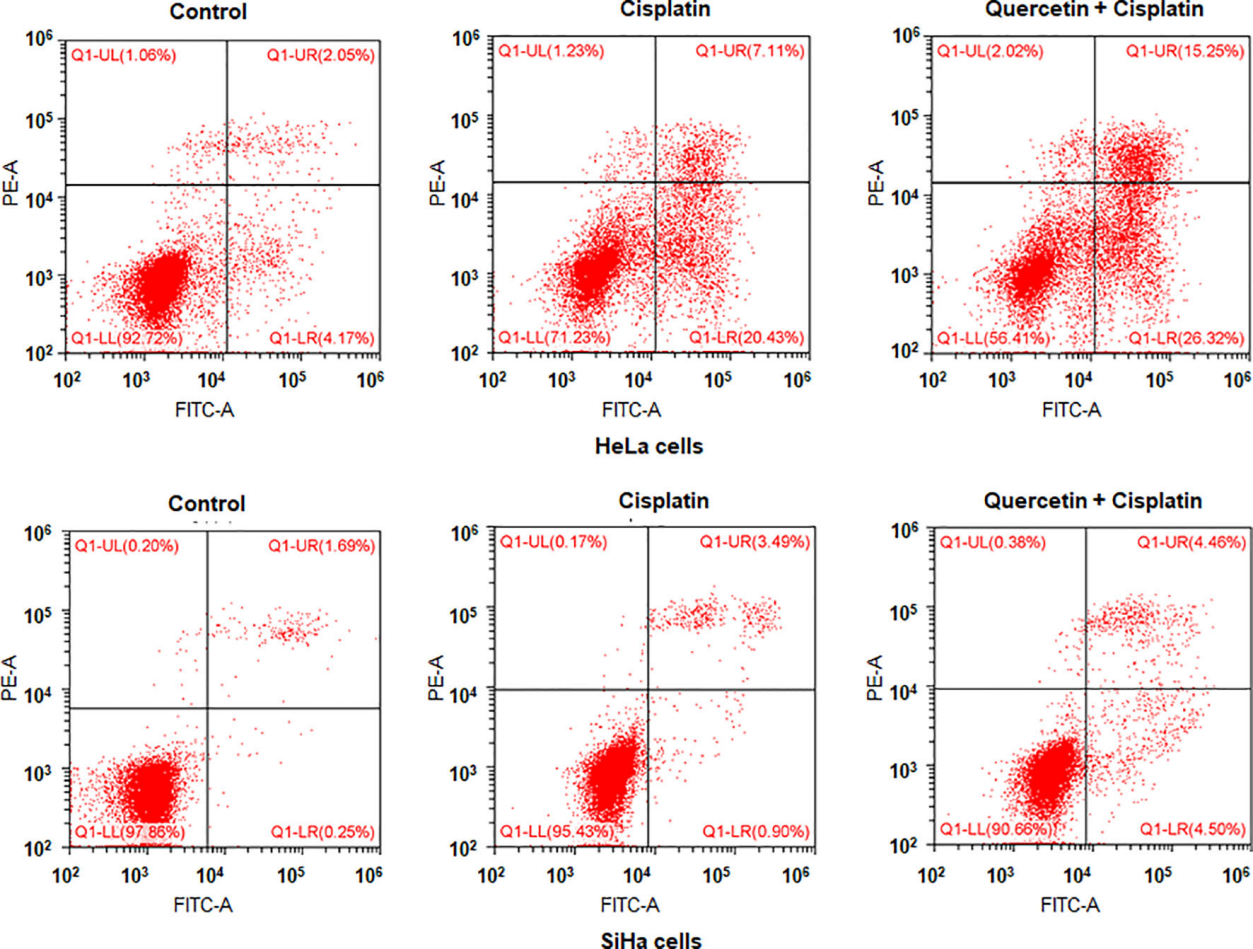
Quercetin enhances cisplatin-induced apoptosis of cervical cancer cells. HeLa cells were treated with 15 μM quercetin and 10 μM cisplatin for 48 h, and SiHa cells were treated with 30 μM quercetin and 12 μM cisplatin for 48 h. Flow cytometry analysis was utilized to measure apoptosis of HeLa and SiHa cells after various treatments.

To determine whether quercetin could promote the antitumor activity of cisplatin in cervical cancer cells, we treated HeLa and SiHa cells with quercetin or cisplatin or combination. The results from CCK-8 assay showed that quercetin inhibited cell viability in both HeLa and SiHa cells. Moreover, quercetin promoted the cisplatin-mediated suppression of cell viability in both HeLa and SiHa cells (**Figure 8**). Furthermore, we observed that cisplatin induced cell apoptosis in cervical cancer cells, which was further enhanced by quercetin treatment (**Figure 9**). Taken together, quercetin can enhance cisplatin-mediated antitumor activity in cervical cancer cells *via* regulating cell viability and apoptosis.

## Discussion

Our previous study had found that an active ingredient of Yimucao, leonurine, combined with cisplatin exerted synergistic antitumorigenic effects on cervical cancer (47); however, the detailed mechanism of Yimucao’s actions and its active ingredients’ synergism with cisplatin on cervical cancer is currently unknown. In the present study, we attempted to illustrate the synergistic mechanism of Yimucao and cisplatin by applying a network pharmacology approach. We identified 8 active compounds and 100 target proteins of Yimucao. Then, 73 potential targets of quercetin and cisplatin related to cervical cancer were obtained by constructing a biological network of interactions between chemical compounds and protein targets at the molecular systemic level. The predicted results indicated that quercetin and cisplatin interacted with 43 targets related to cervical cancer. The synergistic inhibitory effects of quercetin and cisplatin on cervical cancer cells were observed in our previous study (18). Quercetin and cisplatin modulate the same targets simultaneously, which explains their synergistic mechanisms. The PPI network indicated that *EGFR*, *IL6*, *CASP3*, *VEGFA*, *MYC*, *CCND1*, *ERBB2*, *FOS*, *PPARG*, and *CASP8* may play key roles in the effect of quercetin and cisplatin against cervical cancer. GO functional analysis revealed that genes were primarily related to the transcription initiation process, such as cyclin-dependent protein kinase holoenzyme complex, transcription factor complex, activating transcription factor binding, and E-box binding. The KEGG analyses demonstrated that 102 common targets were enriched in signaling pathways, including the platinum drug resistance, p53 signaling pathway, proteoglycans in cancer, apoptosis, PI3K-Akt signaling pathway, TNF signaling pathway, and HIF-1 signaling pathway. *In vitro* experiments verified that in response to cisplatin or quercetin treatment, expression of EGFR, IL6, VEGFA, MYC, CCND1, ERBB2, and PPARG was significantly reduced, while the expression of CASP3, FOS, and CASP8 was significantly increased. In addition, significant changes in the protein expression of five genes (EGFR, MYC, CCND1, ERBB2, and CASP8) were observed in the cells treated with the combination of both two drugs to cells treated with either drug alone. Our results demonstrated that these five genes were related to the synergistic effect of quercetin and cisplatin.

*EGFR* belongs to the family of HER tyrosine kinase receptor family and has a regulatory role in cell proliferation, transformation, and survival (48). It was estimated that 90% of cervical cancers overexpress *EGFR*, and high expression of *EGFR* is associated with poorer prognosis (49). Propofol has been reported to enhance the antitumor effect of cisplatin through the EGFR/JAK2/STAT3 pathway (50). Meanwhile, it has also been reported that TGF-α and amphiregulin inhibit the Hippo signaling pathway and activate YAP through EGFR to induce the proliferation and migration of cervical cancer cells (51). Two prospective studies revealed that cisplatin combined with anti-EFGR monoclonal antibody followed by surgery in locally advanced cervical cancer patients leads to excellent treatment outcomes (52, 53). *ERBB2* is another important therapeutic target of the epidermal growth factor receptor family in the treatment of malignant tumors (54). As a proto-oncogene, *ERBB2* is considered to be a therapeutic target for cervical cancer (55). Whole-exome sequencing of cervical carcinoma results confirmed that the ERBB2/PIK3CA/AKT/mTOR pathway was one of the major mechanisms of cervical cancer in tumorigenesis (56). Moreover, ERBB2 activation was reported as one of the key pathways in cisplatin-resistance in ovarian cancer (57, 58). *CCND1* belongs to the highly conserved cyclin family and plays a key role in the cell cycle transition from G1 phase to S phase transition (59). *MYC* is a proto-oncogene that encodes a nuclear phosphoprotein and is involved in cell proliferation, the cell cycle, and apoptosis (60). However, there have been no reports regarding the role of the four genes in quercetin treatment of cervical cancer.

Mutations in the upstream signaling molecules *EGFR* and *ERBB2* have been reported to be associated with increased NF-κB signaling. NF-κB, in turn, promotes transcription of the downstream proliferation-regulating genes *CCND1* and *MYC*, which participate in *VEGF*-dependent angiogenesis, metastasis, and cellular telomerase immortality (61). Studies have demonstrated that NF-κB is a key factor to confer drug resistance in human cancer (61). The cytoplasmic NF-κB expression is correlated with a trend toward poor outcomes in locally advanced cervical cancer patients after chemoradiation therapy (62). Our results demonstrated that quercetin combined with cisplatin has a synergistic effect on cervical cancer, leading to decreased protein expression of EGFR, MYC, CCND1, and ERBB2. Our network pharmacology research indicated that these four proliferation-related genes are therapeutic targets for quercetin to promote cisplatin-mediated inhibition of proliferation in cervical cancer cells.

Previous studies have shown that cisplatin acts on CASP8 in cervical cancer. Cisplatin in combination with death receptor ligands enhances the expression of CASP8 in the HPV 16-positive cervical cancer cell line SiHa to increase apoptosis (63). In addition, by enhancing exogenous and endogenous apoptotic pathways, cisplatin amplifies *CASP8* activation, sensitizing cervical cancer cells to apoptosis induced by rosulumab (64). However, the effect of quercetin on CASP8 in cervical cancer has not been reported. One study showed that loss of CASP8 activation is a potential mechanism for cisplatin resistance in human cancer cells (65). Our results demonstrated that in the treatment of cervical cancer, cisplatin combined with quercetin increases the expression of CASP8, indicating that CASP8 may be the key factor of quercetin in promoting the apoptosis of cervical cancer cells in combination with cisplatin for the treatment of cervical cancer. Indeed, our results demonstrated that quercetin enhanced cisplatin-mediated antitumor activity in cervical cancer cells *via* regulating cell viability and apoptosis.

This study extends our knowledge regarding the potential role of quercetin in cervical cancer and exhibits a feasible method for revealing potential drugs from Chinese medicinal formulas. However, there are still several limitations in the present work. First, more comprehensive TCM target gene databases are needed to deliver more credible consequences of network pharmacological analysis. Second, animal experiments are needed in order to verify the results of this study. Third, it is necessary to explore the changes in these five genes in response to single-drug therapy or two-drug combination therapy.

In conclusion, network pharmacology indicated that the primary active ingredients of Yimucao, particularly quercetin, could act on multiple targets in cervical cancer. Quercetin exerted a synergistic effect on cisplatin-treated cervical cancer cells primarily through downregulating the protein levels of EGFR, MYC, CCND1, and ERBB2 and upregulating CASP8. Quercetin promoted cisplatin-induced antitumor activity in cervical cancer cells *via* regulating cell viability and apoptosis.

## Data Availability Statement

The original contributions presented in the study are included in the article/**Supplementary Material**. Further inquiries can be directed to the corresponding author.

## Author Contributions

Conceptualization, HJ and XZ. Methodology, HJ. Formal analysis, HJ, KL RL, and SX. Investigation, KL, RL, and SX. Writing, HJ and XZ. Visualization and supervision, XZ. All authors contributed to the article and approved the submitted version.

## Funding

This work was supported by the grant from the Subject of Integrated Traditional Chinese and Western Medicine in Zhejiang Province (2017-XK-A42) and Health Commission of Zhejiang Province (WKJ-ZJ-2036).

## Conflict of Interest

The authors declare that the research was conducted in the absence of any commercial or financial relationships that could be construed as a potential conflict of interest.

## Publisher’s Note

All claims expressed in this article are solely those of the authors and do not necessarily represent those of their affiliated organizations, or those of the publisher, the editors and the reviewers. Any product that may be evaluated in this article, or claim that may be made by its manufacturer, is not guaranteed or endorsed by the publisher.
