# Supplementary figures and images for: Prediction of the Mechanisms by Which Quercetin Enhances Cisplatin Action in Cervical Cancer: A Network Pharmacology Study and Experimental Validation
Source: Front Oncol. 2022 Jan 6;11:780387. doi: 10.3389/fonc.2021.780387 (PMC8770278; doi:10.3389/fonc.2021.780387)

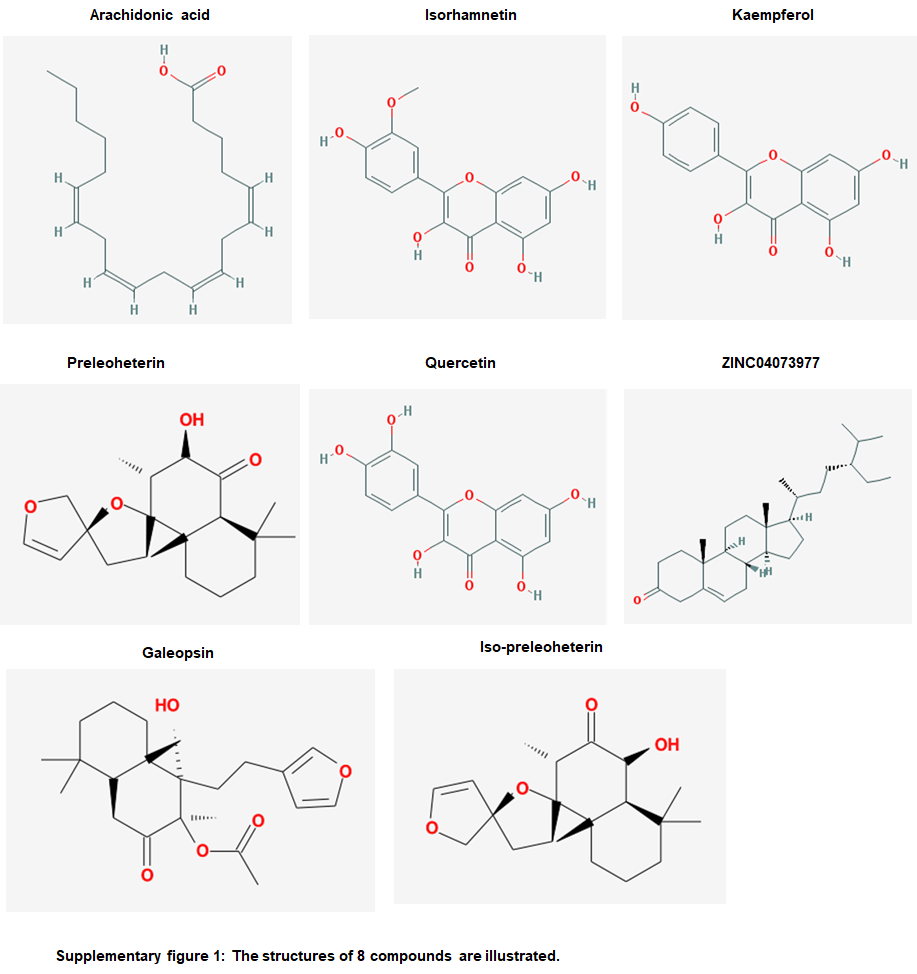

Supplement: Supplementary file 1 [file Image_1.tif]
