# Supplementary material for: Prediction of the Mechanisms by Which Quercetin Enhances Cisplatin Action in Cervical Cancer: A Network Pharmacology Study and Experimental Validation
Source: Front Oncol. 2022 Jan 6;11:780387. doi: 10.3389/fonc.2021.780387 (PMC8770278; doi:10.3389/fonc.2021.780387)
Supplement: Supplementary file 2 [file Table_1.docx]

Supplementary Table 1: A total of 42 compounds in Yimucao retrieved from database.

| MOL ID | Molecule name |
| --- | --- |
| MOL000100 | isoquercetin_qt |
| MOL000108 | quercetin 3-o-rhamnopyranosyl |
| MOL000111 | quercetin,3-o-rutinoside |
| MOL000112 | quercetin-3-o-galactopyranoside |
| MOL001179 | (-)-Alloaromadendrene |
| MOL000141 | hydroxytyrosol |
| MOL001418 | galeopsin |
| MOL000008 | apigenin |
| MOL001420 | ZINC04073977 |
| MOL001421 | preleoheterin |
| MOL001422 | iso-preleoheterin |
| MOL001423 | isorhamnetin-3-o-beta-d-rutinoside |
| MOL001424 | isorhamnetin,3-o-rutinoside |
| MOL001427 | leojaponin |
| MOL001429 | 2,6-Dimethoxyquinol |
| MOL001430 | leonuriside,b |
| MOL001431 | melatonin |
| MOL000098 | quercetin |
| MOL001433 | isoleosibirin |
| MOL001436 | leonuride |
| MOL001437 | leonurinine_qt |
| MOL001438 | leosibiricin |
| MOL001439 | arachidonic acid |
| MOL001600 | copaene |
| MOL001441 | 4-Guanidinobutanoate |
| MOL001442 | phytol |
| MOL001444 | LC 5504 |
| MOL000024 | alpha-humulene |
| MOL000266 | beta-Cubebene |
| MOL000305 | lauric acid |
| MOL000354 | isorhamnetin |
| MOL000036 | beta-caryophyllene |
| MOL000037 | γ-elemene |
| MOL000422 | kaempferol |
| MOL000447 | stachydrine |
| MOL000474 | (-)-Epoxycaryophyllene |
| MOL000602 | FUM |
| MOL000069 | palmitic acid |
| MOL000879 | methyl palmitate |
| MOL000920 | LINALOOL (D) |
| MOL004368 | Hyperin |
| MOL000437 | Hirsutrin |

Supplementary Table 2: A list of potential target genes of Yimucao.

| Compound name | Target genes |
| --- | --- |
| quercetin | *PTGS1, AR, NCOA2, AKR1B1, PRSS1, F7, ACHE, GABRA1, RELA, EGFR, VEGFA, CCND1, BCL2, FOS, EIF6, CASP9, PLAU, RB1, IL6, AHSA1, CASP3, TP63, ELK1, NFKBIA, POR, CASP8, RAF1, PRKCA , IF1A, RUNX1T1, ERBB2, PPARG, ACACA, CYP3A4, CAV1, MYC, CYP1A1, ICAM1, SELE, VCAM1, PTGER3, BIRC5, DUOX2, HSPB1, CYP1B1, CCNB1, ALOX5, GSTP1, NFE2L2, NQO1, PARP1, AHR, PSMD3, COL3A1, DCAF5, NR1I3, CHEK2, HSF1, CRP, RUNX2, RASSF1, CTSD, IGFBP3, IGF2, IRF1, ERBB3, PON1, DIO1, NPEPPS, HK2, RASA1, GSTM1, GSTM2* |
| galeopsin | *CHRM1, ACHE, CHRM2* |
| ZINC04073977 | *PGR, GABRA1* |
| preleoheterin | *CHRM3, CHRM1* |
| iso-preleoheterin | *PTGS1, CHRM1, GABRA1, GRIA2* |
| arachidonic acid | *PTGS1, TRPV1, RXRG, RELA, CCND1, CASP3, PPARG, TNFRSF1A, ALOX5, SELP, GLB1, ABCA1, UCP2, C1R, CETP, ABCG1, ABCC4, KCNK2, COL1A2* |
| isorhamnetin | *PTGS1, ESR1, AR, PPARG, ESR2, GSK3B, PRSS1, NCOA2, CHEK1, AKR1B1, NCOA1, F7, ACHE, GABRA1, GRIA2, RELA, OLR1* |
| kaempferol | *PTGS1, AR, PPARG, NCOA2, PRSS1, PGR, CHRM1, ACHE, CHRM2, GABRA1, F7, RELA, IKBKB, BCL2, AHSA1, CASP3, MAPK8, PPARG, CYP3A4, CYP1A1, ICAM1, SELE, VCAM1, CYP1B1, ALOX5, GSTP1, AHR, PSMD3, NR1I3, DIO1, GSTM1, GSTM2, AKR1C3* |

Supplementary Table 3: Target genes of quercetin and cisplatin for treating cervical cancer.

| No. | Gene |
| --- | --- |
| 1 | *EGFR* |
| 2 | *ERBB2* |
| 3 | *RB1* |
| 4 | *VEGFA* |
| 5 | *CCND1* |
| 6 | *MYC* |
| 7 | *IL6* |
| 8 | *CASP8* |
| 9 | *TP63* |
| 10 | *CHEK2* |
| 11 | *NFE2L2* |
| 12 | *BCL2* |
| 13 | *HSPB1* |
| 14 | *CASP3* |
| 15 | *HIF1A* |
| 16 | *BIRC5* |
| 17 | *GSTM1* |
| 18 | *RAF1* |
| 19 | *PPARG* |
| 20 | *GSTP1* |
| 21 | *RELA* |
| 22 | *CYP1A1* |
| 23 | *PARP1* |
| 24 | *RASSF1* |
| 25 | *IGFBP3* |
| 26 | *CASP9* |
| 27 | *CAV1* |
| 28 | *CCNB1* |
| 29 | *RUNX2* |
| 30 | *PLAU* |
| 31 | *FOS* |
| 32 | *CYP1B1* |
| 33 | *NQO1* |
| 34 | *ICAM1* |
| 35 | *AHR* |
| 36 | *PRKCA* |
| 37 | *COL3A1* |
| 38 | *PTGS1* |
| 39 | *CYP3A4* |
| 40 | *VCAM1* |
| 41 | *PTGER3* |
| 42 | *AKR1B1* |
| 43 | *ACHE* |

Supplemental table 4. GO terms of 43 common target genes of quercetin, cisplatin, and cervical cancer.

| ONTOLOGY | | ID | Description | GeneRatio | BgRatio | pvalue | p.adjust | qvalue | geneID | Count |
| --- | --- | --- | --- | --- | --- | --- | --- | --- | --- | --- |
| BP | GO:0010038 | | response to metal ion | 16/43 | 364/18670 | 5.16E-17 | 7.76E-14 | 3.51E-14 | EGFR/CCND1/BCL2/FOS/CASP9/CASP3/CASP8/HIF1A/CAV1/CYP1A1/ICAM1/VCAM1/CCNB1/NFE2L2/NQO1/PARP1 | 16 |
| BP | GO:0009410 | | response to xenobiotic stimulus | 15/43 | 292/18670 | 5.85E-17 | 7.76E-14 | 3.51E-14 | PTGS1/PPARG/RELA/EGFR/CASP9/RB1/CYP3A4/CYP1A1/ICAM1/CYP1B1/GSTP1/NQO1/AHR/CHEK2/GSTM1 | 15 |
| BP | GO:0071466 | | cellular response to xenobiotic stimulus | 13/43 | 180/18670 | 1.14E-16 | 1.01E-13 | 4.56E-14 | PTGS1/EGFR/CASP9/RB1/CYP3A4/CYP1A1/ICAM1/CYP1B1/GSTP1/NQO1/AHR/CHEK2/GSTM1 | 13 |
| BP | GO:0046677 | | response to antibiotic | 15/43 | 327/18670 | 3.16E-16 | 2.10E-13 | 9.49E-14 | RELA/CCND1/BCL2/CASP9/IL6/CASP3/CASP8/CYP1A1/ICAM1/VCAM1/CYP1B1/GSTP1/NFE2L2/NQO1/AHR | 15 |
| BP | GO:0048545 | | response to steroid hormone | 15/43 | 385/18670 | 3.54E-15 | 1.88E-12 | 8.50E-13 | PPARG/RELA/EGFR/CCND1/BCL2/FOS/CASP9/RB1/IL6/CASP3/TP63/CAV1/ICAM1/GSTP1/PARP1 | 15 |
| BP | GO:0070482 | | response to oxygen levels | 14/43 | 394/18670 | 1.24E-13 | 5.49E-11 | 2.48E-11 | PPARG/VEGFA/BCL2/PLAU/CASP3/RAF1/HIF1A/CAV1/MYC/CYP1A1/ICAM1/VCAM1/CCNB1/NFE2L2 | 14 |
| BP | GO:1901654 | | response to ketone | 11/43 | 193/18670 | 4.66E-13 | 1.77E-10 | 7.99E-11 | PPARG/AKR1B1/RELA/EGFR/CCND1/FOS/CASP9/CAV1/ICAM1/PARP1/AHR | 11 |
| BP | GO:0009314 | | response to radiation | 14/43 | 448/18670 | 7.13E-13 | 2.28E-10 | 1.03E-10 | RELA/EGFR/CCND1/BCL2/FOS/CASP9/CASP3/HIF1A/MYC/ICAM1/VCAM1/PARP1/COL3A1/CHEK2 | 14 |
| BP | GO:0006979 | | response to oxidative stress | 14/43 | 451/18670 | 7.80E-13 | 2.28E-10 | 1.03E-10 | PTGS1/RELA/EGFR/BCL2/FOS/IL6/CASP3/HIF1A/HSPB1/CYP1B1/GSTP1/NFE2L2/NQO1/PARP1 | 14 |
| BP | GO:0001666 | | response to hypoxia | 13/43 | 359/18670 | 8.60E-13 | 2.28E-10 | 1.03E-10 | VEGFA/BCL2/PLAU/CASP3/RAF1/HIF1A/CAV1/MYC/CYP1A1/ICAM1/VCAM1/CCNB1/NFE2L2 | 13 |
| BP | GO:0036293 | | response to decreased oxygen levels | 13/43 | 370/18670 | 1.26E-12 | 3.04E-10 | 1.37E-10 | VEGFA/BCL2/PLAU/CASP3/RAF1/HIF1A/CAV1/MYC/CYP1A1/ICAM1/VCAM1/CCNB1/NFE2L2 | 13 |
| BP | GO:0034599 | | cellular response to oxidative stress | 12/43 | 302/18670 | 2.53E-12 | 5.59E-10 | 2.53E-10 | RELA/EGFR/BCL2/FOS/IL6/HIF1A/HSPB1/CYP1B1/GSTP1/NFE2L2/NQO1/PARP1 | 12 |
| BP | GO:0031960 | | response to corticosteroid | 10/43 | 162/18670 | 2.74E-12 | 5.59E-10 | 2.53E-10 | EGFR/CCND1/BCL2/FOS/CASP9/IL6/CASP3/ICAM1/GSTP1/PARP1 | 10 |
| BP | GO:2001233 | | regulation of apoptotic signaling pathway | 13/43 | 406/18670 | 4.07E-12 | 7.71E-10 | 3.48E-10 | RELA/BCL2/RB1/TP63/CASP8/RAF1/HIF1A/CAV1/ICAM1/HSPB1/GSTP1/NFE2L2/PARP1 | 13 |
| BP | GO:0097193 | | intrinsic apoptotic signaling pathway | 11/43 | 289/18670 | 3.75E-11 | 6.36E-09 | 2.87E-09 | BCL2/CASP9/CASP3/TP63/HIF1A/CAV1/HSPB1/CYP1B1/NFE2L2/PARP1/CHEK2 | 11 |
| BP | GO:0051384 | | response to glucocorticoid | 9/43 | 146/18670 | 3.84E-11 | 6.36E-09 | 2.87E-09 | EGFR/CCND1/BCL2/FOS/CASP9/IL6/CASP3/ICAM1/GSTP1 | 9 |
| BP | GO:2001234 | | negative regulation of apoptotic signaling pathway | 10/43 | 230/18670 | 8.89E-11 | 1.39E-08 | 6.27E-09 | RELA/BCL2/RB1/CASP8/RAF1/HIF1A/ICAM1/HSPB1/GSTP1/NFE2L2 | 10 |
| BP | GO:0000302 | | response to reactive oxygen species | 10/43 | 232/18670 | 9.68E-11 | 1.41E-08 | 6.37E-09 | RELA/EGFR/BCL2/FOS/IL6/CASP3/CYP1B1/GSTP1/NFE2L2/NQO1 | 10 |
| BP | GO:0097305 | | response to alcohol | 10/43 | 233/18670 | 1.01E-10 | 1.41E-08 | 6.37E-09 | PPARG/CCND1/FOS/CASP8/ICAM1/VCAM1/GSTP1/NQO1/PARP1/AHR | 10 |
| BP | GO:0007568 | | aging | 11/43 | 321/18670 | 1.15E-10 | 1.53E-08 | 6.91E-09 | RELA/BCL2/FOS/CASP9/TP63/CYP1A1/ICAM1/VCAM1/NFE2L2/NQO1/CHEK2 | 11 |
| BP | GO:0032496 | | response to lipopolysaccharide | 11/43 | 330/18670 | 1.55E-10 | 1.95E-08 | 8.84E-09 | RELA/FOS/CASP9/IL6/CASP3/CASP8/PRKCA/CYP1A1/ICAM1/VCAM1/GSTP1 | 11 |
| BP | GO:0050673 | | epithelial cell proliferation | 12/43 | 434/18670 | 1.71E-10 | 2.06E-08 | 9.32E-09 | PPARG/EGFR/VEGFA/CCND1/RB1/TP63/PRKCA/HIF1A/ERBB2/CAV1/MYC/IGFBP3 | 12 |
| BP | GO:0001101 | | response to acid chemical | 11/43 | 343/18670 | 2.33E-10 | 2.58E-08 | 1.17E-08 | PPARG/AKR1B1/RELA/EGFR/VEGFA/CASP3/ICAM1/CCNB1/GSTP1/NQO1/COL3A1 | 11 |
| BP | GO:0002237 | | response to molecule of bacterial origin | 11/43 | 343/18670 | 2.33E-10 | 2.58E-08 | 1.17E-08 | RELA/FOS/CASP9/IL6/CASP3/CASP8/PRKCA/CYP1A1/ICAM1/VCAM1/GSTP1 | 11 |
| BP | GO:0006805 | | xenobiotic metabolic process | 8/43 | 125/18670 | 3.84E-10 | 4.07E-08 | 1.84E-08 | PTGS1/CYP3A4/CYP1A1/CYP1B1/GSTP1/NQO1/AHR/GSTM1 | 8 |
| BP | GO:0035690 | | cellular response to drug | 11/43 | 369/18670 | 5.06E-10 | 5.16E-08 | 2.33E-08 | RELA/EGFR/CASP9/IL6/MYC/ICAM1/CYP1B1/NFE2L2/NQO1/AHR/CHEK2 | 11 |
| BP | GO:0008631 | | intrinsic apoptotic signaling pathway in response to oxidative stress | 6/43 | 43/18670 | 5.94E-10 | 5.77E-08 | 2.61E-08 | BCL2/HIF1A/HSPB1/CYP1B1/NFE2L2/PARP1 | 6 |
| BP | GO:0048871 | | multicellular organismal homeostasis | 12/43 | 485/18670 | 6.09E-10 | 5.77E-08 | 2.61E-08 | AKR1B1/ACHE/EGFR/VEGFA/BCL2/RB1/IL6/TP63/PRKCA/CAV1/PTGER3/HSPB1 | 12 |
| BP | GO:0050678 | | regulation of epithelial cell proliferation | 11/43 | 378/18670 | 6.53E-10 | 5.92E-08 | 2.68E-08 | PPARG/EGFR/VEGFA/CCND1/RB1/TP63/PRKCA/HIF1A/ERBB2/CAV1/MYC | 11 |
| BP | GO:0048565 | | digestive tract development | 8/43 | 134/18670 | 6.70E-10 | 5.92E-08 | 2.68E-08 | EGFR/BCL2/RB1/TP63/HIF1A/CYP1A1/CCNB1/COL3A1 | 8 |
| BP | GO:0072593 | | reactive oxygen species metabolic process | 10/43 | 284/18670 | 6.97E-10 | 5.96E-08 | 2.70E-08 | EGFR/BCL2/HIF1A/CAV1/CYP1A1/ICAM1/CYP1B1/GSTP1/NFE2L2/NQO1 | 10 |
| BP | GO:0009411 | | response to UV | 8/43 | 141/18670 | 1.01E-09 | 8.33E-08 | 3.77E-08 | RELA/EGFR/CCND1/BCL2/CASP9/CASP3/MYC/PARP1 | 8 |
| BP | GO:1901522 | | positive regulation of transcription from RNA polymerase II promoter involved in cellular response to chemical stimulus | 5/43 | 22/18670 | 1.30E-09 | 1.03E-07 | 4.68E-08 | RELA/VEGFA/HIF1A/NFE2L2/RUNX2 | 5 |
| BP | GO:0055123 | | digestive system development | 8/43 | 146/18670 | 1.33E-09 | 1.03E-07 | 4.68E-08 | EGFR/BCL2/RB1/TP63/HIF1A/CYP1A1/CCNB1/COL3A1 | 8 |
| BP | GO:0010212 | | response to ionizing radiation | 8/43 | 147/18670 | 1.40E-09 | 1.06E-07 | 4.80E-08 | CCND1/BCL2/CASP3/MYC/ICAM1/VCAM1/PARP1/CHEK2 | 8 |
| BP | GO:0097191 | | extrinsic apoptotic signaling pathway | 9/43 | 224/18670 | 1.74E-09 | 1.28E-07 | 5.78E-08 | RELA/BCL2/CASP9/CASP3/CASP8/RAF1/CAV1/ICAM1/GSTP1 | 9 |
| BP | GO:0030099 | | myeloid cell differentiation | 11/43 | 416/18670 | 1.79E-09 | 1.28E-07 | 5.79E-08 | PPARG/VEGFA/FOS/CASP9/RB1/CASP3/CASP8/PRKCA/HIF1A/MYC/PARP1 | 11 |
| BP | GO:0009416 | | response to light stimulus | 10/43 | 314/18670 | 1.84E-09 | 1.28E-07 | 5.81E-08 | RELA/EGFR/CCND1/BCL2/FOS/CASP9/CASP3/HIF1A/MYC/PARP1 | 10 |
| BP | GO:0018209 | | peptidyl-serine modification | 10/43 | 322/18670 | 2.35E-09 | 1.59E-07 | 7.21E-08 | EGFR/VEGFA/BCL2/IL6/RAF1/PRKCA/CAV1/CCNB1/PARP1/CHEK2 | 10 |
| BP | GO:0071453 | | cellular response to oxygen levels | 9/43 | 234/18670 | 2.55E-09 | 1.69E-07 | 7.64E-08 | PPARG/VEGFA/BCL2/HIF1A/CAV1/MYC/ICAM1/CCNB1/NFE2L2 | 9 |
| BP | GO:0060249 | | anatomical structure homeostasis | 11/43 | 437/18670 | 2.99E-09 | 1.93E-07 | 8.74E-08 | AKR1B1/EGFR/VEGFA/BCL2/RB1/IL6/PRKCA/HIF1A/MYC/HSPB1/PARP1 | 11 |
| BP | GO:0070997 | | neuron death | 10/43 | 348/18670 | 4.94E-09 | 3.12E-07 | 1.41E-07 | BCL2/FOS/CASP9/RB1/CASP3/TP63/CASP8/HIF1A/NQO1/PARP1 | 10 |
| BP | GO:0010039 | | response to iron ion | 5/43 | 33/18670 | 1.15E-08 | 7.11E-07 | 3.22E-07 | CCND1/BCL2/HIF1A/CYP1A1/CCNB1 | 5 |
| BP | GO:0031667 | | response to nutrient levels | 11/43 | 499/18670 | 1.18E-08 | 7.13E-07 | 3.22E-07 | PPARG/RELA/EGFR/CCND1/BCL2/CYP1A1/ICAM1/VCAM1/GSTP1/NFE2L2/NQO1 | 11 |
| BP | GO:2000377 | | regulation of reactive oxygen species metabolic process | 8/43 | 195/18670 | 1.30E-08 | 7.66E-07 | 3.47E-07 | EGFR/BCL2/HIF1A/CAV1/ICAM1/CYP1B1/GSTP1/NFE2L2 | 8 |
| BP | GO:0002573 | | myeloid leukocyte differentiation | 8/43 | 204/18670 | 1.85E-08 | 1.07E-06 | 4.82E-07 | PPARG/VEGFA/FOS/RB1/CASP8/PRKCA/MYC/PARP1 | 8 |
| BP | GO:0050679 | | positive regulation of epithelial cell proliferation | 8/43 | 206/18670 | 2.00E-08 | 1.13E-06 | 5.09E-07 | EGFR/VEGFA/CCND1/TP63/PRKCA/HIF1A/ERBB2/MYC | 8 |
| BP | GO:0018105 | | peptidyl-serine phosphorylation | 9/43 | 299/18670 | 2.15E-08 | 1.18E-06 | 5.33E-07 | EGFR/VEGFA/BCL2/IL6/RAF1/PRKCA/CAV1/CCNB1/CHEK2 | 9 |
| BP | GO:0032355 | | response to estradiol | 7/43 | 134/18670 | 2.18E-08 | 1.18E-06 | 5.33E-07 | EGFR/CCND1/CASP9/CASP3/CASP8/GSTP1/NQO1 | 7 |
| BP | GO:0009612 | | response to mechanical stimulus | 8/43 | 210/18670 | 2.32E-08 | 1.23E-06 | 5.56E-07 | PPARG/RELA/EGFR/FOS/CASP8/RAF1/CCNB1/COL3A1 | 8 |
| BP | GO:0043281 | | regulation of cysteine-type endopeptidase activity involved in apoptotic process | 8/43 | 215/18670 | 2.78E-08 | 1.45E-06 | 6.55E-07 | PPARG/VEGFA/CASP9/TP63/CASP8/RAF1/MYC/BIRC5 | 8 |
| BP | GO:0007584 | | response to nutrient | 8/43 | 219/18670 | 3.21E-08 | 1.63E-06 | 7.39E-07 | PPARG/RELA/EGFR/CCND1/CYP1A1/VCAM1/GSTP1/NQO1 | 8 |
| BP | GO:0034349 | | glial cell apoptotic process | 4/43 | 15/18670 | 3.27E-08 | 1.63E-06 | 7.39E-07 | CASP9/RB1/CASP3/PRKCA | 4 |
| BP | GO:0048608 | | reproductive structure development | 10/43 | 431/18670 | 3.76E-08 | 1.85E-06 | 8.34E-07 | PPARG/EGFR/VEGFA/CCND1/BCL2/CASP3/TP63/CASP8/HIF1A/ICAM1 | 10 |
| BP | GO:0042542 | | response to hydrogen peroxide | 7/43 | 146/18670 | 3.94E-08 | 1.87E-06 | 8.44E-07 | RELA/BCL2/IL6/CASP3/CYP1B1/NFE2L2/NQO1 | 7 |
| BP | GO:0048732 | | gland development | 10/43 | 434/18670 | 4.01E-08 | 1.87E-06 | 8.44E-07 | RELA/EGFR/VEGFA/CCND1/BCL2/TP63/RAF1/HIF1A/CAV1/CYP1A1 | 10 |
| BP | GO:0061458 | | reproductive system development | 10/43 | 434/18670 | 4.01E-08 | 1.87E-06 | 8.44E-07 | PPARG/EGFR/VEGFA/CCND1/BCL2/CASP3/TP63/CASP8/HIF1A/ICAM1 | 10 |
| BP | GO:0001894 | | tissue homeostasis | 8/43 | 227/18670 | 4.24E-08 | 1.94E-06 | 8.77E-07 | AKR1B1/EGFR/VEGFA/BCL2/RB1/IL6/PRKCA/HSPB1 | 8 |
| BP | GO:0030225 | | macrophage differentiation | 5/43 | 44/18670 | 5.18E-08 | 2.33E-06 | 1.05E-06 | VEGFA/RB1/CASP8/PRKCA/PARP1 | 5 |
| BP | GO:2001236 | | regulation of extrinsic apoptotic signaling pathway | 7/43 | 155/18670 | 5.95E-08 | 2.63E-06 | 1.19E-06 | RELA/BCL2/CASP8/RAF1/CAV1/ICAM1/GSTP1 | 7 |
| BP | GO:0071496 | | cellular response to external stimulus | 9/43 | 339/18670 | 6.32E-08 | 2.66E-06 | 1.20E-06 | PPARG/EGFR/BCL2/FOS/CASP8/ICAM1/VCAM1/GSTP1/NFE2L2 | 9 |
| BP | GO:0051402 | | neuron apoptotic process | 8/43 | 239/18670 | 6.32E-08 | 2.66E-06 | 1.20E-06 | BCL2/CASP9/RB1/CASP3/TP63/HIF1A/NQO1/PARP1 | 8 |
| BP | GO:2000116 | | regulation of cysteine-type endopeptidase activity | 8/43 | 239/18670 | 6.32E-08 | 2.66E-06 | 1.20E-06 | PPARG/VEGFA/CASP9/TP63/CASP8/RAF1/MYC/BIRC5 | 8 |
| BP | GO:0033273 | | response to vitamin | 6/43 | 93/18670 | 6.82E-08 | 2.74E-06 | 1.24E-06 | PPARG/RELA/EGFR/CCND1/CYP1A1/GSTP1 | 6 |
| BP | GO:0036473 | | cell death in response to oxidative stress | 6/43 | 93/18670 | 6.82E-08 | 2.74E-06 | 1.24E-06 | BCL2/HIF1A/HSPB1/CYP1B1/NFE2L2/PARP1 | 6 |
| BP | GO:1901655 | | cellular response to ketone | 6/43 | 93/18670 | 6.82E-08 | 2.74E-06 | 1.24E-06 | PPARG/AKR1B1/EGFR/CASP9/ICAM1/AHR | 6 |
| BP | GO:1901216 | | positive regulation of neuron death | 6/43 | 94/18670 | 7.27E-08 | 2.88E-06 | 1.30E-06 | FOS/CASP9/CASP3/CASP8/NQO1/PARP1 | 6 |
| BP | GO:0097237 | | cellular response to toxic substance | 8/43 | 247/18670 | 8.15E-08 | 3.18E-06 | 1.44E-06 | PTGS1/RELA/IL6/CYP1B1/GSTP1/NFE2L2/NQO1/GSTM1 | 8 |
| BP | GO:0071383 | | cellular response to steroid hormone stimulus | 8/43 | 250/18670 | 8.94E-08 | 3.43E-06 | 1.55E-06 | PPARG/EGFR/CASP9/RB1/TP63/ICAM1/GSTP1/PARP1 | 8 |
| BP | GO:0034614 | | cellular response to reactive oxygen species | 7/43 | 168/18670 | 1.03E-07 | 3.92E-06 | 1.77E-06 | RELA/EGFR/FOS/IL6/CYP1B1/NFE2L2/NQO1 | 7 |
| BP | GO:2001237 | | negative regulation of extrinsic apoptotic signaling pathway | 6/43 | 104/18670 | 1.33E-07 | 4.97E-06 | 2.25E-06 | RELA/BCL2/CASP8/RAF1/ICAM1/GSTP1 | 6 |
| BP | GO:0033138 | | positive regulation of peptidyl-serine phosphorylation | 6/43 | 105/18670 | 1.41E-07 | 5.19E-06 | 2.35E-06 | EGFR/VEGFA/BCL2/IL6/RAF1/CAV1 | 6 |
| BP | GO:0048469 | | cell maturation | 7/43 | 177/18670 | 1.48E-07 | 5.36E-06 | 2.43E-06 | PPARG/VEGFA/BCL2/RB1/HIF1A/CCNB1/RUNX2 | 7 |
| BP | GO:0045765 | | regulation of angiogenesis | 9/43 | 383/18670 | 1.78E-07 | 6.39E-06 | 2.89E-06 | PPARG/VEGFA/IL6/PRKCA/HIF1A/ERBB2/HSPB1/CYP1B1/NFE2L2 | 9 |
| BP | GO:1901653 | | cellular response to peptide | 9/43 | 385/18670 | 1.86E-07 | 6.59E-06 | 2.98E-06 | PPARG/AKR1B1/RELA/CAV1/ICAM1/VCAM1/GSTP1/NFE2L2/PARP1 | 9 |
| BP | GO:2000045 | | regulation of G1/S transition of mitotic cell cycle | 7/43 | 184/18670 | 1.92E-07 | 6.71E-06 | 3.03E-06 | EGFR/CCND1/BCL2/RB1/CYP1A1/CCNB1/CHEK2 | 7 |
| BP | GO:0000082 | | G1/S transition of mitotic cell cycle | 8/43 | 279/18670 | 2.07E-07 | 7.14E-06 | 3.23E-06 | EGFR/CCND1/BCL2/RB1/MYC/CYP1A1/CCNB1/CHEK2 | 8 |
| BP | GO:0043200 | | response to amino acid | 6/43 | 113/18670 | 2.18E-07 | 7.42E-06 | 3.36E-06 | RELA/EGFR/CASP3/ICAM1/GSTP1/COL3A1 | 6 |
| BP | GO:0071248 | | cellular response to metal ion | 7/43 | 190/18670 | 2.39E-07 | 8.03E-06 | 3.63E-06 | EGFR/FOS/CYP1A1/CCNB1/NFE2L2/NQO1/PARP1 | 7 |
| BP | GO:0022407 | | regulation of cell-cell adhesion | 9/43 | 403/18670 | 2.74E-07 | 9.08E-06 | 4.11E-06 | RELA/VEGFA/IL6/CASP3/PRKCA/ERBB2/CAV1/ICAM1/VCAM1 | 9 |
| BP | GO:0044843 | | cell cycle G1/S phase transition | 8/43 | 298/18670 | 3.42E-07 | 1.12E-05 | 5.07E-06 | EGFR/CCND1/BCL2/RB1/MYC/CYP1A1/CCNB1/CHEK2 | 8 |
| BP | GO:1902806 | | regulation of cell cycle G1/S phase transition | 7/43 | 202/18670 | 3.62E-07 | 1.17E-05 | 5.30E-06 | EGFR/CCND1/BCL2/RB1/CYP1A1/CCNB1/CHEK2 | 7 |
| BP | GO:0045471 | | response to ethanol | 6/43 | 125/18670 | 3.97E-07 | 1.27E-05 | 5.74E-06 | CCND1/CASP8/ICAM1/VCAM1/GSTP1/NQO1 | 6 |
| BP | GO:1901342 | | regulation of vasculature development | 9/43 | 422/18670 | 4.04E-07 | 1.27E-05 | 5.76E-06 | PPARG/VEGFA/IL6/PRKCA/HIF1A/ERBB2/HSPB1/CYP1B1/NFE2L2 | 9 |
| BP | GO:1902175 | | regulation of oxidative stress-induced intrinsic apoptotic signaling pathway | 4/43 | 27/18670 | 4.12E-07 | 1.28E-05 | 5.81E-06 | HIF1A/HSPB1/NFE2L2/PARP1 | 4 |
| BP | GO:0071456 | | cellular response to hypoxia | 7/43 | 207/18670 | 4.27E-07 | 1.32E-05 | 5.96E-06 | VEGFA/BCL2/HIF1A/MYC/ICAM1/CCNB1/NFE2L2 | 7 |
| BP | GO:0019748 | | secondary metabolic process | 5/43 | 67/18670 | 4.43E-07 | 1.35E-05 | 6.10E-06 | AKR1B1/BCL2/CYP1A1/CYP1B1/NFE2L2 | 5 |
| BP | GO:0051090 | | regulation of DNA-binding transcription factor activity | 9/43 | 432/18670 | 4.91E-07 | 1.48E-05 | 6.69E-06 | PPARG/RELA/VEGFA/FOS/RB1/IL6/CAV1/ICAM1/CYP1B1 | 9 |
| BP | GO:1901214 | | regulation of neuron death | 8/43 | 313/18670 | 4.97E-07 | 1.48E-05 | 6.69E-06 | BCL2/FOS/CASP9/CASP3/CASP8/HIF1A/NQO1/PARP1 | 8 |
| BP | GO:0036294 | | cellular response to decreased oxygen levels | 7/43 | 217/18670 | 5.88E-07 | 1.71E-05 | 7.74E-06 | VEGFA/BCL2/HIF1A/MYC/ICAM1/CCNB1/NFE2L2 | 7 |
| BP | GO:0071241 | | cellular response to inorganic substance | 7/43 | 217/18670 | 5.88E-07 | 1.71E-05 | 7.74E-06 | EGFR/FOS/CYP1A1/CCNB1/NFE2L2/NQO1/PARP1 | 7 |
| BP | GO:0070663 | | regulation of leukocyte proliferation | 7/43 | 222/18670 | 6.85E-07 | 1.97E-05 | 8.92E-06 | BCL2/IL6/CASP3/ERBB2/VCAM1/GSTP1/AHR | 7 |
| BP | GO:0052547 | | regulation of peptidase activity | 9/43 | 452/18670 | 7.17E-07 | 2.04E-05 | 9.24E-06 | PPARG/VEGFA/CASP9/TP63/CASP8/RAF1/CAV1/MYC/BIRC5 | 9 |
| BP | GO:0033135 | | regulation of peptidyl-serine phosphorylation | 6/43 | 139/18670 | 7.43E-07 | 2.09E-05 | 9.46E-06 | EGFR/VEGFA/BCL2/IL6/RAF1/CAV1 | 6 |
| BP | GO:0071214 | | cellular response to abiotic stimulus | 8/43 | 331/18670 | 7.58E-07 | 2.09E-05 | 9.46E-06 | AKR1B1/EGFR/CASP9/CASP3/CASP8/MYC/PARP1/CHEK2 | 8 |
| BP | GO:0104004 | | cellular response to environmental stimulus | 8/43 | 331/18670 | 7.58E-07 | 2.09E-05 | 9.46E-06 | AKR1B1/EGFR/CASP9/CASP3/CASP8/MYC/PARP1/CHEK2 | 8 |
| BP | GO:0007596 | | blood coagulation | 8/43 | 336/18670 | 8.48E-07 | 2.32E-05 | 1.05E-05 | PLAU/IL6/RAF1/PRKCA/CAV1/HSPB1/NFE2L2/COL3A1 | 8 |
| BP | GO:0042110 | | T cell activation | 9/43 | 464/18670 | 8.91E-07 | 2.41E-05 | 1.09E-05 | BCL2/IL6/CASP3/CASP8/ERBB2/CAV1/ICAM1/VCAM1/RUNX2 | 9 |
| BP | GO:0007599 | | hemostasis | 8/43 | 341/18670 | 9.48E-07 | 2.54E-05 | 1.15E-05 | PLAU/IL6/RAF1/PRKCA/CAV1/HSPB1/NFE2L2/COL3A1 | 8 |
| BP | GO:0050817 | | coagulation | 8/43 | 342/18670 | 9.69E-07 | 2.57E-05 | 1.16E-05 | PLAU/IL6/RAF1/PRKCA/CAV1/HSPB1/NFE2L2/COL3A1 | 8 |
| BP | GO:0043536 | | positive regulation of blood vessel endothelial cell migration | 5/43 | 79/18670 | 1.01E-06 | 2.66E-05 | 1.20E-05 | VEGFA/PRKCA/HIF1A/HSPB1/NFE2L2 | 5 |
| BP | GO:0071236 | | cellular response to antibiotic | 6/43 | 147/18670 | 1.03E-06 | 2.68E-05 | 1.21E-05 | RELA/IL6/CYP1B1/NFE2L2/NQO1/AHR | 6 |
| BP | GO:0033002 | | muscle cell proliferation | 7/43 | 239/18670 | 1.12E-06 | 2.89E-05 | 1.31E-05 | PPARG/AKR1B1/EGFR/IL6/CCNB1/GSTP1/IGFBP3 | 7 |
| BP | GO:0048145 | | regulation of fibroblast proliferation | 5/43 | 83/18670 | 1.30E-06 | 3.30E-05 | 1.49E-05 | PPARG/EGFR/MYC/CCNB1/GSTP1 | 5 |
| BP | GO:0032025 | | response to cobalt ion | 3/43 | 10/18670 | 1.35E-06 | 3.38E-05 | 1.53E-05 | CASP9/CASP3/CASP8 | 3 |
| BP | GO:0033483 | | gas homeostasis | 3/43 | 10/18670 | 1.35E-06 | 3.38E-05 | 1.53E-05 | HIF1A/CAV1/GSTP1 | 3 |
| BP | GO:0014910 | | regulation of smooth muscle cell migration | 5/43 | 84/18670 | 1.38E-06 | 3.38E-05 | 1.53E-05 | BCL2/PLAU/GSTP1/NFE2L2/IGFBP3 | 5 |
| BP | GO:0048144 | | fibroblast proliferation | 5/43 | 84/18670 | 1.38E-06 | 3.38E-05 | 1.53E-05 | PPARG/EGFR/MYC/CCNB1/GSTP1 | 5 |
| BP | GO:0043535 | | regulation of blood vessel endothelial cell migration | 6/43 | 156/18670 | 1.46E-06 | 3.55E-05 | 1.61E-05 | PPARG/VEGFA/PRKCA/HIF1A/HSPB1/NFE2L2 | 6 |
| BP | GO:0045931 | | positive regulation of mitotic cell cycle | 6/43 | 163/18670 | 1.88E-06 | 4.54E-05 | 2.05E-05 | EGFR/CCND1/RB1/PRKCA/CYP1A1/CCNB1 | 6 |
| BP | GO:2001242 | | regulation of intrinsic apoptotic signaling pathway | 6/43 | 165/18670 | 2.02E-06 | 4.76E-05 | 2.15E-05 | BCL2/HIF1A/CAV1/HSPB1/NFE2L2/PARP1 | 6 |
| BP | GO:0014909 | | smooth muscle cell migration | 5/43 | 91/18670 | 2.05E-06 | 4.76E-05 | 2.15E-05 | BCL2/PLAU/GSTP1/NFE2L2/IGFBP3 | 5 |
| BP | GO:0045639 | | positive regulation of myeloid cell differentiation | 5/43 | 91/18670 | 2.05E-06 | 4.76E-05 | 2.15E-05 | FOS/RB1/CASP8/PRKCA/HIF1A | 5 |
| BP | GO:1901992 | | positive regulation of mitotic cell cycle phase transition | 5/43 | 91/18670 | 2.05E-06 | 4.76E-05 | 2.15E-05 | EGFR/CCND1/RB1/CYP1A1/CCNB1 | 5 |
| BP | GO:0042572 | | retinol metabolic process | 4/43 | 41/18670 | 2.32E-06 | 5.31E-05 | 2.40E-05 | AKR1B1/CYP3A4/CYP1A1/CYP1B1 | 4 |
| BP | GO:0048660 | | regulation of smooth muscle cell proliferation | 6/43 | 169/18670 | 2.32E-06 | 5.31E-05 | 2.40E-05 | PPARG/AKR1B1/EGFR/IL6/GSTP1/IGFBP3 | 6 |
| BP | GO:0031668 | | cellular response to extracellular stimulus | 7/43 | 268/18670 | 2.41E-06 | 5.45E-05 | 2.47E-05 | PPARG/BCL2/FOS/ICAM1/VCAM1/GSTP1/NFE2L2 | 7 |
| BP | GO:0048659 | | smooth muscle cell proliferation | 6/43 | 171/18670 | 2.49E-06 | 5.59E-05 | 2.53E-05 | PPARG/AKR1B1/EGFR/IL6/GSTP1/IGFBP3 | 6 |
| BP | GO:0043542 | | endothelial cell migration | 7/43 | 273/18670 | 2.72E-06 | 6.06E-05 | 2.74E-05 | PPARG/VEGFA/PRKCA/HIF1A/HSPB1/CYP1B1/NFE2L2 | 7 |
| BP | GO:0001503 | | ossification | 8/43 | 398/18670 | 3.00E-06 | 6.63E-05 | 3.00E-05 | ACHE/EGFR/BCL2/IL6/TP63/HIF1A/RUNX2/IGFBP3 | 8 |
| BP | GO:0001959 | | regulation of cytokine-mediated signaling pathway | 6/43 | 177/18670 | 3.04E-06 | 6.66E-05 | 3.01E-05 | PPARG/IL6/CASP8/HIF1A/CAV1/GSTP1 | 6 |
| BP | GO:0070301 | | cellular response to hydrogen peroxide | 5/43 | 99/18670 | 3.11E-06 | 6.75E-05 | 3.05E-05 | RELA/IL6/CYP1B1/NFE2L2/NQO1 | 5 |
| BP | GO:0042178 | | xenobiotic catabolic process | 3/43 | 13/18670 | 3.20E-06 | 6.87E-05 | 3.11E-05 | CYP1A1/GSTP1/GSTM1 | 3 |
| BP | GO:0030522 | | intracellular receptor signaling pathway | 7/43 | 280/18670 | 3.21E-06 | 6.87E-05 | 3.11E-05 | PPARG/RELA/RB1/TP63/CASP8/PARP1/AHR | 7 |
| BP | GO:0045785 | | positive regulation of cell adhesion | 8/43 | 403/18670 | 3.29E-06 | 6.98E-05 | 3.16E-05 | RELA/VEGFA/IL6/PRKCA/ERBB2/CAV1/ICAM1/VCAM1 | 8 |
| BP | GO:0043534 | | blood vessel endothelial cell migration | 6/43 | 180/18670 | 3.35E-06 | 7.05E-05 | 3.19E-05 | PPARG/VEGFA/PRKCA/HIF1A/HSPB1/NFE2L2 | 6 |
| BP | GO:0021700 | | developmental maturation | 7/43 | 284/18670 | 3.53E-06 | 7.37E-05 | 3.33E-05 | PPARG/VEGFA/BCL2/RB1/HIF1A/CCNB1/RUNX2 | 7 |
| BP | GO:0014812 | | muscle cell migration | 5/43 | 104/18670 | 3.96E-06 | 8.20E-05 | 3.71E-05 | BCL2/PLAU/GSTP1/NFE2L2/IGFBP3 | 5 |
| BP | GO:0061614 | | pri-miRNA transcription by RNA polymerase II | 4/43 | 47/18670 | 4.05E-06 | 8.30E-05 | 3.75E-05 | PPARG/RELA/FOS/HIF1A | 4 |
| BP | GO:0045651 | | positive regulation of macrophage differentiation | 3/43 | 14/18670 | 4.07E-06 | 8.30E-05 | 3.75E-05 | RB1/CASP8/PRKCA | 3 |
| BP | GO:0042136 | | neurotransmitter biosynthetic process | 5/43 | 106/18670 | 4.35E-06 | 8.74E-05 | 3.95E-05 | ACHE/CAV1/ICAM1/CYP1B1/NQO1 | 5 |
| BP | GO:1901989 | | positive regulation of cell cycle phase transition | 5/43 | 106/18670 | 4.35E-06 | 8.74E-05 | 3.95E-05 | EGFR/CCND1/RB1/CYP1A1/CCNB1 | 5 |
| BP | GO:0007595 | | lactation | 4/43 | 48/18670 | 4.41E-06 | 8.79E-05 | 3.97E-05 | VEGFA/CCND1/HIF1A/CAV1 | 4 |
| BP | GO:0060759 | | regulation of response to cytokine stimulus | 6/43 | 190/18670 | 4.58E-06 | 9.05E-05 | 4.09E-05 | PPARG/IL6/CASP8/HIF1A/CAV1/GSTP1 | 6 |
| BP | GO:0030857 | | negative regulation of epithelial cell differentiation | 4/43 | 49/18670 | 4.79E-06 | 9.21E-05 | 4.16E-05 | VEGFA/CCND1/TP63/CAV1 | 4 |
| BP | GO:0035094 | | response to nicotine | 4/43 | 49/18670 | 4.79E-06 | 9.21E-05 | 4.16E-05 | RELA/BCL2/CASP3/VCAM1 | 4 |
| BP | GO:0048546 | | digestive tract morphogenesis | 4/43 | 49/18670 | 4.79E-06 | 9.21E-05 | 4.16E-05 | EGFR/BCL2/TP63/HIF1A | 4 |
| BP | GO:0051205 | | protein insertion into membrane | 4/43 | 49/18670 | 4.79E-06 | 9.21E-05 | 4.16E-05 | EGFR/BCL2/TP63/CASP8 | 4 |
| BP | GO:0070661 | | leukocyte proliferation | 7/43 | 298/18670 | 4.84E-06 | 9.22E-05 | 4.17E-05 | BCL2/IL6/CASP3/ERBB2/VCAM1/GSTP1/AHR | 7 |
| BP | GO:0052548 | | regulation of endopeptidase activity | 8/43 | 425/18670 | 4.87E-06 | 9.22E-05 | 4.17E-05 | PPARG/VEGFA/CASP9/TP63/CASP8/RAF1/MYC/BIRC5 | 8 |
| BP | GO:0001676 | | long-chain fatty acid metabolic process | 5/43 | 109/18670 | 4.99E-06 | 9.38E-05 | 4.24E-05 | PTGS1/CYP3A4/CYP1A1/CYP1B1/GSTP1 | 5 |
| BP | GO:0001933 | | negative regulation of protein phosphorylation | 8/43 | 429/18670 | 5.22E-06 | 9.67E-05 | 4.37E-05 | RB1/CASP3/CAV1/MYC/HSPB1/CCNB1/GSTP1/IGFBP3 | 8 |
| BP | GO:0016101 | | diterpenoid metabolic process | 5/43 | 110/18670 | 5.22E-06 | 9.67E-05 | 4.37E-05 | AKR1B1/EGFR/CYP3A4/CYP1A1/CYP1B1 | 5 |
| BP | GO:1903037 | | regulation of leukocyte cell-cell adhesion | 7/43 | 304/18670 | 5.52E-06 | 0.000102 | 4.60E-05 | RELA/IL6/CASP3/ERBB2/CAV1/ICAM1/VCAM1 | 7 |
| BP | GO:1990748 | | cellular detoxification | 5/43 | 112/18670 | 5.70E-06 | 0.000104 | 4.71E-05 | PTGS1/GSTP1/NFE2L2/NQO1/GSTM1 | 5 |
| BP | GO:0031099 | | regeneration | 6/43 | 198/18670 | 5.80E-06 | 0.000105 | 4.76E-05 | PPARG/EGFR/CCND1/BCL2/CCNB1/GSTP1 | 6 |
| BP | GO:0046660 | | female sex differentiation | 5/43 | 115/18670 | 6.49E-06 | 0.000117 | 5.29E-05 | VEGFA/BCL2/CASP3/TP63/ICAM1 | 5 |
| BP | GO:0034612 | | response to tumor necrosis factor | 7/43 | 312/18670 | 6.54E-06 | 0.000117 | 5.30E-05 | RELA/CASP3/CASP8/ICAM1/VCAM1/GSTP1/NFE2L2 | 7 |
| BP | GO:0045766 | | positive regulation of angiogenesis | 6/43 | 204/18670 | 6.88E-06 | 0.000122 | 5.54E-05 | VEGFA/PRKCA/HIF1A/HSPB1/CYP1B1/NFE2L2 | 6 |
| BP | GO:0002761 | | regulation of myeloid leukocyte differentiation | 5/43 | 117/18670 | 7.06E-06 | 0.000125 | 5.63E-05 | FOS/RB1/CASP8/PRKCA/MYC | 5 |
| BP | GO:0002763 | | positive regulation of myeloid leukocyte differentiation | 4/43 | 54/18670 | 7.09E-06 | 0.000125 | 5.63E-05 | FOS/RB1/CASP8/PRKCA | 4 |
| BP | GO:0050670 | | regulation of lymphocyte proliferation | 6/43 | 208/18670 | 7.69E-06 | 0.000134 | 6.07E-05 | BCL2/IL6/CASP3/ERBB2/VCAM1/AHR | 6 |
| BP | GO:0032944 | | regulation of mononuclear cell proliferation | 6/43 | 209/18670 | 7.90E-06 | 0.000137 | 6.19E-05 | BCL2/IL6/CASP3/ERBB2/VCAM1/AHR | 6 |
| BP | GO:0006721 | | terpenoid metabolic process | 5/43 | 120/18670 | 7.99E-06 | 0.000138 | 6.22E-05 | AKR1B1/EGFR/CYP3A4/CYP1A1/CYP1B1 | 5 |
| BP | GO:0043523 | | regulation of neuron apoptotic process | 6/43 | 210/18670 | 8.12E-06 | 0.000139 | 6.27E-05 | BCL2/CASP9/CASP3/HIF1A/NQO1/PARP1 | 6 |
| BP | GO:1904951 | | positive regulation of establishment of protein localization | 8/43 | 456/18670 | 8.15E-06 | 0.000139 | 6.27E-05 | ACHE/EGFR/BCL2/IL6/TP63/CASP8/HIF1A/ERBB2 | 8 |
| BP | GO:0010332 | | response to gamma radiation | 4/43 | 56/18670 | 8.21E-06 | 0.000139 | 6.27E-05 | BCL2/MYC/PARP1/CHEK2 | 4 |
| BP | GO:1903409 | | reactive oxygen species biosynthetic process | 5/43 | 122/18670 | 8.66E-06 | 0.000144 | 6.53E-05 | CAV1/CYP1A1/ICAM1/CYP1B1/NQO1 | 5 |
| BP | GO:1905477 | | positive regulation of protein localization to membrane | 5/43 | 122/18670 | 8.66E-06 | 0.000144 | 6.53E-05 | EGFR/BCL2/TP63/CASP8/ERBB2 | 5 |
| BP | GO:2001020 | | regulation of response to DNA damage stimulus | 6/43 | 214/18670 | 9.05E-06 | 0.000147 | 6.67E-05 | EGFR/BCL2/CASP9/MYC/PARP1/CHEK2 | 6 |
| BP | GO:0035994 | | response to muscle stretch | 3/43 | 18/18670 | 9.06E-06 | 0.000147 | 6.67E-05 | RELA/FOS/RAF1 | 3 |
| BP | GO:0060749 | | mammary gland alveolus development | 3/43 | 18/18670 | 9.06E-06 | 0.000147 | 6.67E-05 | VEGFA/CCND1/HIF1A | 3 |
| BP | GO:0061377 | | mammary gland lobule development | 3/43 | 18/18670 | 9.06E-06 | 0.000147 | 6.67E-05 | VEGFA/CCND1/HIF1A | 3 |
| BP | GO:0071385 | | cellular response to glucocorticoid stimulus | 4/43 | 58/18670 | 9.45E-06 | 0.000153 | 6.91E-05 | EGFR/CASP9/ICAM1/GSTP1 | 4 |
| BP | GO:2000134 | | negative regulation of G1/S transition of mitotic cell cycle | 5/43 | 125/18670 | 9.75E-06 | 0.000157 | 7.08E-05 | CCND1/BCL2/RB1/CCNB1/CHEK2 | 5 |
| BP | GO:0042326 | | negative regulation of phosphorylation | 8/43 | 468/18670 | 9.85E-06 | 0.000157 | 7.11E-05 | RB1/CASP3/CAV1/MYC/HSPB1/CCNB1/GSTP1/IGFBP3 | 8 |
| BP | GO:0010001 | | glial cell differentiation | 6/43 | 218/18670 | 1.01E-05 | 0.00016 | 7.22E-05 | PPARG/RELA/EGFR/IL6/ERBB2/GSTP1 | 6 |
| BP | GO:0030336 | | negative regulation of cell migration | 7/43 | 334/18670 | 1.02E-05 | 0.000161 | 7.28E-05 | PPARG/BCL2/CYP1B1/GSTP1/NFE2L2/COL3A1/IGFBP3 | 7 |
| BP | GO:0002526 | | acute inflammatory response | 6/43 | 220/18670 | 1.06E-05 | 0.000166 | 7.51E-05 | PPARG/IL6/ICAM1/VCAM1/PTGER3/GSTP1 | 6 |
| BP | GO:1902176 | | negative regulation of oxidative stress-induced intrinsic apoptotic signaling pathway | 3/43 | 19/18670 | 1.07E-05 | 0.000168 | 7.58E-05 | HIF1A/HSPB1/NFE2L2 | 3 |
| BP | GO:0007159 | | leukocyte cell-cell adhesion | 7/43 | 337/18670 | 1.08E-05 | 0.000168 | 7.58E-05 | RELA/IL6/CASP3/ERBB2/CAV1/ICAM1/VCAM1 | 7 |
| BP | GO:0010595 | | positive regulation of endothelial cell migration | 5/43 | 128/18670 | 1.09E-05 | 0.000168 | 7.60E-05 | VEGFA/PRKCA/HIF1A/HSPB1/NFE2L2 | 5 |
| BP | GO:1903706 | | regulation of hemopoiesis | 8/43 | 475/18670 | 1.10E-05 | 0.000168 | 7.60E-05 | FOS/RB1/CASP8/PRKCA/HIF1A/ERBB2/MYC/NFE2L2 | 8 |
| BP | GO:0071384 | | cellular response to corticosteroid stimulus | 4/43 | 61/18670 | 1.16E-05 | 0.000176 | 7.97E-05 | EGFR/CASP9/ICAM1/GSTP1 | 4 |
| BP | GO:0098754 | | detoxification | 5/43 | 131/18670 | 1.22E-05 | 0.000184 | 8.34E-05 | PTGS1/GSTP1/NFE2L2/NQO1/GSTM1 | 5 |
| BP | GO:1902807 | | negative regulation of cell cycle G1/S phase transition | 5/43 | 131/18670 | 1.22E-05 | 0.000184 | 8.34E-05 | CCND1/BCL2/RB1/CCNB1/CHEK2 | 5 |
| BP | GO:0010594 | | regulation of endothelial cell migration | 6/43 | 229/18670 | 1.33E-05 | 0.000199 | 9.01E-05 | PPARG/VEGFA/PRKCA/HIF1A/HSPB1/NFE2L2 | 6 |
| BP | GO:2000146 | | negative regulation of cell motility | 7/43 | 349/18670 | 1.36E-05 | 0.000202 | 9.13E-05 | PPARG/BCL2/CYP1B1/GSTP1/NFE2L2/COL3A1/IGFBP3 | 7 |
| BP | GO:1904018 | | positive regulation of vasculature development | 6/43 | 230/18670 | 1.36E-05 | 0.000202 | 9.13E-05 | VEGFA/PRKCA/HIF1A/HSPB1/CYP1B1/NFE2L2 | 6 |
| BP | GO:0010631 | | epithelial cell migration | 7/43 | 351/18670 | 1.41E-05 | 0.000207 | 9.38E-05 | PPARG/VEGFA/PRKCA/HIF1A/HSPB1/CYP1B1/NFE2L2 | 7 |
| BP | GO:0042445 | | hormone metabolic process | 6/43 | 232/18670 | 1.43E-05 | 0.00021 | 9.49E-05 | AKR1B1/ACHE/HIF1A/CYP3A4/CYP1A1/CYP1B1 | 6 |
| BP | GO:0055093 | | response to hyperoxia | 3/43 | 21/18670 | 1.47E-05 | 0.000214 | 9.69E-05 | PPARG/CAV1/CYP1A1 | 3 |
| BP | GO:0090132 | | epithelium migration | 7/43 | 354/18670 | 1.49E-05 | 0.000215 | 9.74E-05 | PPARG/VEGFA/PRKCA/HIF1A/HSPB1/CYP1B1/NFE2L2 | 7 |
| BP | GO:0007050 | | cell cycle arrest | 6/43 | 237/18670 | 1.62E-05 | 0.000233 | 0.000105 | CCND1/RB1/MYC/CCNB1/CHEK2/RASSF1 | 6 |
| BP | GO:0006720 | | isoprenoid metabolic process | 5/43 | 139/18670 | 1.63E-05 | 0.000234 | 0.000106 | AKR1B1/EGFR/CYP3A4/CYP1A1/CYP1B1 | 5 |
| BP | GO:0090130 | | tissue migration | 7/43 | 360/18670 | 1.66E-05 | 0.000236 | 0.000107 | PPARG/VEGFA/PRKCA/HIF1A/HSPB1/CYP1B1/NFE2L2 | 7 |
| BP | GO:0045649 | | regulation of macrophage differentiation | 3/43 | 22/18670 | 1.70E-05 | 0.000241 | 0.000109 | RB1/CASP8/PRKCA | 3 |
| BP | GO:0001654 | | eye development | 7/43 | 362/18670 | 1.72E-05 | 0.000242 | 0.00011 | ACHE/EGFR/VEGFA/BCL2/HIF1A/CYP1A1/CYP1B1 | 7 |
| BP | GO:0035924 | | cellular response to vascular endothelial growth factor stimulus | 4/43 | 68/18670 | 1.78E-05 | 0.00025 | 0.000113 | RELA/VEGFA/VCAM1/HSPB1 | 4 |
| BP | GO:0150063 | | visual system development | 7/43 | 366/18670 | 1.84E-05 | 0.000257 | 0.000116 | ACHE/EGFR/VEGFA/BCL2/HIF1A/CYP1A1/CYP1B1 | 7 |
| BP | GO:0048872 | | homeostasis of number of cells | 6/43 | 246/18670 | 2.00E-05 | 0.000276 | 0.000125 | VEGFA/BCL2/RB1/IL6/CASP3/HIF1A | 6 |
| BP | GO:0071901 | | negative regulation of protein serine/threonine kinase activity | 5/43 | 145/18670 | 2.00E-05 | 0.000276 | 0.000125 | RB1/CASP3/CAV1/HSPB1/GSTP1 | 5 |
| BP | GO:0048880 | | sensory system development | 7/43 | 371/18670 | 2.01E-05 | 0.000276 | 0.000125 | ACHE/EGFR/VEGFA/BCL2/HIF1A/CYP1A1/CYP1B1 | 7 |
| BP | GO:0002262 | | myeloid cell homeostasis | 5/43 | 147/18670 | 2.14E-05 | 0.000292 | 0.000132 | VEGFA/RB1/IL6/CASP3/HIF1A | 5 |
| BP | GO:2000209 | | regulation of anoikis | 3/43 | 24/18670 | 2.23E-05 | 0.000302 | 0.000137 | BCL2/CAV1/CHEK2 | 3 |
| BP | GO:0045637 | | regulation of myeloid cell differentiation | 6/43 | 251/18670 | 2.24E-05 | 0.000302 | 0.000137 | FOS/RB1/CASP8/PRKCA/HIF1A/MYC | 6 |
| BP | GO:0006801 | | superoxide metabolic process | 4/43 | 73/18670 | 2.37E-05 | 0.000317 | 0.000143 | EGFR/GSTP1/NFE2L2/NQO1 | 4 |
| BP | GO:0031100 | | animal organ regeneration | 4/43 | 73/18670 | 2.37E-05 | 0.000317 | 0.000143 | PPARG/EGFR/CCND1/GSTP1 | 4 |
| BP | GO:0006631 | | fatty acid metabolic process | 7/43 | 383/18670 | 2.47E-05 | 0.000329 | 0.000149 | PTGS1/PPARG/CYP3A4/CAV1/CYP1A1/CYP1B1/GSTP1 | 7 |
| BP | GO:0051271 | | negative regulation of cellular component movement | 7/43 | 384/18670 | 2.51E-05 | 0.00033 | 0.000149 | PPARG/BCL2/CYP1B1/GSTP1/NFE2L2/COL3A1/IGFBP3 | 7 |
| BP | GO:0009404 | | toxin metabolic process | 3/43 | 25/18670 | 2.53E-05 | 0.00033 | 0.000149 | CYP1A1/CYP1B1/NFE2L2 | 3 |
| BP | GO:0060571 | | morphogenesis of an epithelial fold | 3/43 | 25/18670 | 2.53E-05 | 0.00033 | 0.000149 | EGFR/TP63/HIF1A | 3 |
| BP | GO:1904385 | | cellular response to angiotensin | 3/43 | 25/18670 | 2.53E-05 | 0.00033 | 0.000149 | RELA/CAV1/NFE2L2 | 3 |
| BP | GO:0030168 | | platelet activation | 5/43 | 153/18670 | 2.59E-05 | 0.000335 | 0.000152 | IL6/RAF1/PRKCA/HSPB1/COL3A1 | 5 |
| BP | GO:0042133 | | neurotransmitter metabolic process | 5/43 | 153/18670 | 2.59E-05 | 0.000335 | 0.000152 | ACHE/CAV1/ICAM1/CYP1B1/NQO1 | 5 |
| BP | GO:0001960 | | negative regulation of cytokine-mediated signaling pathway | 4/43 | 75/18670 | 2.63E-05 | 0.000337 | 0.000152 | PPARG/IL6/CAV1/GSTP1 | 4 |
| BP | GO:1903201 | | regulation of oxidative stress-induced cell death | 4/43 | 75/18670 | 2.63E-05 | 0.000337 | 0.000152 | HIF1A/HSPB1/NFE2L2/PARP1 | 4 |
| BP | GO:0045787 | | positive regulation of cell cycle | 7/43 | 389/18670 | 2.73E-05 | 0.000347 | 0.000157 | EGFR/CCND1/RB1/PRKCA/CYP1A1/CCNB1/CHEK2 | 7 |
| BP | GO:0051091 | | positive regulation of DNA-binding transcription factor activity | 6/43 | 261/18670 | 2.78E-05 | 0.000353 | 0.00016 | PPARG/RELA/VEGFA/IL6/CAV1/ICAM1 | 6 |
| BP | GO:0032800 | | receptor biosynthetic process | 3/43 | 26/18670 | 2.85E-05 | 0.000357 | 0.000161 | PPARG/ACHE/HIF1A | 3 |
| BP | GO:1900739 | | regulation of protein insertion into mitochondrial membrane involved in apoptotic signaling pathway | 3/43 | 26/18670 | 2.85E-05 | 0.000357 | 0.000161 | BCL2/TP63/CASP8 | 3 |
| BP | GO:1900740 | | positive regulation of protein insertion into mitochondrial membrane involved in apoptotic signaling pathway | 3/43 | 26/18670 | 2.85E-05 | 0.000357 | 0.000161 | BCL2/TP63/CASP8 | 3 |
| BP | GO:0006809 | | nitric oxide biosynthetic process | 4/43 | 77/18670 | 2.92E-05 | 0.000364 | 0.000164 | CAV1/ICAM1/CYP1B1/NQO1 | 4 |
| BP | GO:0040013 | | negative regulation of locomotion | 7/43 | 396/18670 | 3.05E-05 | 0.000378 | 0.000171 | PPARG/BCL2/CYP1B1/GSTP1/NFE2L2/COL3A1/IGFBP3 | 7 |
| BP | GO:0035902 | | response to immobilization stress | 3/43 | 27/18670 | 3.20E-05 | 0.000395 | 0.000179 | PPARG/FOS/CYP1A1 | 3 |
| BP | GO:0030193 | | regulation of blood coagulation | 4/43 | 79/18670 | 3.23E-05 | 0.000397 | 0.000179 | PLAU/PRKCA/CAV1/NFE2L2 | 4 |
| BP | GO:0007548 | | sex differentiation | 6/43 | 270/18670 | 3.37E-05 | 0.000411 | 0.000186 | VEGFA/CCND1/BCL2/CASP3/TP63/ICAM1 | 6 |
| BP | GO:1900046 | | regulation of hemostasis | 4/43 | 80/18670 | 3.40E-05 | 0.000413 | 0.000187 | PLAU/PRKCA/CAV1/NFE2L2 | 4 |
| BP | GO:0046651 | | lymphocyte proliferation | 6/43 | 272/18670 | 3.51E-05 | 0.000423 | 0.000191 | BCL2/IL6/CASP3/ERBB2/VCAM1/AHR | 6 |
| BP | GO:1902105 | | regulation of leukocyte differentiation | 6/43 | 272/18670 | 3.51E-05 | 0.000423 | 0.000191 | FOS/RB1/CASP8/PRKCA/ERBB2/MYC | 6 |
| BP | GO:0060761 | | negative regulation of response to cytokine stimulus | 4/43 | 81/18670 | 3.57E-05 | 0.000426 | 0.000193 | PPARG/IL6/CAV1/GSTP1 | 4 |
| BP | GO:0036296 | | response to increased oxygen levels | 3/43 | 28/18670 | 3.58E-05 | 0.000426 | 0.000193 | PPARG/CAV1/CYP1A1 | 3 |
| BP | GO:1990776 | | response to angiotensin | 3/43 | 28/18670 | 3.58E-05 | 0.000426 | 0.000193 | RELA/CAV1/NFE2L2 | 3 |
| BP | GO:0032943 | | mononuclear cell proliferation | 6/43 | 274/18670 | 3.66E-05 | 0.000433 | 0.000196 | BCL2/IL6/CASP3/ERBB2/VCAM1/AHR | 6 |
| BP | GO:0060537 | | muscle tissue development | 7/43 | 408/18670 | 3.69E-05 | 0.000435 | 0.000197 | VEGFA/BCL2/FOS/RB1/CAV1/CCNB1/COL3A1 | 7 |
| BP | GO:0046209 | | nitric oxide metabolic process | 4/43 | 82/18670 | 3.74E-05 | 0.000439 | 0.000199 | CAV1/ICAM1/CYP1B1/NQO1 | 4 |
| BP | GO:0007517 | | muscle organ development | 7/43 | 410/18670 | 3.81E-05 | 0.000445 | 0.000201 | ACHE/BCL2/FOS/RB1/CAV1/CCNB1/COL3A1 | 7 |
| BP | GO:0010575 | | positive regulation of vascular endothelial growth factor production | 3/43 | 29/18670 | 3.99E-05 | 0.000464 | 0.00021 | IL6/HIF1A/CYP1B1 | 3 |
| BP | GO:0050818 | | regulation of coagulation | 4/43 | 84/18670 | 4.12E-05 | 0.000477 | 0.000216 | PLAU/PRKCA/CAV1/NFE2L2 | 4 |
| BP | GO:0001558 | | regulation of cell growth | 7/43 | 416/18670 | 4.18E-05 | 0.000482 | 0.000218 | PPARG/EGFR/VEGFA/BCL2/RB1/ERBB2/IGFBP3 | 7 |
| BP | GO:0034308 | | primary alcohol metabolic process | 4/43 | 85/18670 | 4.31E-05 | 0.000493 | 0.000223 | AKR1B1/CYP3A4/CYP1A1/CYP1B1 | 4 |
| BP | GO:2001057 | | reactive nitrogen species metabolic process | 4/43 | 85/18670 | 4.31E-05 | 0.000493 | 0.000223 | CAV1/ICAM1/CYP1B1/NQO1 | 4 |
| BP | GO:0010634 | | positive regulation of epithelial cell migration | 5/43 | 171/18670 | 4.42E-05 | 0.000495 | 0.000224 | VEGFA/PRKCA/HIF1A/HSPB1/NFE2L2 | 5 |
| BP | GO:0001782 | | B cell homeostasis | 3/43 | 30/18670 | 4.42E-05 | 0.000495 | 0.000224 | BCL2/CASP3/HIF1A | 3 |
| BP | GO:0001844 | | protein insertion into mitochondrial membrane involved in apoptotic signaling pathway | 3/43 | 30/18670 | 4.42E-05 | 0.000495 | 0.000224 | BCL2/TP63/CASP8 | 3 |
| BP | GO:0048147 | | negative regulation of fibroblast proliferation | 3/43 | 30/18670 | 4.42E-05 | 0.000495 | 0.000224 | PPARG/MYC/GSTP1 | 3 |
| BP | GO:0071549 | | cellular response to dexamethasone stimulus | 3/43 | 30/18670 | 4.42E-05 | 0.000495 | 0.000224 | EGFR/CASP9/ICAM1 | 3 |
| BP | GO:0001776 | | leukocyte homeostasis | 4/43 | 86/18670 | 4.52E-05 | 0.000499 | 0.000226 | BCL2/IL6/CASP3/HIF1A | 4 |
| BP | GO:0001942 | | hair follicle development | 4/43 | 86/18670 | 4.52E-05 | 0.000499 | 0.000226 | RELA/EGFR/BCL2/TP63 | 4 |
| BP | GO:0008625 | | extrinsic apoptotic signaling pathway via death domain receptors | 4/43 | 86/18670 | 4.52E-05 | 0.000499 | 0.000226 | BCL2/CASP8/RAF1/ICAM1 | 4 |
| BP | GO:0051348 | | negative regulation of transferase activity | 6/43 | 285/18670 | 4.55E-05 | 0.000501 | 0.000226 | PPARG/RB1/CASP3/CAV1/HSPB1/GSTP1 | 6 |
| BP | GO:0001659 | | temperature homeostasis | 5/43 | 173/18670 | 4.67E-05 | 0.000511 | 0.000231 | ACHE/VEGFA/RB1/CAV1/PTGER3 | 5 |
| BP | GO:0045737 | | positive regulation of cyclin-dependent protein serine/threonine kinase activity | 3/43 | 31/18670 | 4.89E-05 | 0.000528 | 0.000239 | EGFR/CCND1/CCNB1 | 3 |
| BP | GO:1902895 | | positive regulation of pri-miRNA transcription by RNA polymerase II | 3/43 | 31/18670 | 4.89E-05 | 0.000528 | 0.000239 | RELA/FOS/HIF1A | 3 |
| BP | GO:0022404 | | molting cycle process | 4/43 | 88/18670 | 4.94E-05 | 0.000528 | 0.000239 | RELA/EGFR/BCL2/TP63 | 4 |
| BP | GO:0022405 | | hair cycle process | 4/43 | 88/18670 | 4.94E-05 | 0.000528 | 0.000239 | RELA/EGFR/BCL2/TP63 | 4 |
| BP | GO:0098773 | | skin epidermis development | 4/43 | 88/18670 | 4.94E-05 | 0.000528 | 0.000239 | RELA/EGFR/BCL2/TP63 | 4 |
| BP | GO:1900407 | | regulation of cellular response to oxidative stress | 4/43 | 88/18670 | 4.94E-05 | 0.000528 | 0.000239 | HIF1A/HSPB1/NFE2L2/PARP1 | 4 |
| BP | GO:0042063 | | gliogenesis | 6/43 | 290/18670 | 5.02E-05 | 0.000534 | 0.000241 | PPARG/RELA/EGFR/IL6/ERBB2/GSTP1 | 6 |
| BP | GO:0001936 | | regulation of endothelial cell proliferation | 5/43 | 176/18670 | 5.06E-05 | 0.000537 | 0.000243 | PPARG/VEGFA/PRKCA/HIF1A/CAV1 | 5 |
| BP | GO:0010632 | | regulation of epithelial cell migration | 6/43 | 291/18670 | 5.11E-05 | 0.000538 | 0.000243 | PPARG/VEGFA/PRKCA/HIF1A/HSPB1/NFE2L2 | 6 |
| BP | GO:0071356 | | cellular response to tumor necrosis factor | 6/43 | 291/18670 | 5.11E-05 | 0.000538 | 0.000243 | RELA/CASP8/ICAM1/VCAM1/GSTP1/NFE2L2 | 6 |
| BP | GO:0008210 | | estrogen metabolic process | 3/43 | 32/18670 | 5.39E-05 | 0.000558 | 0.000252 | CYP3A4/CYP1A1/CYP1B1 | 3 |
| BP | GO:0038128 | | ERBB2 signaling pathway | 3/43 | 32/18670 | 5.39E-05 | 0.000558 | 0.000252 | EGFR/PRKCA/ERBB2 | 3 |
| BP | GO:0045907 | | positive regulation of vasoconstriction | 3/43 | 32/18670 | 5.39E-05 | 0.000558 | 0.000252 | EGFR/CAV1/ICAM1 | 3 |
| BP | GO:0051385 | | response to mineralocorticoid | 3/43 | 32/18670 | 5.39E-05 | 0.000558 | 0.000252 | CCND1/FOS/PARP1 | 3 |
| BP | GO:0048771 | | tissue remodeling | 5/43 | 179/18670 | 5.49E-05 | 0.000566 | 0.000256 | EGFR/IL6/PRKCA/HIF1A/CAV1 | 5 |
| BP | GO:0043434 | | response to peptide hormone | 7/43 | 436/18670 | 5.62E-05 | 0.000578 | 0.000261 | PPARG/RELA/CAV1/ICAM1/GSTP1/NFE2L2/PARP1 | 7 |
| BP | GO:0090068 | | positive regulation of cell cycle process | 6/43 | 298/18670 | 5.83E-05 | 0.000597 | 0.00027 | EGFR/CCND1/RB1/CYP1A1/CCNB1/CHEK2 | 6 |
| BP | GO:0036003 | | positive regulation of transcription from RNA polymerase II promoter in response to stress | 3/43 | 33/18670 | 5.92E-05 | 0.000601 | 0.000272 | VEGFA/HIF1A/NFE2L2 | 3 |
| BP | GO:0051204 | | protein insertion into mitochondrial membrane | 3/43 | 33/18670 | 5.92E-05 | 0.000601 | 0.000272 | BCL2/TP63/CASP8 | 3 |
| BP | GO:0007589 | | body fluid secretion | 4/43 | 93/18670 | 6.14E-05 | 0.000621 | 0.000281 | VEGFA/CCND1/HIF1A/CAV1 | 4 |
| BP | GO:1901990 | | regulation of mitotic cell cycle phase transition | 7/43 | 444/18670 | 6.31E-05 | 0.000636 | 0.000287 | EGFR/CCND1/BCL2/RB1/CYP1A1/CCNB1/CHEK2 | 7 |
| BP | GO:1903708 | | positive regulation of hemopoiesis | 5/43 | 185/18670 | 6.42E-05 | 0.000645 | 0.000292 | FOS/RB1/CASP8/PRKCA/HIF1A | 5 |
| BP | GO:0010574 | | regulation of vascular endothelial growth factor production | 3/43 | 34/18670 | 6.48E-05 | 0.000646 | 0.000292 | IL6/HIF1A/CYP1B1 | 3 |
| BP | GO:0043276 | | anoikis | 3/43 | 34/18670 | 6.48E-05 | 0.000646 | 0.000292 | BCL2/CAV1/CHEK2 | 3 |
| BP | GO:1905475 | | regulation of protein localization to membrane | 5/43 | 187/18670 | 6.75E-05 | 0.000671 | 0.000303 | EGFR/BCL2/TP63/CASP8/ERBB2 | 5 |
| BP | GO:0008585 | | female gonad development | 4/43 | 96/18670 | 6.95E-05 | 0.000687 | 0.000311 | VEGFA/BCL2/CASP3/ICAM1 | 4 |
| BP | GO:1901030 | | positive regulation of mitochondrial outer membrane permeabilization involved in apoptotic signaling pathway | 3/43 | 35/18670 | 7.07E-05 | 0.000695 | 0.000314 | BCL2/TP63/CASP8 | 3 |
| BP | GO:1904031 | | positive regulation of cyclin-dependent protein kinase activity | 3/43 | 35/18670 | 7.07E-05 | 0.000695 | 0.000314 | EGFR/CCND1/CCNB1 | 3 |
| BP | GO:1902882 | | regulation of response to oxidative stress | 4/43 | 97/18670 | 7.23E-05 | 0.000707 | 0.00032 | HIF1A/HSPB1/NFE2L2/PARP1 | 4 |
| BP | GO:0042113 | | B cell activation | 6/43 | 310/18670 | 7.26E-05 | 0.000707 | 0.00032 | BCL2/IL6/CASP3/CASP8/VCAM1/AHR | 6 |
| BP | GO:0001935 | | endothelial cell proliferation | 5/43 | 191/18670 | 7.47E-05 | 0.000725 | 0.000328 | PPARG/VEGFA/PRKCA/HIF1A/CAV1 | 5 |
| BP | GO:2001243 | | negative regulation of intrinsic apoptotic signaling pathway | 4/43 | 98/18670 | 7.53E-05 | 0.000728 | 0.000329 | BCL2/HIF1A/HSPB1/NFE2L2 | 4 |
| BP | GO:0043112 | | receptor metabolic process | 5/43 | 192/18670 | 7.65E-05 | 0.000732 | 0.000331 | PPARG/ACHE/VEGFA/HIF1A/CAV1 | 5 |
| BP | GO:0010573 | | vascular endothelial growth factor production | 3/43 | 36/18670 | 7.71E-05 | 0.000732 | 0.000331 | IL6/HIF1A/CYP1B1 | 3 |
| BP | GO:0030224 | | monocyte differentiation | 3/43 | 36/18670 | 7.71E-05 | 0.000732 | 0.000331 | PPARG/VEGFA/MYC | 3 |
| BP | GO:0090322 | | regulation of superoxide metabolic process | 3/43 | 36/18670 | 7.71E-05 | 0.000732 | 0.000331 | EGFR/GSTP1/NFE2L2 | 3 |
| BP | GO:1903131 | | mononuclear cell differentiation | 3/43 | 36/18670 | 7.71E-05 | 0.000732 | 0.000331 | PPARG/VEGFA/MYC | 3 |
| BP | GO:0062012 | | regulation of small molecule metabolic process | 7/43 | 459/18670 | 7.77E-05 | 0.000735 | 0.000332 | PPARG/HIF1A/CAV1/CCNB1/NQO1/PARP1/IGFBP3 | 7 |
| BP | GO:0043010 | | camera-type eye development | 6/43 | 314/18670 | 7.79E-05 | 0.000735 | 0.000332 | ACHE/EGFR/VEGFA/HIF1A/CYP1A1/CYP1B1 | 6 |
| BP | GO:0001667 | | ameboidal-type cell migration | 7/43 | 461/18670 | 7.98E-05 | 0.00075 | 0.000339 | PPARG/VEGFA/PRKCA/HIF1A/HSPB1/CYP1B1/NFE2L2 | 7 |
| BP | GO:0070371 | | ERK1 and ERK2 cascade | 6/43 | 317/18670 | 8.21E-05 | 0.000769 | 0.000348 | EGFR/PRKCA/ERBB2/MYC/ICAM1/GSTP1 | 6 |
| BP | GO:0014912 | | negative regulation of smooth muscle cell migration | 3/43 | 37/18670 | 8.37E-05 | 0.000779 | 0.000352 | GSTP1/NFE2L2/IGFBP3 | 3 |
| BP | GO:0038083 | | peptidyl-tyrosine autophosphorylation | 3/43 | 37/18670 | 8.37E-05 | 0.000779 | 0.000352 | EGFR/VEGFA/CAV1 | 3 |
| BP | GO:0046545 | | development of primary female sexual characteristics | 4/43 | 101/18670 | 8.47E-05 | 0.000785 | 0.000355 | VEGFA/BCL2/CASP3/ICAM1 | 4 |
| BP | GO:0010952 | | positive regulation of peptidase activity | 5/43 | 197/18670 | 8.64E-05 | 0.000798 | 0.000361 | PPARG/CASP9/CASP8/CAV1/MYC | 5 |
| BP | GO:0071375 | | cellular response to peptide hormone stimulus | 6/43 | 321/18670 | 8.79E-05 | 0.000801 | 0.000362 | PPARG/RELA/CAV1/GSTP1/NFE2L2/PARP1 | 6 |
| BP | GO:0000079 | | regulation of cyclin-dependent protein serine/threonine kinase activity | 4/43 | 102/18670 | 8.80E-05 | 0.000801 | 0.000362 | EGFR/CCND1/CASP3/CCNB1 | 4 |
| BP | GO:0098869 | | cellular oxidant detoxification | 4/43 | 102/18670 | 8.80E-05 | 0.000801 | 0.000362 | PTGS1/GSTP1/NFE2L2/NQO1 | 4 |
| BP | GO:2000379 | | positive regulation of reactive oxygen species metabolic process | 4/43 | 102/18670 | 8.80E-05 | 0.000801 | 0.000362 | EGFR/ICAM1/GSTP1/NFE2L2 | 4 |
| BP | GO:0032103 | | positive regulation of response to external stimulus | 6/43 | 323/18670 | 9.10E-05 | 0.000826 | 0.000374 | EGFR/VEGFA/IL6/PRKCA/PTGER3/HSPB1 | 6 |
| BP | GO:1903829 | | positive regulation of cellular protein localization | 6/43 | 324/18670 | 9.26E-05 | 0.000837 | 0.000379 | EGFR/BCL2/TP63/CASP8/ERBB2/PARP1 | 6 |
| BP | GO:0001523 | | retinoid metabolic process | 4/43 | 104/18670 | 9.49E-05 | 0.000848 | 0.000383 | AKR1B1/CYP3A4/CYP1A1/CYP1B1 | 4 |
| BP | GO:0008630 | | intrinsic apoptotic signaling pathway in response to DNA damage | 4/43 | 104/18670 | 9.49E-05 | 0.000848 | 0.000383 | BCL2/CASP9/TP63/CHEK2 | 4 |
| BP | GO:0071887 | | leukocyte apoptotic process | 4/43 | 104/18670 | 9.49E-05 | 0.000848 | 0.000383 | CASP9/IL6/CASP3/HIF1A | 4 |
| BP | GO:0050769 | | positive regulation of neurogenesis | 7/43 | 474/18670 | 9.50E-05 | 0.000848 | 0.000383 | PPARG/RELA/VEGFA/BCL2/IL6/HIF1A/NFE2L2 | 7 |
| BP | GO:1902042 | | negative regulation of extrinsic apoptotic signaling pathway via death domain receptors | 3/43 | 39/18670 | 9.82E-05 | 0.000873 | 0.000395 | CASP8/RAF1/ICAM1 | 3 |
| BP | GO:0030278 | | regulation of ossification | 5/43 | 203/18670 | 9.95E-05 | 0.000882 | 0.000399 | BCL2/IL6/TP63/HIF1A/RUNX2 | 5 |
| BP | GO:1904029 | | regulation of cyclin-dependent protein kinase activity | 4/43 | 106/18670 | 0.000102 | 0.000901 | 0.000408 | EGFR/CCND1/CASP3/CCNB1 | 4 |
| BP | GO:0001655 | | urogenital system development | 6/43 | 330/18670 | 0.000102 | 0.000901 | 0.000408 | AKR1B1/VEGFA/BCL2/CASP9/TP63/MYC | 6 |
| BP | GO:1901987 | | regulation of cell cycle phase transition | 7/43 | 480/18670 | 0.000103 | 0.000902 | 0.000408 | EGFR/CCND1/BCL2/RB1/CYP1A1/CCNB1/CHEK2 | 7 |
| BP | GO:0071222 | | cellular response to lipopolysaccharide | 5/43 | 205/18670 | 0.000104 | 0.000912 | 0.000412 | RELA/IL6/PRKCA/ICAM1/GSTP1 | 5 |
| BP | GO:0045124 | | regulation of bone resorption | 3/43 | 40/18670 | 0.000106 | 0.000921 | 0.000416 | EGFR/IL6/PRKCA | 3 |
| BP | GO:0071548 | | response to dexamethasone | 3/43 | 40/18670 | 0.000106 | 0.000921 | 0.000416 | EGFR/CASP9/ICAM1 | 3 |
| BP | GO:0016049 | | cell growth | 7/43 | 484/18670 | 0.000108 | 0.000934 | 0.000422 | PPARG/EGFR/VEGFA/BCL2/RB1/ERBB2/IGFBP3 | 7 |
| BP | GO:0034284 | | response to monosaccharide | 5/43 | 207/18670 | 0.000109 | 0.000934 | 0.000422 | CASP3/RAF1/HIF1A/ICAM1/GSTP1 | 5 |
| BP | GO:0070555 | | response to interleukin-1 | 5/43 | 207/18670 | 0.000109 | 0.000934 | 0.000422 | RELA/IL6/PRKCA/HIF1A/ICAM1 | 5 |
| BP | GO:0071902 | | positive regulation of protein serine/threonine kinase activity | 6/43 | 334/18670 | 0.000109 | 0.000934 | 0.000422 | EGFR/VEGFA/CCND1/RAF1/ERBB2/CCNB1 | 6 |
| BP | GO:0050727 | | regulation of inflammatory response | 7/43 | 485/18670 | 0.00011 | 0.000934 | 0.000422 | PPARG/RELA/EGFR/RB1/IL6/PTGER3/GSTP1 | 7 |
| BP | GO:0051249 | | regulation of lymphocyte activation | 7/43 | 485/18670 | 0.00011 | 0.000934 | 0.000422 | BCL2/IL6/CASP3/ERBB2/CAV1/VCAM1/AHR | 7 |
| BP | GO:1902893 | | regulation of pri-miRNA transcription by RNA polymerase II | 3/43 | 41/18670 | 0.000114 | 0.000967 | 0.000437 | RELA/FOS/HIF1A | 3 |
| BP | GO:0071229 | | cellular response to acid chemical | 5/43 | 209/18670 | 0.000114 | 0.000967 | 0.000437 | PPARG/EGFR/VEGFA/CCNB1/COL3A1 | 5 |
| BP | GO:0043405 | | regulation of MAP kinase activity | 6/43 | 337/18670 | 0.000115 | 0.000969 | 0.000438 | EGFR/VEGFA/RAF1/ERBB2/CAV1/GSTP1 | 6 |
| BP | GO:0045930 | | negative regulation of mitotic cell cycle | 6/43 | 338/18670 | 0.000117 | 0.000982 | 0.000444 | EGFR/CCND1/BCL2/RB1/CCNB1/CHEK2 | 6 |
| BP | GO:0033559 | | unsaturated fatty acid metabolic process | 4/43 | 110/18670 | 0.000118 | 0.000989 | 0.000447 | PTGS1/CYP1A1/CYP1B1/GSTP1 | 4 |
| BP | GO:0071219 | | cellular response to molecule of bacterial origin | 5/43 | 212/18670 | 0.000122 | 0.001018 | 0.00046 | RELA/IL6/PRKCA/ICAM1/GSTP1 | 5 |
| BP | GO:1904019 | | epithelial cell apoptotic process | 4/43 | 111/18670 | 0.000122 | 0.001018 | 0.00046 | RB1/IL6/ICAM1/NFE2L2 | 4 |
| BP | GO:0042303 | | molting cycle | 4/43 | 112/18670 | 0.000126 | 0.001044 | 0.000472 | RELA/EGFR/BCL2/TP63 | 4 |
| BP | GO:0042633 | | hair cycle | 4/43 | 112/18670 | 0.000126 | 0.001044 | 0.000472 | RELA/EGFR/BCL2/TP63 | 4 |
| BP | GO:0043279 | | response to alkaloid | 4/43 | 112/18670 | 0.000126 | 0.001044 | 0.000472 | PPARG/RELA/CASP3/ICAM1 | 4 |
| BP | GO:0071364 | | cellular response to epidermal growth factor stimulus | 3/43 | 43/18670 | 0.000132 | 0.001081 | 0.000489 | EGFR/ERBB2/GSTP1 | 3 |
| BP | GO:1904646 | | cellular response to amyloid-beta | 3/43 | 43/18670 | 0.000132 | 0.001081 | 0.000489 | ICAM1/VCAM1/PARP1 | 3 |
| BP | GO:0030218 | | erythrocyte differentiation | 4/43 | 114/18670 | 0.000135 | 0.001108 | 0.000501 | VEGFA/RB1/CASP3/HIF1A | 4 |
| BP | GO:0008406 | | gonad development | 5/43 | 217/18670 | 0.000136 | 0.00111 | 0.000502 | VEGFA/CCND1/BCL2/CASP3/ICAM1 | 5 |
| BP | GO:1903039 | | positive regulation of leukocyte cell-cell adhesion | 5/43 | 218/18670 | 0.000139 | 0.001131 | 0.000511 | RELA/IL6/CAV1/ICAM1/VCAM1 | 5 |
| BP | GO:0046688 | | response to copper ion | 3/43 | 44/18670 | 0.000141 | 0.001137 | 0.000514 | CYP1A1/ICAM1/NFE2L2 | 3 |
| BP | GO:0061756 | | leukocyte adhesion to vascular endothelial cell | 3/43 | 44/18670 | 0.000141 | 0.001137 | 0.000514 | RELA/ICAM1/VCAM1 | 3 |
| BP | GO:1901028 | | regulation of mitochondrial outer membrane permeabilization involved in apoptotic signaling pathway | 3/43 | 44/18670 | 0.000141 | 0.001137 | 0.000514 | BCL2/TP63/CASP8 | 3 |
| BP | GO:0007569 | | cell aging | 4/43 | 116/18670 | 0.000145 | 0.001163 | 0.000526 | BCL2/TP63/ICAM1/CHEK2 | 4 |
| BP | GO:0010822 | | positive regulation of mitochondrion organization | 4/43 | 117/18670 | 0.00015 | 0.001199 | 0.000542 | BCL2/TP63/CASP8/HIF1A | 4 |
| BP | GO:0032570 | | response to progesterone | 3/43 | 45/18670 | 0.000151 | 0.001202 | 0.000543 | RELA/FOS/CAV1 | 3 |
| BP | GO:0042771 | | intrinsic apoptotic signaling pathway in response to DNA damage by p53 class mediator | 3/43 | 45/18670 | 0.000151 | 0.001202 | 0.000543 | BCL2/TP63/CHEK2 | 3 |
| BP | GO:0045137 | | development of primary sexual characteristics | 5/43 | 223/18670 | 0.000155 | 0.001227 | 0.000555 | VEGFA/CCND1/BCL2/CASP3/ICAM1 | 5 |
| BP | GO:0001649 | | osteoblast differentiation | 5/43 | 225/18670 | 0.000161 | 0.001268 | 0.000574 | ACHE/IL6/TP63/RUNX2/IGFBP3 | 5 |
| BP | GO:0007160 | | cell-matrix adhesion | 5/43 | 225/18670 | 0.000161 | 0.001268 | 0.000574 | VEGFA/BCL2/PLAU/VCAM1/COL3A1 | 5 |
| BP | GO:0071354 | | cellular response to interleukin-6 | 3/43 | 46/18670 | 0.000161 | 0.001268 | 0.000574 | RELA/IL6/ICAM1 | 3 |
| BP | GO:0022612 | | gland morphogenesis | 4/43 | 120/18670 | 0.000165 | 0.001294 | 0.000585 | EGFR/BCL2/TP63/CAV1 | 4 |
| BP | GO:0070849 | | response to epidermal growth factor | 3/43 | 47/18670 | 0.000172 | 0.001338 | 0.000605 | EGFR/ERBB2/GSTP1 | 3 |
| BP | GO:0018108 | | peptidyl-tyrosine phosphorylation | 6/43 | 363/18670 | 0.000172 | 0.001338 | 0.000605 | EGFR/VEGFA/IL6/ERBB2/CAV1/ICAM1 | 6 |
| BP | GO:0045862 | | positive regulation of proteolysis | 6/43 | 363/18670 | 0.000172 | 0.001338 | 0.000605 | PPARG/CASP9/CASP8/CAV1/MYC/NFE2L2 | 6 |
| BP | GO:0034101 | | erythrocyte homeostasis | 4/43 | 122/18670 | 0.000176 | 0.001363 | 0.000617 | VEGFA/RB1/CASP3/HIF1A | 4 |
| BP | GO:0009743 | | response to carbohydrate | 5/43 | 230/18670 | 0.000179 | 0.00138 | 0.000624 | CASP3/RAF1/HIF1A/ICAM1/GSTP1 | 5 |
| BP | GO:0018212 | | peptidyl-tyrosine modification | 6/43 | 366/18670 | 0.00018 | 0.001387 | 0.000627 | EGFR/VEGFA/IL6/ERBB2/CAV1/ICAM1 | 6 |
| BP | GO:0046850 | | regulation of bone remodeling | 3/43 | 48/18670 | 0.000183 | 0.001407 | 0.000636 | EGFR/IL6/PRKCA | 3 |
| BP | GO:0030198 | | extracellular matrix organization | 6/43 | 368/18670 | 0.000185 | 0.00142 | 0.000642 | RB1/IL6/ICAM1/VCAM1/CYP1B1/COL3A1 | 6 |
| BP | GO:0006469 | | negative regulation of protein kinase activity | 5/43 | 235/18670 | 0.000197 | 0.001503 | 0.00068 | RB1/CASP3/CAV1/HSPB1/GSTP1 | 5 |
| BP | GO:0046777 | | protein autophosphorylation | 5/43 | 235/18670 | 0.000197 | 0.001503 | 0.00068 | EGFR/VEGFA/ERBB2/CAV1/CHEK2 | 5 |
| BP | GO:0071216 | | cellular response to biotic stimulus | 5/43 | 236/18670 | 0.000201 | 0.001529 | 0.000691 | RELA/IL6/PRKCA/ICAM1/GSTP1 | 5 |
| BP | GO:0043620 | | regulation of DNA-templated transcription in response to stress | 4/43 | 127/18670 | 0.000205 | 0.001549 | 0.000701 | RELA/VEGFA/HIF1A/NFE2L2 | 4 |
| BP | GO:0002931 | | response to ischemia | 3/43 | 50/18670 | 0.000207 | 0.001549 | 0.000701 | BCL2/CASP9/CAV1 | 3 |
| BP | GO:0009409 | | response to cold | 3/43 | 50/18670 | 0.000207 | 0.001549 | 0.000701 | PPARG/FOS/CASP8 | 3 |
| BP | GO:0070741 | | response to interleukin-6 | 3/43 | 50/18670 | 0.000207 | 0.001549 | 0.000701 | RELA/IL6/ICAM1 | 3 |
| BP | GO:1900087 | | positive regulation of G1/S transition of mitotic cell cycle | 3/43 | 50/18670 | 0.000207 | 0.001549 | 0.000701 | EGFR/CCND1/CYP1A1 | 3 |
| BP | GO:0034754 | | cellular hormone metabolic process | 4/43 | 129/18670 | 0.000218 | 0.001625 | 0.000735 | AKR1B1/CYP3A4/CYP1A1/CYP1B1 | 4 |
| BP | GO:0019369 | | arachidonic acid metabolic process | 3/43 | 51/18670 | 0.000219 | 0.001625 | 0.000735 | PTGS1/CYP1A1/CYP1B1 | 3 |
| BP | GO:0048146 | | positive regulation of fibroblast proliferation | 3/43 | 51/18670 | 0.000219 | 0.001625 | 0.000735 | EGFR/MYC/CCNB1 | 3 |
| BP | GO:0050999 | | regulation of nitric-oxide synthase activity | 3/43 | 51/18670 | 0.000219 | 0.001625 | 0.000735 | EGFR/HIF1A/CAV1 | 3 |
| BP | GO:0010749 | | regulation of nitric oxide mediated signal transduction | 2/43 | 10/18670 | 0.00023 | 0.001702 | 0.00077 | EGFR/VEGFA | 2 |
| BP | GO:0043280 | | positive regulation of cysteine-type endopeptidase activity involved in apoptotic process | 4/43 | 132/18670 | 0.000238 | 0.001752 | 0.000792 | PPARG/CASP9/CASP8/MYC | 4 |
| BP | GO:1901991 | | negative regulation of mitotic cell cycle phase transition | 5/43 | 248/18670 | 0.000253 | 0.001857 | 0.00084 | CCND1/BCL2/RB1/CCNB1/CHEK2 | 5 |
| BP | GO:0014706 | | striated muscle tissue development | 6/43 | 390/18670 | 0.000254 | 0.001857 | 0.00084 | VEGFA/BCL2/FOS/RB1/CAV1/CCNB1 | 6 |
| BP | GO:0001889 | | liver development | 4/43 | 135/18670 | 0.000259 | 0.001884 | 0.000852 | RELA/EGFR/CCND1/CYP1A1 | 4 |
| BP | GO:0030199 | | collagen fibril organization | 3/43 | 54/18670 | 0.00026 | 0.001884 | 0.000852 | RB1/CYP1B1/COL3A1 | 3 |
| BP | GO:0097345 | | mitochondrial outer membrane permeabilization | 3/43 | 54/18670 | 0.00026 | 0.001884 | 0.000852 | BCL2/TP63/CASP8 | 3 |
| BP | GO:1904645 | | response to amyloid-beta | 3/43 | 54/18670 | 0.00026 | 0.001884 | 0.000852 | ICAM1/VCAM1/PARP1 | 3 |
| BP | GO:0030072 | | peptide hormone secretion | 5/43 | 250/18670 | 0.000263 | 0.001898 | 0.000859 | EGFR/IL6/RAF1/PRKCA/HIF1A | 5 |
| BP | GO:0046425 | | regulation of JAK-STAT cascade | 4/43 | 137/18670 | 0.000274 | 0.001973 | 0.000892 | AKR1B1/IL6/CAV1/CYP1B1 | 4 |
| BP | GO:0001541 | | ovarian follicle development | 3/43 | 55/18670 | 0.000275 | 0.001973 | 0.000892 | VEGFA/BCL2/ICAM1 | 3 |
| BP | GO:0032494 | | response to peptidoglycan | 2/43 | 11/18670 | 0.000281 | 0.002009 | 0.000909 | RELA/IL6 | 2 |
| BP | GO:0043619 | | regulation of transcription from RNA polymerase II promoter in response to oxidative stress | 2/43 | 11/18670 | 0.000281 | 0.002009 | 0.000909 | HIF1A/NFE2L2 | 2 |
| BP | GO:0061008 | | hepaticobiliary system development | 4/43 | 138/18670 | 0.000282 | 0.002009 | 0.000909 | RELA/EGFR/CCND1/CYP1A1 | 4 |
| BP | GO:0022409 | | positive regulation of cell-cell adhesion | 5/43 | 255/18670 | 0.000288 | 0.002046 | 0.000925 | RELA/IL6/CAV1/ICAM1/VCAM1 | 5 |
| BP | GO:0006749 | | glutathione metabolic process | 3/43 | 56/18670 | 0.00029 | 0.002048 | 0.000926 | GSTP1/NFE2L2/GSTM1 | 3 |
| BP | GO:1903202 | | negative regulation of oxidative stress-induced cell death | 3/43 | 56/18670 | 0.00029 | 0.002048 | 0.000926 | HIF1A/HSPB1/NFE2L2 | 3 |
| BP | GO:0050730 | | regulation of peptidyl-tyrosine phosphorylation | 5/43 | 256/18670 | 0.000293 | 0.002067 | 0.000935 | EGFR/VEGFA/IL6/CAV1/ICAM1 | 5 |
| BP | GO:0033673 | | negative regulation of kinase activity | 5/43 | 257/18670 | 0.000298 | 0.002099 | 0.000949 | RB1/CASP3/CAV1/HSPB1/GSTP1 | 5 |
| BP | GO:0043525 | | positive regulation of neuron apoptotic process | 3/43 | 57/18670 | 0.000305 | 0.002136 | 0.000966 | CASP9/CASP3/NQO1 | 3 |
| BP | GO:1900408 | | negative regulation of cellular response to oxidative stress | 3/43 | 57/18670 | 0.000305 | 0.002136 | 0.000966 | HIF1A/HSPB1/NFE2L2 | 3 |
| BP | GO:0019229 | | regulation of vasoconstriction | 3/43 | 58/18670 | 0.000321 | 0.002223 | 0.001006 | EGFR/CAV1/ICAM1 | 3 |
| BP | GO:1902041 | | regulation of extrinsic apoptotic signaling pathway via death domain receptors | 3/43 | 58/18670 | 0.000321 | 0.002223 | 0.001006 | CASP8/RAF1/ICAM1 | 3 |
| BP | GO:1903749 | | positive regulation of establishment of protein localization to mitochondrion | 3/43 | 58/18670 | 0.000321 | 0.002223 | 0.001006 | BCL2/TP63/CASP8 | 3 |
| BP | GO:0030879 | | mammary gland development | 4/43 | 143/18670 | 0.000323 | 0.002223 | 0.001006 | VEGFA/CCND1/HIF1A/CAV1 | 4 |
| BP | GO:0106106 | | cold-induced thermogenesis | 4/43 | 143/18670 | 0.000323 | 0.002223 | 0.001006 | ACHE/VEGFA/RB1/CAV1 | 4 |
| BP | GO:0120161 | | regulation of cold-induced thermogenesis | 4/43 | 143/18670 | 0.000323 | 0.002223 | 0.001006 | ACHE/VEGFA/RB1/CAV1 | 4 |
| BP | GO:1902107 | | positive regulation of leukocyte differentiation | 4/43 | 144/18670 | 0.000332 | 0.002277 | 0.00103 | FOS/RB1/CASP8/PRKCA | 4 |
| BP | GO:0002674 | | negative regulation of acute inflammatory response | 2/43 | 12/18670 | 0.000337 | 0.002287 | 0.001034 | PPARG/GSTP1 | 2 |
| BP | GO:0007077 | | mitotic nuclear envelope disassembly | 2/43 | 12/18670 | 0.000337 | 0.002287 | 0.001034 | PRKCA/CCNB1 | 2 |
| BP | GO:0033197 | | response to vitamin E | 2/43 | 12/18670 | 0.000337 | 0.002287 | 0.001034 | PPARG/CCND1 | 2 |
| BP | GO:0001658 | | branching involved in ureteric bud morphogenesis | 3/43 | 59/18670 | 0.000338 | 0.002287 | 0.001034 | VEGFA/BCL2/MYC | 3 |
| BP | GO:0043388 | | positive regulation of DNA binding | 3/43 | 59/18670 | 0.000338 | 0.002287 | 0.001034 | PPARG/RB1/PARP1 | 3 |
| BP | GO:1902883 | | negative regulation of response to oxidative stress | 3/43 | 59/18670 | 0.000338 | 0.002287 | 0.001034 | HIF1A/HSPB1/NFE2L2 | 3 |
| BP | GO:1904892 | | regulation of STAT cascade | 4/43 | 146/18670 | 0.000349 | 0.002357 | 0.001066 | AKR1B1/IL6/CAV1/CYP1B1 | 4 |
| BP | GO:1902110 | | positive regulation of mitochondrial membrane permeability involved in apoptotic process | 3/43 | 60/18670 | 0.000355 | 0.002381 | 0.001077 | BCL2/TP63/CASP8 | 3 |
| BP | GO:1902808 | | positive regulation of cell cycle G1/S phase transition | 3/43 | 60/18670 | 0.000355 | 0.002381 | 0.001077 | EGFR/CCND1/CYP1A1 | 3 |
| BP | GO:1901988 | | negative regulation of cell cycle phase transition | 5/43 | 267/18670 | 0.000356 | 0.002381 | 0.001077 | CCND1/BCL2/RB1/CCNB1/CHEK2 | 5 |
| BP | GO:0051592 | | response to calcium ion | 4/43 | 148/18670 | 0.000368 | 0.002451 | 0.001108 | EGFR/CCND1/FOS/CAV1 | 4 |
| BP | GO:0061041 | | regulation of wound healing | 4/43 | 148/18670 | 0.000368 | 0.002451 | 0.001108 | PLAU/PRKCA/CAV1/NFE2L2 | 4 |
| BP | GO:0043588 | | skin development | 6/43 | 419/18670 | 0.000372 | 0.002469 | 0.001117 | RELA/EGFR/BCL2/CASP3/TP63/COL3A1 | 6 |
| BP | GO:0030888 | | regulation of B cell proliferation | 3/43 | 61/18670 | 0.000373 | 0.002469 | 0.001117 | BCL2/CASP3/AHR | 3 |
| BP | GO:0045165 | | cell fate commitment | 5/43 | 270/18670 | 0.000374 | 0.002469 | 0.001117 | PPARG/BCL2/IL6/CASP3/RUNX2 | 5 |
| BP | GO:0045927 | | positive regulation of growth | 5/43 | 270/18670 | 0.000374 | 0.002469 | 0.001117 | EGFR/VEGFA/BCL2/ERBB2/CCNB1 | 5 |
| BP | GO:0014074 | | response to purine-containing compound | 4/43 | 149/18670 | 0.000377 | 0.002477 | 0.00112 | PPARG/RELA/FOS/AHR | 4 |
| BP | GO:2001056 | | positive regulation of cysteine-type endopeptidase activity | 4/43 | 149/18670 | 0.000377 | 0.002477 | 0.00112 | PPARG/CASP9/CASP8/MYC | 4 |
| BP | GO:0043062 | | extracellular structure organization | 6/43 | 422/18670 | 0.000386 | 0.002528 | 0.001144 | RB1/IL6/ICAM1/VCAM1/CYP1B1/COL3A1 | 6 |
| BP | GO:0032868 | | response to insulin | 5/43 | 272/18670 | 0.000387 | 0.002528 | 0.001144 | PPARG/RELA/ICAM1/GSTP1/PARP1 | 5 |
| BP | GO:0002260 | | lymphocyte homeostasis | 3/43 | 62/18670 | 0.000391 | 0.002538 | 0.001148 | BCL2/CASP3/HIF1A | 3 |
| BP | GO:0045453 | | bone resorption | 3/43 | 62/18670 | 0.000391 | 0.002538 | 0.001148 | EGFR/IL6/PRKCA | 3 |
| BP | GO:1902686 | | mitochondrial outer membrane permeabilization involved in programmed cell death | 3/43 | 62/18670 | 0.000391 | 0.002538 | 0.001148 | BCL2/TP63/CASP8 | 3 |
| BP | GO:0001890 | | placenta development | 4/43 | 152/18670 | 0.000407 | 0.002632 | 0.00119 | PPARG/EGFR/CASP8/HIF1A | 4 |
| BP | GO:0031571 | | mitotic G1 DNA damage checkpoint | 3/43 | 63/18670 | 0.00041 | 0.002641 | 0.001194 | CCND1/CCNB1/CHEK2 | 3 |
| BP | GO:0044819 | | mitotic G1/S transition checkpoint | 3/43 | 63/18670 | 0.00041 | 0.002641 | 0.001194 | CCND1/CCNB1/CHEK2 | 3 |
| BP | GO:0034250 | | positive regulation of cellular amide metabolic process | 4/43 | 153/18670 | 0.000417 | 0.002679 | 0.001212 | IL6/CASP3/ERBB2/NFE2L2 | 4 |
| BP | GO:0051052 | | regulation of DNA metabolic process | 6/43 | 429/18670 | 0.000422 | 0.0027 | 0.001221 | PPARG/EGFR/IL6/MYC/PARP1/CHEK2 | 6 |
| BP | GO:1990845 | | adaptive thermogenesis | 4/43 | 154/18670 | 0.000428 | 0.002707 | 0.001224 | ACHE/VEGFA/RB1/CAV1 | 4 |
| BP | GO:0001822 | | kidney development | 5/43 | 278/18670 | 0.000428 | 0.002707 | 0.001224 | AKR1B1/VEGFA/BCL2/CASP9/MYC | 5 |
| BP | GO:0032768 | | regulation of monooxygenase activity | 3/43 | 64/18670 | 0.00043 | 0.002707 | 0.001224 | EGFR/HIF1A/CAV1 | 3 |
| BP | GO:0035794 | | positive regulation of mitochondrial membrane permeability | 3/43 | 64/18670 | 0.00043 | 0.002707 | 0.001224 | BCL2/TP63/CASP8 | 3 |
| BP | GO:0044783 | | G1 DNA damage checkpoint | 3/43 | 64/18670 | 0.00043 | 0.002707 | 0.001224 | CCND1/CCNB1/CHEK2 | 3 |
| BP | GO:0045669 | | positive regulation of osteoblast differentiation | 3/43 | 64/18670 | 0.00043 | 0.002707 | 0.001224 | IL6/TP63/RUNX2 | 3 |
| BP | GO:2000378 | | negative regulation of reactive oxygen species metabolic process | 3/43 | 64/18670 | 0.00043 | 0.002707 | 0.001224 | BCL2/HIF1A/CAV1 | 3 |
| BP | GO:0007259 | | JAK-STAT cascade | 4/43 | 156/18670 | 0.000449 | 0.002807 | 0.001269 | AKR1B1/IL6/CAV1/CYP1B1 | 4 |
| BP | GO:0030856 | | regulation of epithelial cell differentiation | 4/43 | 156/18670 | 0.000449 | 0.002807 | 0.001269 | VEGFA/CCND1/TP63/CAV1 | 4 |
| BP | GO:0042129 | | regulation of T cell proliferation | 4/43 | 156/18670 | 0.000449 | 0.002807 | 0.001269 | IL6/CASP3/ERBB2/VCAM1 | 4 |
| BP | GO:0060675 | | ureteric bud morphogenesis | 3/43 | 65/18670 | 0.00045 | 0.002807 | 0.001269 | VEGFA/BCL2/MYC | 3 |
| BP | GO:0036295 | | cellular response to increased oxygen levels | 2/43 | 14/18670 | 0.000463 | 0.002883 | 0.001304 | PPARG/CAV1 | 2 |
| BP | GO:0072171 | | mesonephric tubule morphogenesis | 3/43 | 66/18670 | 0.000471 | 0.002908 | 0.001315 | VEGFA/BCL2/MYC | 3 |
| BP | GO:1902108 | | regulation of mitochondrial membrane permeability involved in apoptotic process | 3/43 | 66/18670 | 0.000471 | 0.002908 | 0.001315 | BCL2/TP63/CASP8 | 3 |
| BP | GO:1905710 | | positive regulation of membrane permeability | 3/43 | 66/18670 | 0.000471 | 0.002908 | 0.001315 | BCL2/TP63/CASP8 | 3 |
| BP | GO:0019932 | | second-messenger-mediated signaling | 6/43 | 439/18670 | 0.000476 | 0.002937 | 0.001328 | EGFR/VEGFA/PRKCA/VCAM1/PTGER3/AHR | 6 |
| BP | GO:0002673 | | regulation of acute inflammatory response | 4/43 | 159/18670 | 0.000483 | 0.002969 | 0.001343 | PPARG/IL6/PTGER3/GSTP1 | 4 |
| BP | GO:0042108 | | positive regulation of cytokine biosynthetic process | 3/43 | 67/18670 | 0.000492 | 0.003012 | 0.001362 | RELA/IL6/HSPB1 | 3 |
| BP | GO:0048662 | | negative regulation of smooth muscle cell proliferation | 3/43 | 67/18670 | 0.000492 | 0.003012 | 0.001362 | PPARG/GSTP1/IGFBP3 | 3 |
| BP | GO:0007519 | | skeletal muscle tissue development | 4/43 | 160/18670 | 0.000494 | 0.003019 | 0.001365 | BCL2/FOS/RB1/CAV1 | 4 |
| BP | GO:0019915 | | lipid storage | 3/43 | 68/18670 | 0.000514 | 0.003124 | 0.001413 | PPARG/IL6/CAV1 | 3 |
| BP | GO:0033627 | | cell adhesion mediated by integrin | 3/43 | 68/18670 | 0.000514 | 0.003124 | 0.001413 | PLAU/ICAM1/CYP1B1 | 3 |
| BP | GO:2001028 | | positive regulation of endothelial cell chemotaxis | 2/43 | 15/18670 | 0.000534 | 0.003238 | 0.001465 | VEGFA/HSPB1 | 2 |
| BP | GO:0051146 | | striated muscle cell differentiation | 5/43 | 293/18670 | 0.000543 | 0.003282 | 0.001484 | VEGFA/BCL2/RB1/CASP3/CCNB1 | 5 |
| BP | GO:0072001 | | renal system development | 5/43 | 293/18670 | 0.000543 | 0.003282 | 0.001484 | AKR1B1/VEGFA/BCL2/CASP9/MYC | 5 |
| BP | GO:0007093 | | mitotic cell cycle checkpoint | 4/43 | 165/18670 | 0.000555 | 0.003336 | 0.001509 | CCND1/RB1/CCNB1/CHEK2 | 4 |
| BP | GO:0071695 | | anatomical structure maturation | 4/43 | 165/18670 | 0.000555 | 0.003336 | 0.001509 | BCL2/RB1/HIF1A/CCNB1 | 4 |
| BP | GO:0097696 | | STAT cascade | 4/43 | 166/18670 | 0.000568 | 0.003405 | 0.00154 | AKR1B1/IL6/CAV1/CYP1B1 | 4 |
| BP | GO:0035051 | | cardiocyte differentiation | 4/43 | 167/18670 | 0.000581 | 0.003475 | 0.001571 | EGFR/VEGFA/VCAM1/CCNB1 | 4 |
| BP | GO:0070372 | | regulation of ERK1 and ERK2 cascade | 5/43 | 300/18670 | 0.000605 | 0.003557 | 0.001609 | EGFR/PRKCA/ERBB2/ICAM1/GSTP1 | 5 |
| BP | GO:0030307 | | positive regulation of cell growth | 4/43 | 169/18670 | 0.000607 | 0.003557 | 0.001609 | EGFR/VEGFA/BCL2/ERBB2 | 4 |
| BP | GO:0060538 | | skeletal muscle organ development | 4/43 | 169/18670 | 0.000607 | 0.003557 | 0.001609 | BCL2/FOS/RB1/CAV1 | 4 |
| BP | GO:0006305 | | DNA alkylation | 3/43 | 72/18670 | 0.000608 | 0.003557 | 0.001609 | FOS/MYC/PARP1 | 3 |
| BP | GO:0006306 | | DNA methylation | 3/43 | 72/18670 | 0.000608 | 0.003557 | 0.001609 | FOS/MYC/PARP1 | 3 |
| BP | GO:0038034 | | signal transduction in absence of ligand | 3/43 | 72/18670 | 0.000608 | 0.003557 | 0.001609 | BCL2/CASP9/CASP3 | 3 |
| BP | GO:0097192 | | extrinsic apoptotic signaling pathway in absence of ligand | 3/43 | 72/18670 | 0.000608 | 0.003557 | 0.001609 | BCL2/CASP9/CASP3 | 3 |
| BP | GO:1903747 | | regulation of establishment of protein localization to mitochondrion | 3/43 | 72/18670 | 0.000608 | 0.003557 | 0.001609 | BCL2/TP63/CASP8 | 3 |
| BP | GO:0030397 | | membrane disassembly | 2/43 | 16/18670 | 0.000609 | 0.003557 | 0.001609 | PRKCA/CCNB1 | 2 |
| BP | GO:0051081 | | nuclear envelope disassembly | 2/43 | 16/18670 | 0.000609 | 0.003557 | 0.001609 | PRKCA/CCNB1 | 2 |
| BP | GO:1904294 | | positive regulation of ERAD pathway | 2/43 | 16/18670 | 0.000609 | 0.003557 | 0.001609 | CAV1/NFE2L2 | 2 |
| BP | GO:0002683 | | negative regulation of immune system process | 6/43 | 463/18670 | 0.000631 | 0.003662 | 0.001656 | PPARG/CASP3/ERBB2/MYC/NFE2L2/COL3A1 | 6 |
| BP | GO:0043627 | | response to estrogen | 3/43 | 73/18670 | 0.000633 | 0.003662 | 0.001656 | PPARG/CCND1/CAV1 | 3 |
| BP | GO:0045685 | | regulation of glial cell differentiation | 3/43 | 73/18670 | 0.000633 | 0.003662 | 0.001656 | PPARG/RELA/IL6 | 3 |
| BP | GO:1903524 | | positive regulation of blood circulation | 3/43 | 73/18670 | 0.000633 | 0.003662 | 0.001656 | EGFR/CAV1/ICAM1 | 3 |
| BP | GO:0001819 | | positive regulation of cytokine production | 6/43 | 464/18670 | 0.000638 | 0.003683 | 0.001666 | RELA/IL6/CASP8/HIF1A/HSPB1/CYP1B1 | 6 |
| BP | GO:0060326 | | cell chemotaxis | 5/43 | 304/18670 | 0.000642 | 0.003701 | 0.001674 | VEGFA/IL6/VCAM1/HSPB1/GSTP1 | 5 |
| BP | GO:0021543 | | pallium development | 4/43 | 172/18670 | 0.000649 | 0.00373 | 0.001687 | EGFR/CASP3/HIF1A/COL3A1 | 4 |
| BP | GO:0014015 | | positive regulation of gliogenesis | 3/43 | 74/18670 | 0.000658 | 0.003769 | 0.001705 | PPARG/RELA/IL6 | 3 |
| BP | GO:0072078 | | nephron tubule morphogenesis | 3/43 | 74/18670 | 0.000658 | 0.003769 | 0.001705 | VEGFA/BCL2/MYC | 3 |
| BP | GO:0003018 | | vascular process in circulatory system | 4/43 | 173/18670 | 0.000663 | 0.003787 | 0.001713 | EGFR/VEGFA/CAV1/ICAM1 | 4 |
| BP | GO:0010224 | | response to UV-B | 2/43 | 17/18670 | 0.000689 | 0.003888 | 0.001759 | RELA/BCL2 | 2 |
| BP | GO:0017085 | | response to insecticide | 2/43 | 17/18670 | 0.000689 | 0.003888 | 0.001759 | CYP1A1/CCNB1 | 2 |
| BP | GO:0032966 | | negative regulation of collagen biosynthetic process | 2/43 | 17/18670 | 0.000689 | 0.003888 | 0.001759 | PPARG/IL6 | 2 |
| BP | GO:0061298 | | retina vasculature development in camera-type eye | 2/43 | 17/18670 | 0.000689 | 0.003888 | 0.001759 | HIF1A/CYP1B1 | 2 |
| BP | GO:1904996 | | positive regulation of leukocyte adhesion to vascular endothelial cell | 2/43 | 17/18670 | 0.000689 | 0.003888 | 0.001759 | RELA/ICAM1 | 2 |
| BP | GO:2000811 | | negative regulation of anoikis | 2/43 | 17/18670 | 0.000689 | 0.003888 | 0.001759 | BCL2/CAV1 | 2 |
| BP | GO:0042310 | | vasoconstriction | 3/43 | 76/18670 | 0.000712 | 0.003988 | 0.001804 | EGFR/CAV1/ICAM1 | 3 |
| BP | GO:0046902 | | regulation of mitochondrial membrane permeability | 3/43 | 76/18670 | 0.000712 | 0.003988 | 0.001804 | BCL2/TP63/CASP8 | 3 |
| BP | GO:0072088 | | nephron epithelium morphogenesis | 3/43 | 76/18670 | 0.000712 | 0.003988 | 0.001804 | VEGFA/BCL2/MYC | 3 |
| BP | GO:0046879 | | hormone secretion | 5/43 | 312/18670 | 0.000722 | 0.004038 | 0.001826 | EGFR/IL6/RAF1/PRKCA/HIF1A | 5 |
| BP | GO:0010950 | | positive regulation of endopeptidase activity | 4/43 | 178/18670 | 0.000737 | 0.004116 | 0.001861 | PPARG/CASP9/CASP8/MYC | 4 |
| BP | GO:0061418 | | regulation of transcription from RNA polymerase II promoter in response to hypoxia | 3/43 | 77/18670 | 0.000739 | 0.004117 | 0.001862 | VEGFA/HIF1A/NFE2L2 | 3 |
| BP | GO:0050863 | | regulation of T cell activation | 5/43 | 314/18670 | 0.000743 | 0.00413 | 0.001868 | IL6/CASP3/ERBB2/CAV1/VCAM1 | 5 |
| BP | GO:0051099 | | positive regulation of binding | 4/43 | 179/18670 | 0.000753 | 0.004146 | 0.001875 | PPARG/RB1/CAV1/PARP1 | 4 |
| BP | GO:0071347 | | cellular response to interleukin-1 | 4/43 | 179/18670 | 0.000753 | 0.004146 | 0.001875 | RELA/IL6/HIF1A/ICAM1 | 4 |
| BP | GO:1903034 | | regulation of response to wounding | 4/43 | 179/18670 | 0.000753 | 0.004146 | 0.001875 | PLAU/PRKCA/CAV1/NFE2L2 | 4 |
| BP | GO:2001235 | | positive regulation of apoptotic signaling pathway | 4/43 | 179/18670 | 0.000753 | 0.004146 | 0.001875 | BCL2/TP63/CASP8/CAV1 | 4 |
| BP | GO:0031098 | | stress-activated protein kinase signaling cascade | 5/43 | 315/18670 | 0.000754 | 0.004146 | 0.001875 | AKR1B1/EGFR/VEGFA/MYC/GSTP1 | 5 |
| BP | GO:0061333 | | renal tubule morphogenesis | 3/43 | 78/18670 | 0.000768 | 0.004187 | 0.001894 | VEGFA/BCL2/MYC | 3 |
| BP | GO:0070664 | | negative regulation of leukocyte proliferation | 3/43 | 78/18670 | 0.000768 | 0.004187 | 0.001894 | CASP3/ERBB2/GSTP1 | 3 |
| BP | GO:0072028 | | nephron morphogenesis | 3/43 | 78/18670 | 0.000768 | 0.004187 | 0.001894 | VEGFA/BCL2/MYC | 3 |
| BP | GO:0072332 | | intrinsic apoptotic signaling pathway by p53 class mediator | 3/43 | 78/18670 | 0.000768 | 0.004187 | 0.001894 | BCL2/TP63/CHEK2 | 3 |
| BP | GO:0010713 | | negative regulation of collagen metabolic process | 2/43 | 18/18670 | 0.000774 | 0.004198 | 0.001899 | PPARG/IL6 | 2 |
| BP | GO:0045780 | | positive regulation of bone resorption | 2/43 | 18/18670 | 0.000774 | 0.004198 | 0.001899 | EGFR/PRKCA | 2 |
| BP | GO:0046852 | | positive regulation of bone remodeling | 2/43 | 18/18670 | 0.000774 | 0.004198 | 0.001899 | EGFR/PRKCA | 2 |
| BP | GO:0010821 | | regulation of mitochondrion organization | 4/43 | 182/18670 | 0.000801 | 0.004326 | 0.001957 | BCL2/TP63/CASP8/HIF1A | 4 |
| BP | GO:0061138 | | morphogenesis of a branching epithelium | 4/43 | 182/18670 | 0.000801 | 0.004326 | 0.001957 | VEGFA/BCL2/TP63/MYC | 4 |
| BP | GO:0009914 | | hormone transport | 5/43 | 322/18670 | 0.000832 | 0.004461 | 0.002017 | EGFR/IL6/RAF1/PRKCA/HIF1A | 5 |
| BP | GO:0060562 | | epithelial tube morphogenesis | 5/43 | 322/18670 | 0.000832 | 0.004461 | 0.002017 | VEGFA/BCL2/CASP3/HIF1A/MYC | 5 |
| BP | GO:0042098 | | T cell proliferation | 4/43 | 184/18670 | 0.000835 | 0.004461 | 0.002017 | IL6/CASP3/ERBB2/VCAM1 | 4 |
| BP | GO:0043401 | | steroid hormone mediated signaling pathway | 4/43 | 184/18670 | 0.000835 | 0.004461 | 0.002017 | PPARG/RB1/TP63/PARP1 | 4 |
| BP | GO:0050864 | | regulation of B cell activation | 4/43 | 184/18670 | 0.000835 | 0.004461 | 0.002017 | BCL2/IL6/CASP3/AHR | 4 |
| BP | GO:0002076 | | osteoblast development | 2/43 | 19/18670 | 0.000864 | 0.004555 | 0.00206 | ACHE/RUNX2 | 2 |
| BP | GO:0002363 | | alpha-beta T cell lineage commitment | 2/43 | 19/18670 | 0.000864 | 0.004555 | 0.00206 | BCL2/IL6 | 2 |
| BP | GO:0010888 | | negative regulation of lipid storage | 2/43 | 19/18670 | 0.000864 | 0.004555 | 0.00206 | PPARG/IL6 | 2 |
| BP | GO:0030949 | | positive regulation of vascular endothelial growth factor receptor signaling pathway | 2/43 | 19/18670 | 0.000864 | 0.004555 | 0.00206 | VEGFA/HIF1A | 2 |
| BP | GO:0032930 | | positive regulation of superoxide anion generation | 2/43 | 19/18670 | 0.000864 | 0.004555 | 0.00206 | EGFR/GSTP1 | 2 |
| BP | GO:0033189 | | response to vitamin A | 2/43 | 19/18670 | 0.000864 | 0.004555 | 0.00206 | PPARG/CYP1A1 | 2 |
| BP | GO:0034138 | | toll-like receptor 3 signaling pathway | 2/43 | 19/18670 | 0.000864 | 0.004555 | 0.00206 | CASP8/CAV1 | 2 |
| BP | GO:0009615 | | response to virus | 5/43 | 326/18670 | 0.000879 | 0.004625 | 0.002092 | RELA/BCL2/IL6/CYP1A1/HSPB1 | 5 |
| BP | GO:0032642 | | regulation of chemokine production | 3/43 | 82/18670 | 0.000888 | 0.004652 | 0.002104 | IL6/HIF1A/GSTP1 | 3 |
| BP | GO:0034644 | | cellular response to UV | 3/43 | 82/18670 | 0.000888 | 0.004652 | 0.002104 | CASP9/MYC/PARP1 | 3 |
| BP | GO:0006367 | | transcription initiation from RNA polymerase II promoter | 4/43 | 188/18670 | 0.000904 | 0.004728 | 0.002138 | PPARG/CCND1/CCNB1/RUNX2 | 4 |
| BP | GO:0006970 | | response to osmotic stress | 3/43 | 83/18670 | 0.00092 | 0.004799 | 0.002171 | AKR1B1/EGFR/CASP3 | 3 |
| BP | GO:0043154 | | negative regulation of cysteine-type endopeptidase activity involved in apoptotic process | 3/43 | 84/18670 | 0.000952 | 0.004936 | 0.002233 | VEGFA/RAF1/BIRC5 | 3 |
| BP | GO:0097756 | | negative regulation of blood vessel diameter | 3/43 | 84/18670 | 0.000952 | 0.004936 | 0.002233 | EGFR/CAV1/ICAM1 | 3 |
| BP | GO:0090150 | | establishment of protein localization to membrane | 5/43 | 332/18670 | 0.000954 | 0.004936 | 0.002233 | EGFR/BCL2/TP63/CASP8/ERBB2 | 5 |
| BP | GO:0019373 | | epoxygenase P450 pathway | 2/43 | 20/18670 | 0.000959 | 0.004936 | 0.002233 | CYP1A1/CYP1B1 | 2 |
| BP | GO:0030220 | | platelet formation | 2/43 | 20/18670 | 0.000959 | 0.004936 | 0.002233 | CASP9/CASP3 | 2 |
| BP | GO:0043369 | | CD4-positive or CD8-positive, alpha-beta T cell lineage commitment | 2/43 | 20/18670 | 0.000959 | 0.004936 | 0.002233 | BCL2/IL6 | 2 |
| BP | GO:0071478 | | cellular response to radiation | 4/43 | 191/18670 | 0.000959 | 0.004936 | 0.002233 | CASP9/MYC/PARP1/CHEK2 | 4 |
| BP | GO:0006919 | | activation of cysteine-type endopeptidase activity involved in apoptotic process | 3/43 | 86/18670 | 0.001019 | 0.005217 | 0.00236 | PPARG/CASP9/CASP8 | 3 |
| BP | GO:0042446 | | hormone biosynthetic process | 3/43 | 86/18670 | 0.001019 | 0.005217 | 0.00236 | AKR1B1/HIF1A/CYP3A4 | 3 |
| BP | GO:0090559 | | regulation of membrane permeability | 3/43 | 86/18670 | 0.001019 | 0.005217 | 0.00236 | BCL2/TP63/CASP8 | 3 |
| BP | GO:0001892 | | embryonic placenta development | 3/43 | 87/18670 | 0.001054 | 0.005323 | 0.002408 | EGFR/CASP8/HIF1A | 3 |
| BP | GO:0001763 | | morphogenesis of a branching structure | 4/43 | 196/18670 | 0.001055 | 0.005323 | 0.002408 | VEGFA/BCL2/TP63/MYC | 4 |
| BP | GO:0032332 | | positive regulation of chondrocyte differentiation | 2/43 | 21/18670 | 0.001058 | 0.005323 | 0.002408 | RELA/RUNX2 | 2 |
| BP | GO:0032682 | | negative regulation of chemokine production | 2/43 | 21/18670 | 0.001058 | 0.005323 | 0.002408 | IL6/GSTP1 | 2 |
| BP | GO:0035162 | | embryonic hemopoiesis | 2/43 | 21/18670 | 0.001058 | 0.005323 | 0.002408 | VEGFA/HIF1A | 2 |
| BP | GO:0036344 | | platelet morphogenesis | 2/43 | 21/18670 | 0.001058 | 0.005323 | 0.002408 | CASP9/CASP3 | 2 |
| BP | GO:0051412 | | response to corticosterone | 2/43 | 21/18670 | 0.001058 | 0.005323 | 0.002408 | CCND1/FOS | 2 |
| BP | GO:0051900 | | regulation of mitochondrial depolarization | 2/43 | 21/18670 | 0.001058 | 0.005323 | 0.002408 | BCL2/PARP1 | 2 |
| BP | GO:1903055 | | positive regulation of extracellular matrix organization | 2/43 | 21/18670 | 0.001058 | 0.005323 | 0.002408 | RB1/IL6 | 2 |
| BP | GO:0009749 | | response to glucose | 4/43 | 197/18670 | 0.001076 | 0.0054 | 0.002442 | CASP3/RAF1/HIF1A/ICAM1 | 4 |
| BP | GO:0033044 | | regulation of chromosome organization | 5/43 | 342/18670 | 0.001089 | 0.00543 | 0.002456 | VEGFA/RB1/MYC/CCNB1/PARP1 | 5 |
| BP | GO:0034103 | | regulation of tissue remodeling | 3/43 | 88/18670 | 0.00109 | 0.00543 | 0.002456 | EGFR/IL6/PRKCA | 3 |
| BP | GO:0097194 | | execution phase of apoptosis | 3/43 | 88/18670 | 0.00109 | 0.00543 | 0.002456 | IL6/CASP3/CASP8 | 3 |
| BP | GO:1904035 | | regulation of epithelial cell apoptotic process | 3/43 | 88/18670 | 0.00109 | 0.00543 | 0.002456 | IL6/ICAM1/NFE2L2 | 3 |
| BP | GO:0007179 | | transforming growth factor beta receptor signaling pathway | 4/43 | 199/18670 | 0.001117 | 0.005553 | 0.002511 | FOS/CAV1/PARP1/COL3A1 | 4 |
| BP | GO:0032602 | | chemokine production | 3/43 | 89/18670 | 0.001126 | 0.005579 | 0.002523 | IL6/HIF1A/GSTP1 | 3 |
| BP | GO:0046427 | | positive regulation of JAK-STAT cascade | 3/43 | 89/18670 | 0.001126 | 0.005579 | 0.002523 | AKR1B1/IL6/CYP1B1 | 3 |
| BP | GO:0002052 | | positive regulation of neuroblast proliferation | 2/43 | 22/18670 | 0.001162 | 0.005656 | 0.002558 | VEGFA/HIF1A | 2 |
| BP | GO:0010869 | | regulation of receptor biosynthetic process | 2/43 | 22/18670 | 0.001162 | 0.005656 | 0.002558 | PPARG/HIF1A | 2 |
| BP | GO:0032928 | | regulation of superoxide anion generation | 2/43 | 22/18670 | 0.001162 | 0.005656 | 0.002558 | EGFR/GSTP1 | 2 |
| BP | GO:0042359 | | vitamin D metabolic process | 2/43 | 22/18670 | 0.001162 | 0.005656 | 0.002558 | CYP3A4/CYP1A1 | 2 |
| BP | GO:1901685 | | glutathione derivative metabolic process | 2/43 | 22/18670 | 0.001162 | 0.005656 | 0.002558 | GSTP1/GSTM1 | 2 |
| BP | GO:1901687 | | glutathione derivative biosynthetic process | 2/43 | 22/18670 | 0.001162 | 0.005656 | 0.002558 | GSTP1/GSTM1 | 2 |
| BP | GO:0001656 | | metanephros development | 3/43 | 90/18670 | 0.001163 | 0.005656 | 0.002558 | AKR1B1/BCL2/MYC | 3 |
| BP | GO:0032651 | | regulation of interleukin-1 beta production | 3/43 | 90/18670 | 0.001163 | 0.005656 | 0.002558 | CASP8/HSPB1/GSTP1 | 3 |
| BP | GO:0045778 | | positive regulation of ossification | 3/43 | 90/18670 | 0.001163 | 0.005656 | 0.002558 | IL6/TP63/RUNX2 | 3 |
| BP | GO:0046849 | | bone remodeling | 3/43 | 90/18670 | 0.001163 | 0.005656 | 0.002558 | EGFR/IL6/PRKCA | 3 |
| BP | GO:0009746 | | response to hexose | 4/43 | 202/18670 | 0.00118 | 0.005729 | 0.002591 | CASP3/RAF1/HIF1A/ICAM1 | 4 |
| BP | GO:0007178 | | transmembrane receptor protein serine/threonine kinase signaling pathway | 5/43 | 349/18670 | 0.001192 | 0.005775 | 0.002612 | FOS/CAV1/PARP1/COL3A1/RUNX2 | 5 |
| BP | GO:0051899 | | membrane depolarization | 3/43 | 91/18670 | 0.001201 | 0.005797 | 0.002622 | BCL2/CAV1/PARP1 | 3 |
| BP | GO:0060333 | | interferon-gamma-mediated signaling pathway | 3/43 | 91/18670 | 0.001201 | 0.005797 | 0.002622 | PPARG/ICAM1/VCAM1 | 3 |
| BP | GO:1904894 | | positive regulation of STAT cascade | 3/43 | 92/18670 | 0.001239 | 0.005961 | 0.002696 | AKR1B1/IL6/CYP1B1 | 3 |
| BP | GO:2000117 | | negative regulation of cysteine-type endopeptidase activity | 3/43 | 92/18670 | 0.001239 | 0.005961 | 0.002696 | VEGFA/RAF1/BIRC5 | 3 |
| BP | GO:0030098 | | lymphocyte differentiation | 5/43 | 353/18670 | 0.001253 | 0.00602 | 0.002722 | BCL2/IL6/ERBB2/VCAM1/RUNX2 | 5 |
| BP | GO:0001505 | | regulation of neurotransmitter levels | 5/43 | 354/18670 | 0.001269 | 0.006061 | 0.002741 | ACHE/CAV1/ICAM1/CYP1B1/NQO1 | 5 |
| BP | GO:0031589 | | cell-substrate adhesion | 5/43 | 354/18670 | 0.001269 | 0.006061 | 0.002741 | VEGFA/BCL2/PLAU/VCAM1/COL3A1 | 5 |
| BP | GO:0051882 | | mitochondrial depolarization | 2/43 | 23/18670 | 0.001271 | 0.006061 | 0.002741 | BCL2/PARP1 | 2 |
| BP | GO:0071157 | | negative regulation of cell cycle arrest | 2/43 | 23/18670 | 0.001271 | 0.006061 | 0.002741 | CCND1/CHEK2 | 2 |
| BP | GO:0072080 | | nephron tubule development | 3/43 | 93/18670 | 0.001278 | 0.006084 | 0.002751 | VEGFA/BCL2/MYC | 3 |
| BP | GO:0002064 | | epithelial cell development | 4/43 | 207/18670 | 0.001291 | 0.006135 | 0.002775 | VEGFA/TP63/HIF1A/ICAM1 | 4 |
| BP | GO:0044728 | | DNA methylation or demethylation | 3/43 | 94/18670 | 0.001318 | 0.006241 | 0.002822 | FOS/MYC/PARP1 | 3 |
| BP | GO:0060993 | | kidney morphogenesis | 3/43 | 94/18670 | 0.001318 | 0.006241 | 0.002822 | VEGFA/BCL2/MYC | 3 |
| BP | GO:0051701 | | interaction with host | 4/43 | 209/18670 | 0.001338 | 0.006323 | 0.002859 | EGFR/CASP8/CAV1/ICAM1 | 4 |
| BP | GO:0042100 | | B cell proliferation | 3/43 | 95/18670 | 0.001359 | 0.0064 | 0.002894 | BCL2/CASP3/AHR | 3 |
| BP | GO:0061326 | | renal tubule development | 3/43 | 95/18670 | 0.001359 | 0.0064 | 0.002894 | VEGFA/BCL2/MYC | 3 |
| BP | GO:0010948 | | negative regulation of cell cycle process | 5/43 | 361/18670 | 0.001384 | 0.006486 | 0.002933 | CCND1/BCL2/RB1/CCNB1/CHEK2 | 5 |
| BP | GO:0044346 | | fibroblast apoptotic process | 2/43 | 24/18670 | 0.001385 | 0.006486 | 0.002933 | TP63/MYC | 2 |
| BP | GO:2001026 | | regulation of endothelial cell chemotaxis | 2/43 | 24/18670 | 0.001385 | 0.006486 | 0.002933 | VEGFA/HSPB1 | 2 |
| BP | GO:0048010 | | vascular endothelial growth factor receptor signaling pathway | 3/43 | 96/18670 | 0.001401 | 0.006537 | 0.002957 | VEGFA/HIF1A/HSPB1 | 3 |
| BP | GO:0097327 | | response to antineoplastic agent | 3/43 | 96/18670 | 0.001401 | 0.006537 | 0.002957 | EGFR/CASP9/ICAM1 | 3 |
| BP | GO:0001657 | | ureteric bud development | 3/43 | 97/18670 | 0.001443 | 0.006665 | 0.003014 | VEGFA/BCL2/MYC | 3 |
| BP | GO:0044773 | | mitotic DNA damage checkpoint | 3/43 | 97/18670 | 0.001443 | 0.006665 | 0.003014 | CCND1/CCNB1/CHEK2 | 3 |
| BP | GO:0048709 | | oligodendrocyte differentiation | 3/43 | 97/18670 | 0.001443 | 0.006665 | 0.003014 | PPARG/ERBB2/GSTP1 | 3 |
| BP | GO:0051591 | | response to cAMP | 3/43 | 97/18670 | 0.001443 | 0.006665 | 0.003014 | RELA/FOS/AHR | 3 |
| BP | GO:0120162 | | positive regulation of cold-induced thermogenesis | 3/43 | 97/18670 | 0.001443 | 0.006665 | 0.003014 | ACHE/VEGFA/CAV1 | 3 |
| BP | GO:2001022 | | positive regulation of response to DNA damage stimulus | 3/43 | 97/18670 | 0.001443 | 0.006665 | 0.003014 | EGFR/MYC/PARP1 | 3 |
| BP | GO:0072163 | | mesonephric epithelium development | 3/43 | 98/18670 | 0.001486 | 0.006841 | 0.003094 | VEGFA/BCL2/MYC | 3 |
| BP | GO:0072164 | | mesonephric tubule development | 3/43 | 98/18670 | 0.001486 | 0.006841 | 0.003094 | VEGFA/BCL2/MYC | 3 |
| BP | GO:0002053 | | positive regulation of mesenchymal cell proliferation | 2/43 | 25/18670 | 0.001503 | 0.00687 | 0.003107 | VEGFA/MYC | 2 |
| BP | GO:0019430 | | removal of superoxide radicals | 2/43 | 25/18670 | 0.001503 | 0.00687 | 0.003107 | NFE2L2/NQO1 | 2 |
| BP | GO:0031069 | | hair follicle morphogenesis | 2/43 | 25/18670 | 0.001503 | 0.00687 | 0.003107 | BCL2/TP63 | 2 |
| BP | GO:2000679 | | positive regulation of transcription regulatory region DNA binding | 2/43 | 25/18670 | 0.001503 | 0.00687 | 0.003107 | RB1/PARP1 | 2 |
| BP | GO:0000075 | | cell cycle checkpoint | 4/43 | 216/18670 | 0.00151 | 0.006879 | 0.003111 | CCND1/RB1/CCNB1/CHEK2 | 4 |
| BP | GO:0032869 | | cellular response to insulin stimulus | 4/43 | 216/18670 | 0.00151 | 0.006879 | 0.003111 | PPARG/RELA/GSTP1/PARP1 | 4 |
| BP | GO:0050920 | | regulation of chemotaxis | 4/43 | 217/18670 | 0.001536 | 0.006984 | 0.003159 | VEGFA/IL6/HSPB1/GSTP1 | 4 |
| BP | GO:0001701 | | in utero embryonic development | 5/43 | 373/18670 | 0.001599 | 0.007244 | 0.003276 | EGFR/VEGFA/CASP8/HIF1A/CCNB1 | 5 |
| BP | GO:0051098 | | regulation of binding | 5/43 | 373/18670 | 0.001599 | 0.007244 | 0.003276 | PPARG/BCL2/RB1/CAV1/PARP1 | 5 |
| BP | GO:0032611 | | interleukin-1 beta production | 3/43 | 101/18670 | 0.001621 | 0.007293 | 0.003298 | CASP8/HSPB1/GSTP1 | 3 |
| BP | GO:0048661 | | positive regulation of smooth muscle cell proliferation | 3/43 | 101/18670 | 0.001621 | 0.007293 | 0.003298 | AKR1B1/EGFR/IL6 | 3 |
| BP | GO:0002360 | | T cell lineage commitment | 2/43 | 26/18670 | 0.001626 | 0.007293 | 0.003298 | BCL2/IL6 | 2 |
| BP | GO:0006309 | | apoptotic DNA fragmentation | 2/43 | 26/18670 | 0.001626 | 0.007293 | 0.003298 | IL6/CASP3 | 2 |
| BP | GO:0060544 | | regulation of necroptotic process | 2/43 | 26/18670 | 0.001626 | 0.007293 | 0.003298 | CASP8/CAV1 | 2 |
| BP | GO:1903203 | | regulation of oxidative stress-induced neuron death | 2/43 | 26/18670 | 0.001626 | 0.007293 | 0.003298 | HIF1A/PARP1 | 2 |
| BP | GO:0001823 | | mesonephros development | 3/43 | 102/18670 | 0.001667 | 0.007464 | 0.003376 | VEGFA/BCL2/MYC | 3 |
| BP | GO:0044706 | | multi-multicellular organism process | 4/43 | 222/18670 | 0.00167 | 0.007464 | 0.003376 | AKR1B1/BCL2/FOS/CYP1A1 | 4 |
| BP | GO:0032652 | | regulation of interleukin-1 production | 3/43 | 103/18670 | 0.001714 | 0.007652 | 0.00346 | CASP8/HSPB1/GSTP1 | 3 |
| BP | GO:0010971 | | positive regulation of G2/M transition of mitotic cell cycle | 2/43 | 27/18670 | 0.001753 | 0.007752 | 0.003506 | CCND1/CCNB1 | 2 |
| BP | GO:0036475 | | neuron death in response to oxidative stress | 2/43 | 27/18670 | 0.001753 | 0.007752 | 0.003506 | HIF1A/PARP1 | 2 |
| BP | GO:0048143 | | astrocyte activation | 2/43 | 27/18670 | 0.001753 | 0.007752 | 0.003506 | EGFR/IL6 | 2 |
| BP | GO:0071450 | | cellular response to oxygen radical | 2/43 | 27/18670 | 0.001753 | 0.007752 | 0.003506 | NFE2L2/NQO1 | 2 |
| BP | GO:0071451 | | cellular response to superoxide | 2/43 | 27/18670 | 0.001753 | 0.007752 | 0.003506 | NFE2L2/NQO1 | 2 |
| BP | GO:0042176 | | regulation of protein catabolic process | 5/43 | 381/18670 | 0.001755 | 0.007752 | 0.003506 | RELA/EGFR/CAV1/NFE2L2/CHEK2 | 5 |
| BP | GO:0042692 | | muscle cell differentiation | 5/43 | 385/18670 | 0.001837 | 0.008101 | 0.003664 | VEGFA/BCL2/RB1/CASP3/CCNB1 | 5 |
| BP | GO:0051054 | | positive regulation of DNA metabolic process | 4/43 | 228/18670 | 0.00184 | 0.008105 | 0.003666 | EGFR/IL6/MYC/PARP1 | 4 |
| BP | GO:0007052 | | mitotic spindle organization | 3/43 | 106/18670 | 0.001861 | 0.008169 | 0.003695 | BIRC5/CCNB1/CHEK2 | 3 |
| BP | GO:0044774 | | mitotic DNA integrity checkpoint | 3/43 | 106/18670 | 0.001861 | 0.008169 | 0.003695 | CCND1/CCNB1/CHEK2 | 3 |
| BP | GO:0051984 | | positive regulation of chromosome segregation | 2/43 | 28/18670 | 0.001886 | 0.008248 | 0.00373 | RB1/CCNB1 | 2 |
| BP | GO:1904994 | | regulation of leukocyte adhesion to vascular endothelial cell | 2/43 | 28/18670 | 0.001886 | 0.008248 | 0.00373 | RELA/ICAM1 | 2 |
| BP | GO:0051341 | | regulation of oxidoreductase activity | 3/43 | 107/18670 | 0.001912 | 0.00835 | 0.003776 | EGFR/HIF1A/CAV1 | 3 |
| BP | GO:0071156 | | regulation of cell cycle arrest | 3/43 | 108/18670 | 0.001963 | 0.008561 | 0.003872 | CCND1/CCNB1/CHEK2 | 3 |
| BP | GO:0072009 | | nephron epithelium development | 3/43 | 109/18670 | 0.002016 | 0.008703 | 0.003936 | VEGFA/BCL2/MYC | 3 |
| BP | GO:0000303 | | response to superoxide | 2/43 | 29/18670 | 0.002022 | 0.008703 | 0.003936 | NFE2L2/NQO1 | 2 |
| BP | GO:0007263 | | nitric oxide mediated signal transduction | 2/43 | 29/18670 | 0.002022 | 0.008703 | 0.003936 | EGFR/VEGFA | 2 |
| BP | GO:0007271 | | synaptic transmission, cholinergic | 2/43 | 29/18670 | 0.002022 | 0.008703 | 0.003936 | ACHE/NQO1 | 2 |
| BP | GO:0044030 | | regulation of DNA methylation | 2/43 | 29/18670 | 0.002022 | 0.008703 | 0.003936 | MYC/PARP1 | 2 |
| BP | GO:0048730 | | epidermis morphogenesis | 2/43 | 29/18670 | 0.002022 | 0.008703 | 0.003936 | BCL2/TP63 | 2 |
| BP | GO:0071280 | | cellular response to copper ion | 2/43 | 29/18670 | 0.002022 | 0.008703 | 0.003936 | CYP1A1/NFE2L2 | 2 |
| BP | GO:1900027 | | regulation of ruffle assembly | 2/43 | 29/18670 | 0.002022 | 0.008703 | 0.003936 | CAV1/ICAM1 | 2 |
| BP | GO:0009755 | | hormone-mediated signaling pathway | 4/43 | 235/18670 | 0.002055 | 0.008828 | 0.003993 | PPARG/RB1/TP63/PARP1 | 4 |
| BP | GO:0031669 | | cellular response to nutrient levels | 4/43 | 237/18670 | 0.002119 | 0.00906 | 0.004098 | PPARG/BCL2/ICAM1/NFE2L2 | 4 |
| BP | GO:0032872 | | regulation of stress-activated MAPK cascade | 4/43 | 237/18670 | 0.002119 | 0.00906 | 0.004098 | EGFR/VEGFA/MYC/GSTP1 | 4 |
| BP | GO:0043122 | | regulation of I-kappaB kinase/NF-kappaB signaling | 4/43 | 237/18670 | 0.002119 | 0.00906 | 0.004098 | RELA/CASP8/HSPB1/GSTP1 | 4 |
| BP | GO:0031623 | | receptor internalization | 3/43 | 111/18670 | 0.002123 | 0.009063 | 0.004099 | ACHE/VEGFA/CAV1 | 3 |
| BP | GO:0000305 | | response to oxygen radical | 2/43 | 30/18670 | 0.002164 | 0.009162 | 0.004144 | NFE2L2/NQO1 | 2 |
| BP | GO:0042759 | | long-chain fatty acid biosynthetic process | 2/43 | 30/18670 | 0.002164 | 0.009162 | 0.004144 | CYP3A4/CYP1A1 | 2 |
| BP | GO:0097421 | | liver regeneration | 2/43 | 30/18670 | 0.002164 | 0.009162 | 0.004144 | EGFR/CCND1 | 2 |
| BP | GO:1902751 | | positive regulation of cell cycle G2/M phase transition | 2/43 | 30/18670 | 0.002164 | 0.009162 | 0.004144 | CCND1/CCNB1 | 2 |
| BP | GO:1904292 | | regulation of ERAD pathway | 2/43 | 30/18670 | 0.002164 | 0.009162 | 0.004144 | CAV1/NFE2L2 | 2 |
| BP | GO:0001938 | | positive regulation of endothelial cell proliferation | 3/43 | 112/18670 | 0.002178 | 0.009209 | 0.004165 | VEGFA/PRKCA/HIF1A | 3 |
| BP | GO:0070302 | | regulation of stress-activated protein kinase signaling cascade | 4/43 | 239/18670 | 0.002185 | 0.009222 | 0.004171 | EGFR/VEGFA/MYC/GSTP1 | 4 |
| BP | GO:0030217 | | T cell differentiation | 4/43 | 240/18670 | 0.002218 | 0.009348 | 0.004228 | BCL2/IL6/ERBB2/RUNX2 | 4 |
| BP | GO:0034329 | | cell junction assembly | 4/43 | 241/18670 | 0.002252 | 0.00946 | 0.004278 | VEGFA/BCL2/PRKCA/CAV1 | 4 |
| BP | GO:0042593 | | glucose homeostasis | 4/43 | 241/18670 | 0.002252 | 0.00946 | 0.004278 | PPARG/RAF1/HIF1A/ICAM1 | 4 |
| BP | GO:0033500 | | carbohydrate homeostasis | 4/43 | 242/18670 | 0.002286 | 0.009578 | 0.004332 | PPARG/RAF1/HIF1A/ICAM1 | 4 |
| BP | GO:0006690 | | icosanoid metabolic process | 3/43 | 114/18670 | 0.002291 | 0.009578 | 0.004332 | PTGS1/CYP1A1/CYP1B1 | 3 |
| BP | GO:0042035 | | regulation of cytokine biosynthetic process | 3/43 | 114/18670 | 0.002291 | 0.009578 | 0.004332 | RELA/IL6/HSPB1 | 3 |
| BP | GO:0002675 | | positive regulation of acute inflammatory response | 2/43 | 31/18670 | 0.002309 | 0.009581 | 0.004333 | IL6/PTGER3 | 2 |
| BP | GO:0010165 | | response to X-ray | 2/43 | 31/18670 | 0.002309 | 0.009581 | 0.004333 | CCND1/CASP3 | 2 |
| BP | GO:0035767 | | endothelial cell chemotaxis | 2/43 | 31/18670 | 0.002309 | 0.009581 | 0.004333 | VEGFA/HSPB1 | 2 |
| BP | GO:0043457 | | regulation of cellular respiration | 2/43 | 31/18670 | 0.002309 | 0.009581 | 0.004333 | HIF1A/CCNB1 | 2 |
| BP | GO:0045879 | | negative regulation of smoothened signaling pathway | 2/43 | 31/18670 | 0.002309 | 0.009581 | 0.004333 | RB1/RUNX2 | 2 |
| BP | GO:0032612 | | interleukin-1 production | 3/43 | 115/18670 | 0.002348 | 0.009711 | 0.004392 | CASP8/HSPB1/GSTP1 | 3 |
| BP | GO:0032963 | | collagen metabolic process | 3/43 | 115/18670 | 0.002348 | 0.009711 | 0.004392 | PPARG/IL6/HIF1A | 3 |
| BP | GO:0006304 | | DNA modification | 3/43 | 116/18670 | 0.002407 | 0.009907 | 0.00448 | FOS/MYC/PARP1 | 3 |
| BP | GO:0021782 | | glial cell development | 3/43 | 116/18670 | 0.002407 | 0.009907 | 0.00448 | EGFR/IL6/GSTP1 | 3 |
| BP | GO:0021987 | | cerebral cortex development | 3/43 | 116/18670 | 0.002407 | 0.009907 | 0.00448 | EGFR/HIF1A/COL3A1 | 3 |
| BP | GO:0008608 | | attachment of spindle microtubules to kinetochore | 2/43 | 32/18670 | 0.00246 | 0.010078 | 0.004558 | RB1/CCNB1 | 2 |
| BP | GO:0042573 | | retinoic acid metabolic process | 2/43 | 32/18670 | 0.00246 | 0.010078 | 0.004558 | CYP3A4/CYP1A1 | 2 |
| BP | GO:1901976 | | regulation of cell cycle checkpoint | 2/43 | 32/18670 | 0.00246 | 0.010078 | 0.004558 | CCNB1/CHEK2 | 2 |
| BP | GO:0042180 | | cellular ketone metabolic process | 4/43 | 248/18670 | 0.002497 | 0.010217 | 0.004621 | PPARG/AKR1B1/CAV1/NQO1 | 4 |
| BP | GO:0006352 | | DNA-templated transcription, initiation | 4/43 | 249/18670 | 0.002534 | 0.010335 | 0.004674 | PPARG/CCND1/CCNB1/RUNX2 | 4 |
| BP | GO:0071560 | | cellular response to transforming growth factor beta stimulus | 4/43 | 249/18670 | 0.002534 | 0.010335 | 0.004674 | FOS/CAV1/PARP1/COL3A1 | 4 |
| BP | GO:0001952 | | regulation of cell-matrix adhesion | 3/43 | 119/18670 | 0.002588 | 0.010486 | 0.004742 | VEGFA/BCL2/PLAU | 3 |
| BP | GO:0021537 | | telencephalon development | 4/43 | 251/18670 | 0.002608 | 0.010486 | 0.004742 | EGFR/CASP3/HIF1A/COL3A1 | 4 |
| BP | GO:0010464 | | regulation of mesenchymal cell proliferation | 2/43 | 33/18670 | 0.002615 | 0.010486 | 0.004742 | VEGFA/MYC | 2 |
| BP | GO:0033028 | | myeloid cell apoptotic process | 2/43 | 33/18670 | 0.002615 | 0.010486 | 0.004742 | BCL2/IL6 | 2 |
| BP | GO:0035115 | | embryonic forelimb morphogenesis | 2/43 | 33/18670 | 0.002615 | 0.010486 | 0.004742 | TP63/RUNX2 | 2 |
| BP | GO:0048011 | | neurotrophin TRK receptor signaling pathway | 2/43 | 33/18670 | 0.002615 | 0.010486 | 0.004742 | CASP3/RAF1 | 2 |
| BP | GO:0051194 | | positive regulation of cofactor metabolic process | 2/43 | 33/18670 | 0.002615 | 0.010486 | 0.004742 | HIF1A/NFE2L2 | 2 |
| BP | GO:0061036 | | positive regulation of cartilage development | 2/43 | 33/18670 | 0.002615 | 0.010486 | 0.004742 | RELA/RUNX2 | 2 |
| BP | GO:1901890 | | positive regulation of cell junction assembly | 2/43 | 33/18670 | 0.002615 | 0.010486 | 0.004742 | VEGFA/CAV1 | 2 |
| BP | GO:1902692 | | regulation of neuroblast proliferation | 2/43 | 33/18670 | 0.002615 | 0.010486 | 0.004742 | VEGFA/HIF1A | 2 |
| BP | GO:2000352 | | negative regulation of endothelial cell apoptotic process | 2/43 | 33/18670 | 0.002615 | 0.010486 | 0.004742 | ICAM1/NFE2L2 | 2 |
| BP | GO:0051222 | | positive regulation of protein transport | 5/43 | 418/18670 | 0.002626 | 0.010514 | 0.004755 | ACHE/EGFR/IL6/HIF1A/ERBB2 | 5 |
| BP | GO:2000027 | | regulation of animal organ morphogenesis | 4/43 | 253/18670 | 0.002684 | 0.010732 | 0.004854 | VEGFA/BCL2/MYC/RUNX2 | 4 |
| BP | GO:0043618 | | regulation of transcription from RNA polymerase II promoter in response to stress | 3/43 | 121/18670 | 0.002713 | 0.010814 | 0.004891 | VEGFA/HIF1A/NFE2L2 | 3 |
| BP | GO:0046718 | | viral entry into host cell | 3/43 | 121/18670 | 0.002713 | 0.010814 | 0.004891 | EGFR/CAV1/ICAM1 | 3 |
| BP | GO:0071559 | | response to transforming growth factor beta | 4/43 | 255/18670 | 0.002761 | 0.010991 | 0.004971 | FOS/CAV1/PARP1/COL3A1 | 4 |
| BP | GO:0000737 | | DNA catabolic process, endonucleolytic | 2/43 | 34/18670 | 0.002774 | 0.011003 | 0.004976 | IL6/CASP3 | 2 |
| BP | GO:0043368 | | positive T cell selection | 2/43 | 34/18670 | 0.002774 | 0.011003 | 0.004976 | BCL2/IL6 | 2 |
| BP | GO:1903578 | | regulation of ATP metabolic process | 3/43 | 122/18670 | 0.002777 | 0.011003 | 0.004976 | HIF1A/CCNB1/PARP1 | 3 |
| BP | GO:0007611 | | learning or memory | 4/43 | 256/18670 | 0.0028 | 0.01108 | 0.005011 | EGFR/FOS/CASP3/HIF1A | 4 |
| BP | GO:0048863 | | stem cell differentiation | 4/43 | 257/18670 | 0.00284 | 0.01121 | 0.00507 | TP63/HIF1A/NFE2L2/RUNX2 | 4 |
| BP | GO:0042089 | | cytokine biosynthetic process | 3/43 | 123/18670 | 0.002842 | 0.01121 | 0.00507 | RELA/IL6/HSPB1 | 3 |
| BP | GO:0043406 | | positive regulation of MAP kinase activity | 4/43 | 258/18670 | 0.00288 | 0.011344 | 0.00513 | EGFR/VEGFA/RAF1/ERBB2 | 4 |
| BP | GO:0048568 | | embryonic organ development | 5/43 | 428/18670 | 0.002907 | 0.011368 | 0.005141 | EGFR/VEGFA/CASP8/HIF1A/RUNX2 | 5 |
| BP | GO:0008637 | | apoptotic mitochondrial changes | 3/43 | 124/18670 | 0.002907 | 0.011368 | 0.005141 | BCL2/TP63/CASP8 | 3 |
| BP | GO:0014013 | | regulation of gliogenesis | 3/43 | 124/18670 | 0.002907 | 0.011368 | 0.005141 | PPARG/RELA/IL6 | 3 |
| BP | GO:0042107 | | cytokine metabolic process | 3/43 | 124/18670 | 0.002907 | 0.011368 | 0.005141 | RELA/IL6/HSPB1 | 3 |
| BP | GO:0051101 | | regulation of DNA binding | 3/43 | 124/18670 | 0.002907 | 0.011368 | 0.005141 | PPARG/RB1/PARP1 | 3 |
| BP | GO:0030262 | | apoptotic nuclear changes | 2/43 | 35/18670 | 0.002938 | 0.011436 | 0.005172 | IL6/CASP3 | 2 |
| BP | GO:0070423 | | nucleotide-binding oligomerization domain containing signaling pathway | 2/43 | 35/18670 | 0.002938 | 0.011436 | 0.005172 | RELA/CASP8 | 2 |
| BP | GO:0071312 | | cellular response to alkaloid | 2/43 | 35/18670 | 0.002938 | 0.011436 | 0.005172 | CASP3/ICAM1 | 2 |
| BP | GO:0045667 | | regulation of osteoblast differentiation | 3/43 | 126/18670 | 0.003042 | 0.011824 | 0.005347 | IL6/TP63/RUNX2 | 3 |
| BP | GO:0030947 | | regulation of vascular endothelial growth factor receptor signaling pathway | 2/43 | 36/18670 | 0.003106 | 0.012021 | 0.005436 | VEGFA/HIF1A | 2 |
| BP | GO:0035872 | | nucleotide-binding domain, leucine rich repeat containing receptor signaling pathway | 2/43 | 36/18670 | 0.003106 | 0.012021 | 0.005436 | RELA/CASP8 | 2 |
| BP | GO:0042554 | | superoxide anion generation | 2/43 | 36/18670 | 0.003106 | 0.012021 | 0.005436 | EGFR/GSTP1 | 2 |
| BP | GO:0140014 | | mitotic nuclear division | 4/43 | 264/18670 | 0.003128 | 0.012086 | 0.005466 | RB1/BIRC5/CCNB1/CHEK2 | 4 |
| BP | GO:0002687 | | positive regulation of leukocyte migration | 3/43 | 128/18670 | 0.00318 | 0.012271 | 0.00555 | VEGFA/IL6/ICAM1 | 3 |
| BP | GO:0071482 | | cellular response to light stimulus | 3/43 | 129/18670 | 0.00325 | 0.012525 | 0.005664 | CASP9/MYC/PARP1 | 3 |
| BP | GO:0072331 | | signal transduction by p53 class mediator | 4/43 | 267/18670 | 0.003257 | 0.012531 | 0.005667 | BCL2/TP63/CCNB1/CHEK2 | 4 |
| BP | GO:0006921 | | cellular component disassembly involved in execution phase of apoptosis | 2/43 | 37/18670 | 0.003279 | 0.012543 | 0.005673 | IL6/CASP3 | 2 |
| BP | GO:0043029 | | T cell homeostasis | 2/43 | 37/18670 | 0.003279 | 0.012543 | 0.005673 | BCL2/CASP3 | 2 |
| BP | GO:0071276 | | cellular response to cadmium ion | 2/43 | 37/18670 | 0.003279 | 0.012543 | 0.005673 | EGFR/FOS | 2 |
| BP | GO:0071542 | | dopaminergic neuron differentiation | 2/43 | 37/18670 | 0.003279 | 0.012543 | 0.005673 | VEGFA/HIF1A | 2 |
| BP | GO:0050714 | | positive regulation of protein secretion | 4/43 | 268/18670 | 0.003301 | 0.012608 | 0.005702 | ACHE/EGFR/IL6/HIF1A | 4 |
| BP | GO:0050671 | | positive regulation of lymphocyte proliferation | 3/43 | 130/18670 | 0.003322 | 0.012671 | 0.005731 | BCL2/IL6/VCAM1 | 3 |
| BP | GO:0007249 | | I-kappaB kinase/NF-kappaB signaling | 4/43 | 269/18670 | 0.003345 | 0.012741 | 0.005762 | RELA/CASP8/HSPB1/GSTP1 | 4 |
| BP | GO:0032946 | | positive regulation of mononuclear cell proliferation | 3/43 | 131/18670 | 0.003394 | 0.012892 | 0.00583 | BCL2/IL6/VCAM1 | 3 |
| BP | GO:1902850 | | microtubule cytoskeleton organization involved in mitosis | 3/43 | 131/18670 | 0.003394 | 0.012892 | 0.00583 | BIRC5/CCNB1/CHEK2 | 3 |
| BP | GO:0010939 | | regulation of necrotic cell death | 2/43 | 38/18670 | 0.003456 | 0.013051 | 0.005902 | CASP8/CAV1 | 2 |
| BP | GO:0016572 | | histone phosphorylation | 2/43 | 38/18670 | 0.003456 | 0.013051 | 0.005902 | PRKCA/CCNB1 | 2 |
| BP | GO:1904706 | | negative regulation of vascular smooth muscle cell proliferation | 2/43 | 38/18670 | 0.003456 | 0.013051 | 0.005902 | PPARG/GSTP1 | 2 |
| BP | GO:1905898 | | positive regulation of response to endoplasmic reticulum stress | 2/43 | 38/18670 | 0.003456 | 0.013051 | 0.005902 | CAV1/NFE2L2 | 2 |
| BP | GO:0030518 | | intracellular steroid hormone receptor signaling pathway | 3/43 | 132/18670 | 0.003468 | 0.013077 | 0.005914 | RB1/TP63/PARP1 | 3 |
| BP | GO:0042770 | | signal transduction in response to DNA damage | 3/43 | 133/18670 | 0.003542 | 0.01332 | 0.006024 | CASP9/CCNB1/CHEK2 | 3 |
| BP | GO:0071333 | | cellular response to glucose stimulus | 3/43 | 133/18670 | 0.003542 | 0.01332 | 0.006024 | RAF1/HIF1A/ICAM1 | 3 |
| BP | GO:0007006 | | mitochondrial membrane organization | 3/43 | 134/18670 | 0.003618 | 0.013488 | 0.0061 | BCL2/TP63/CASP8 | 3 |
| BP | GO:0030260 | | entry into host cell | 3/43 | 134/18670 | 0.003618 | 0.013488 | 0.0061 | EGFR/CAV1/ICAM1 | 3 |
| BP | GO:0044409 | | entry into host | 3/43 | 134/18670 | 0.003618 | 0.013488 | 0.0061 | EGFR/CAV1/ICAM1 | 3 |
| BP | GO:0046683 | | response to organophosphorus | 3/43 | 134/18670 | 0.003618 | 0.013488 | 0.0061 | RELA/FOS/AHR | 3 |
| BP | GO:0051806 | | entry into cell of other organism involved in symbiotic interaction | 3/43 | 134/18670 | 0.003618 | 0.013488 | 0.0061 | EGFR/CAV1/ICAM1 | 3 |
| BP | GO:0051828 | | entry into other organism involved in symbiotic interaction | 3/43 | 134/18670 | 0.003618 | 0.013488 | 0.0061 | EGFR/CAV1/ICAM1 | 3 |
| BP | GO:0033146 | | regulation of intracellular estrogen receptor signaling pathway | 2/43 | 39/18670 | 0.003637 | 0.013505 | 0.006108 | TP63/PARP1 | 2 |
| BP | GO:0038179 | | neurotrophin signaling pathway | 2/43 | 39/18670 | 0.003637 | 0.013505 | 0.006108 | CASP3/RAF1 | 2 |
| BP | GO:2000648 | | positive regulation of stem cell proliferation | 2/43 | 39/18670 | 0.003637 | 0.013505 | 0.006108 | VEGFA/HIF1A | 2 |
| BP | GO:0045088 | | regulation of innate immune response | 5/43 | 452/18670 | 0.003672 | 0.013613 | 0.006157 | PPARG/RELA/CASP8/RAF1/CAV1 | 5 |
| BP | GO:0050921 | | positive regulation of chemotaxis | 3/43 | 135/18670 | 0.003694 | 0.013657 | 0.006177 | VEGFA/IL6/HSPB1 | 3 |
| BP | GO:0071331 | | cellular response to hexose stimulus | 3/43 | 135/18670 | 0.003694 | 0.013657 | 0.006177 | RAF1/HIF1A/ICAM1 | 3 |
| BP | GO:0060485 | | mesenchyme development | 4/43 | 278/18670 | 0.003762 | 0.013889 | 0.006282 | BCL2/IL6/HIF1A/MYC | 4 |
| BP | GO:0071326 | | cellular response to monosaccharide stimulus | 3/43 | 136/18670 | 0.003771 | 0.013904 | 0.006288 | RAF1/HIF1A/ICAM1 | 3 |
| BP | GO:0071470 | | cellular response to osmotic stress | 2/43 | 40/18670 | 0.003823 | 0.014057 | 0.006358 | AKR1B1/CASP3 | 2 |
| BP | GO:0090184 | | positive regulation of kidney development | 2/43 | 40/18670 | 0.003823 | 0.014057 | 0.006358 | VEGFA/MYC | 2 |
| BP | GO:0072655 | | establishment of protein localization to mitochondrion | 3/43 | 137/18670 | 0.003849 | 0.014134 | 0.006392 | BCL2/TP63/CASP8 | 3 |
| BP | GO:0008584 | | male gonad development | 3/43 | 138/18670 | 0.003929 | 0.014385 | 0.006506 | CCND1/BCL2/ICAM1 | 3 |
| BP | GO:0009308 | | amine metabolic process | 3/43 | 138/18670 | 0.003929 | 0.014385 | 0.006506 | CYP1A1/VCAM1/NQO1 | 3 |
| BP | GO:0046546 | | development of primary male sexual characteristics | 3/43 | 139/18670 | 0.004009 | 0.014555 | 0.006583 | CCND1/BCL2/ICAM1 | 3 |
| BP | GO:0070665 | | positive regulation of leukocyte proliferation | 3/43 | 139/18670 | 0.004009 | 0.014555 | 0.006583 | BCL2/IL6/VCAM1 | 3 |
| BP | GO:0006308 | | DNA catabolic process | 2/43 | 41/18670 | 0.004013 | 0.014555 | 0.006583 | IL6/CASP3 | 2 |
| BP | GO:0035136 | | forelimb morphogenesis | 2/43 | 41/18670 | 0.004013 | 0.014555 | 0.006583 | TP63/RUNX2 | 2 |
| BP | GO:0061028 | | establishment of endothelial barrier | 2/43 | 41/18670 | 0.004013 | 0.014555 | 0.006583 | VEGFA/ICAM1 | 2 |
| BP | GO:0097178 | | ruffle assembly | 2/43 | 41/18670 | 0.004013 | 0.014555 | 0.006583 | CAV1/ICAM1 | 2 |
| BP | GO:1903053 | | regulation of extracellular matrix organization | 2/43 | 41/18670 | 0.004013 | 0.014555 | 0.006583 | RB1/IL6 | 2 |
| BP | GO:0023061 | | signal release | 5/43 | 462/18670 | 0.004029 | 0.014592 | 0.0066 | EGFR/IL6/RAF1/PRKCA/HIF1A | 5 |
| BP | GO:0072073 | | kidney epithelium development | 3/43 | 140/18670 | 0.00409 | 0.014793 | 0.00669 | VEGFA/BCL2/MYC | 3 |
| BP | GO:0008544 | | epidermis development | 5/43 | 464/18670 | 0.004104 | 0.014821 | 0.006703 | RELA/EGFR/BCL2/CASP3/TP63 | 5 |
| BP | GO:0034976 | | response to endoplasmic reticulum stress | 4/43 | 285/18670 | 0.004109 | 0.014822 | 0.006703 | CCND1/BCL2/CAV1/NFE2L2 | 4 |
| BP | GO:0051403 | | stress-activated MAPK cascade | 4/43 | 286/18670 | 0.004161 | 0.014987 | 0.006778 | EGFR/VEGFA/MYC/GSTP1 | 4 |
| BP | GO:0070585 | | protein localization to mitochondrion | 3/43 | 141/18670 | 0.004173 | 0.015009 | 0.006788 | BCL2/TP63/CASP8 | 3 |
| BP | GO:0010613 | | positive regulation of cardiac muscle hypertrophy | 2/43 | 42/18670 | 0.004208 | 0.015095 | 0.006827 | PRKCA/PARP1 | 2 |
| BP | GO:0042149 | | cellular response to glucose starvation | 2/43 | 42/18670 | 0.004208 | 0.015095 | 0.006827 | BCL2/NFE2L2 | 2 |
| BP | GO:0043254 | | regulation of protein complex assembly | 5/43 | 467/18670 | 0.004217 | 0.015108 | 0.006833 | VEGFA/RB1/RAF1/ICAM1/PARP1 | 5 |
| BP | GO:0038127 | | ERBB signaling pathway | 3/43 | 142/18670 | 0.004256 | 0.015184 | 0.006867 | EGFR/PRKCA/ERBB2 | 3 |
| BP | GO:0072006 | | nephron development | 3/43 | 142/18670 | 0.004256 | 0.015184 | 0.006867 | VEGFA/BCL2/MYC | 3 |
| BP | GO:1900542 | | regulation of purine nucleotide metabolic process | 3/43 | 142/18670 | 0.004256 | 0.015184 | 0.006867 | HIF1A/CCNB1/PARP1 | 3 |
| BP | GO:0002793 | | positive regulation of peptide secretion | 4/43 | 288/18670 | 0.004265 | 0.015196 | 0.006873 | ACHE/EGFR/IL6/HIF1A | 4 |
| BP | GO:0007162 | | negative regulation of cell adhesion | 4/43 | 289/18670 | 0.004318 | 0.015364 | 0.006948 | VEGFA/CASP3/ERBB2/CYP1B1 | 4 |
| BP | GO:0035296 | | regulation of tube diameter | 3/43 | 143/18670 | 0.00434 | 0.015382 | 0.006957 | EGFR/CAV1/ICAM1 | 3 |
| BP | GO:0050880 | | regulation of blood vessel size | 3/43 | 143/18670 | 0.00434 | 0.015382 | 0.006957 | EGFR/CAV1/ICAM1 | 3 |
| BP | GO:0097746 | | regulation of blood vessel diameter | 3/43 | 143/18670 | 0.00434 | 0.015382 | 0.006957 | EGFR/CAV1/ICAM1 | 3 |
| BP | GO:0034330 | | cell junction organization | 4/43 | 290/18670 | 0.004371 | 0.01547 | 0.006996 | VEGFA/BCL2/PRKCA/CAV1 | 4 |
| BP | GO:0006775 | | fat-soluble vitamin metabolic process | 2/43 | 43/18670 | 0.004407 | 0.015518 | 0.007018 | CYP3A4/CYP1A1 | 2 |
| BP | GO:0014742 | | positive regulation of muscle hypertrophy | 2/43 | 43/18670 | 0.004407 | 0.015518 | 0.007018 | PRKCA/PARP1 | 2 |
| BP | GO:0045687 | | positive regulation of glial cell differentiation | 2/43 | 43/18670 | 0.004407 | 0.015518 | 0.007018 | PPARG/RELA | 2 |
| BP | GO:0050708 | | regulation of protein secretion | 5/43 | 472/18670 | 0.004411 | 0.015518 | 0.007018 | ACHE/EGFR/IL6/PRKCA/HIF1A | 5 |
| BP | GO:0035150 | | regulation of tube size | 3/43 | 144/18670 | 0.004425 | 0.015518 | 0.007018 | EGFR/CAV1/ICAM1 | 3 |
| BP | GO:0060041 | | retina development in camera-type eye | 3/43 | 144/18670 | 0.004425 | 0.015518 | 0.007018 | ACHE/HIF1A/CYP1B1 | 3 |
| BP | GO:0071322 | | cellular response to carbohydrate stimulus | 3/43 | 144/18670 | 0.004425 | 0.015518 | 0.007018 | RAF1/HIF1A/ICAM1 | 3 |
| BP | GO:0090287 | | regulation of cellular response to growth factor stimulus | 4/43 | 292/18670 | 0.004478 | 0.015683 | 0.007093 | VEGFA/HIF1A/CAV1/RUNX2 | 4 |
| BP | GO:0000077 | | DNA damage checkpoint | 3/43 | 145/18670 | 0.004512 | 0.015758 | 0.007127 | CCND1/CCNB1/CHEK2 | 3 |
| BP | GO:0007605 | | sensory perception of sound | 3/43 | 145/18670 | 0.004512 | 0.015758 | 0.007127 | CASP3/ICAM1/BIRC5 | 3 |
| BP | GO:0006140 | | regulation of nucleotide metabolic process | 3/43 | 146/18670 | 0.004599 | 0.015954 | 0.007216 | HIF1A/CCNB1/PARP1 | 3 |
| BP | GO:0010463 | | mesenchymal cell proliferation | 2/43 | 44/18670 | 0.00461 | 0.015954 | 0.007216 | VEGFA/MYC | 2 |
| BP | GO:0014002 | | astrocyte development | 2/43 | 44/18670 | 0.00461 | 0.015954 | 0.007216 | EGFR/IL6 | 2 |
| BP | GO:0032965 | | regulation of collagen biosynthetic process | 2/43 | 44/18670 | 0.00461 | 0.015954 | 0.007216 | PPARG/IL6 | 2 |
| BP | GO:0034105 | | positive regulation of tissue remodeling | 2/43 | 44/18670 | 0.00461 | 0.015954 | 0.007216 | EGFR/PRKCA | 2 |
| BP | GO:0038084 | | vascular endothelial growth factor signaling pathway | 2/43 | 44/18670 | 0.00461 | 0.015954 | 0.007216 | VEGFA/HSPB1 | 2 |
| BP | GO:0070266 | | necroptotic process | 2/43 | 44/18670 | 0.00461 | 0.015954 | 0.007216 | CASP8/CAV1 | 2 |
| BP | GO:0048511 | | rhythmic process | 4/43 | 295/18670 | 0.004643 | 0.016048 | 0.007258 | PPARG/EGFR/CASP3/AHR | 4 |
| BP | GO:0050890 | | cognition | 4/43 | 296/18670 | 0.004699 | 0.016219 | 0.007335 | EGFR/FOS/CASP3/HIF1A | 4 |
| BP | GO:0035148 | | tube formation | 3/43 | 148/18670 | 0.004777 | 0.016467 | 0.007447 | VEGFA/CASP3/HIF1A | 3 |
| BP | GO:0002758 | | innate immune response-activating signal transduction | 4/43 | 298/18670 | 0.004812 | 0.016542 | 0.007482 | RELA/CASP8/RAF1/CAV1 | 4 |
| BP | GO:0048483 | | autonomic nervous system development | 2/43 | 45/18670 | 0.004817 | 0.016542 | 0.007482 | TP63/VCAM1 | 2 |
| BP | GO:0048538 | | thymus development | 2/43 | 45/18670 | 0.004817 | 0.016542 | 0.007482 | BCL2/RAF1 | 2 |
| BP | GO:0034248 | | regulation of cellular amide metabolic process | 5/43 | 483/18670 | 0.004861 | 0.016648 | 0.007529 | IL6/CASP3/ERBB2/HSPB1/NFE2L2 | 5 |
| BP | GO:0048592 | | eye morphogenesis | 3/43 | 149/18670 | 0.004867 | 0.016648 | 0.007529 | VEGFA/BCL2/HIF1A | 3 |
| BP | GO:0051092 | | positive regulation of NF-kappaB transcription factor activity | 3/43 | 149/18670 | 0.004867 | 0.016648 | 0.007529 | RELA/CAV1/ICAM1 | 3 |
| BP | GO:0016331 | | morphogenesis of embryonic epithelium | 3/43 | 150/18670 | 0.004958 | 0.016918 | 0.007651 | CASP3/TP63/HIF1A | 3 |
| BP | GO:0048754 | | branching morphogenesis of an epithelial tube | 3/43 | 150/18670 | 0.004958 | 0.016918 | 0.007651 | VEGFA/BCL2/MYC | 3 |
| BP | GO:0007157 | | heterophilic cell-cell adhesion via plasma membrane cell adhesion molecules | 2/43 | 46/18670 | 0.005029 | 0.017048 | 0.00771 | ICAM1/VCAM1 | 2 |
| BP | GO:0010883 | | regulation of lipid storage | 2/43 | 46/18670 | 0.005029 | 0.017048 | 0.00771 | PPARG/IL6 | 2 |
| BP | GO:0033628 | | regulation of cell adhesion mediated by integrin | 2/43 | 46/18670 | 0.005029 | 0.017048 | 0.00771 | PLAU/CYP1B1 | 2 |
| BP | GO:0048599 | | oocyte development | 2/43 | 46/18670 | 0.005029 | 0.017048 | 0.00771 | BCL2/CCNB1 | 2 |
| BP | GO:1904036 | | negative regulation of epithelial cell apoptotic process | 2/43 | 46/18670 | 0.005029 | 0.017048 | 0.00771 | ICAM1/NFE2L2 | 2 |
| BP | GO:0016999 | | antibiotic metabolic process | 3/43 | 151/18670 | 0.005051 | 0.017101 | 0.007734 | AKR1B1/EGFR/CYP1A1 | 3 |
| BP | GO:0001678 | | cellular glucose homeostasis | 3/43 | 152/18670 | 0.005144 | 0.017395 | 0.007867 | RAF1/HIF1A/ICAM1 | 3 |
| BP | GO:0050729 | | positive regulation of inflammatory response | 3/43 | 153/18670 | 0.005239 | 0.017578 | 0.00795 | EGFR/IL6/PTGER3 | 3 |
| BP | GO:0062013 | | positive regulation of small molecule metabolic process | 3/43 | 153/18670 | 0.005239 | 0.017578 | 0.00795 | PPARG/HIF1A/CCNB1 | 3 |
| BP | GO:0006953 | | acute-phase response | 2/43 | 47/18670 | 0.005245 | 0.017578 | 0.00795 | IL6/PTGER3 | 2 |
| BP | GO:0031952 | | regulation of protein autophosphorylation | 2/43 | 47/18670 | 0.005245 | 0.017578 | 0.00795 | VEGFA/CAV1 | 2 |
| BP | GO:0042551 | | neuron maturation | 2/43 | 47/18670 | 0.005245 | 0.017578 | 0.00795 | BCL2/RB1 | 2 |
| BP | GO:0045058 | | T cell selection | 2/43 | 47/18670 | 0.005245 | 0.017578 | 0.00795 | BCL2/IL6 | 2 |
| BP | GO:1903580 | | positive regulation of ATP metabolic process | 2/43 | 47/18670 | 0.005245 | 0.017578 | 0.00795 | HIF1A/CCNB1 | 2 |
| BP | GO:1903707 | | negative regulation of hemopoiesis | 3/43 | 155/18670 | 0.005431 | 0.018179 | 0.008222 | ERBB2/MYC/NFE2L2 | 3 |
| BP | GO:0051972 | | regulation of telomerase activity | 2/43 | 48/18670 | 0.005465 | 0.018269 | 0.008262 | PPARG/MYC | 2 |
| BP | GO:0051053 | | negative regulation of DNA metabolic process | 3/43 | 156/18670 | 0.005529 | 0.01846 | 0.008349 | PPARG/PARP1/CHEK2 | 3 |
| BP | GO:0050900 | | leukocyte migration | 5/43 | 499/18670 | 0.005572 | 0.018582 | 0.008404 | VEGFA/IL6/CAV1/ICAM1/VCAM1 | 5 |
| BP | GO:0002791 | | regulation of peptide secretion | 5/43 | 500/18670 | 0.005619 | 0.018695 | 0.008455 | ACHE/EGFR/IL6/PRKCA/HIF1A | 5 |
| BP | GO:0031570 | | DNA integrity checkpoint | 3/43 | 157/18670 | 0.005628 | 0.018695 | 0.008455 | CCND1/CCNB1/CHEK2 | 3 |
| BP | GO:0043271 | | negative regulation of ion transport | 3/43 | 157/18670 | 0.005628 | 0.018695 | 0.008455 | BCL2/CAV1/ICAM1 | 3 |
| BP | GO:0003254 | | regulation of membrane depolarization | 2/43 | 49/18670 | 0.005689 | 0.018806 | 0.008505 | BCL2/PARP1 | 2 |
| BP | GO:0010712 | | regulation of collagen metabolic process | 2/43 | 49/18670 | 0.005689 | 0.018806 | 0.008505 | PPARG/IL6 | 2 |
| BP | GO:0043124 | | negative regulation of I-kappaB kinase/NF-kappaB signaling | 2/43 | 49/18670 | 0.005689 | 0.018806 | 0.008505 | CASP8/GSTP1 | 2 |
| BP | GO:0097300 | | programmed necrotic cell death | 2/43 | 49/18670 | 0.005689 | 0.018806 | 0.008505 | CASP8/CAV1 | 2 |
| BP | GO:0032873 | | negative regulation of stress-activated MAPK cascade | 2/43 | 50/18670 | 0.005918 | 0.019512 | 0.008825 | MYC/GSTP1 | 2 |
| BP | GO:0070303 | | negative regulation of stress-activated protein kinase signaling cascade | 2/43 | 50/18670 | 0.005918 | 0.019512 | 0.008825 | MYC/GSTP1 | 2 |
| BP | GO:0046661 | | male sex differentiation | 3/43 | 160/18670 | 0.00593 | 0.019529 | 0.008832 | CCND1/BCL2/ICAM1 | 3 |
| BP | GO:0002218 | | activation of innate immune response | 4/43 | 319/18670 | 0.00611 | 0.020098 | 0.009089 | RELA/CASP8/RAF1/CAV1 | 4 |
| BP | GO:0009994 | | oocyte differentiation | 2/43 | 51/18670 | 0.00615 | 0.020178 | 0.009126 | BCL2/CCNB1 | 2 |
| BP | GO:0030071 | | regulation of mitotic metaphase/anaphase transition | 2/43 | 51/18670 | 0.00615 | 0.020178 | 0.009126 | RB1/CCNB1 | 2 |
| BP | GO:0006633 | | fatty acid biosynthetic process | 3/43 | 164/18670 | 0.006349 | 0.020775 | 0.009396 | PTGS1/CYP3A4/CYP1A1 | 3 |
| BP | GO:0006998 | | nuclear envelope organization | 2/43 | 52/18670 | 0.006387 | 0.020775 | 0.009396 | PRKCA/CCNB1 | 2 |
| BP | GO:0031529 | | ruffle organization | 2/43 | 52/18670 | 0.006387 | 0.020775 | 0.009396 | CAV1/ICAM1 | 2 |
| BP | GO:0032330 | | regulation of chondrocyte differentiation | 2/43 | 52/18670 | 0.006387 | 0.020775 | 0.009396 | RELA/RUNX2 | 2 |
| BP | GO:0032731 | | positive regulation of interleukin-1 beta production | 2/43 | 52/18670 | 0.006387 | 0.020775 | 0.009396 | CASP8/HSPB1 | 2 |
| BP | GO:2000179 | | positive regulation of neural precursor cell proliferation | 2/43 | 52/18670 | 0.006387 | 0.020775 | 0.009396 | VEGFA/HIF1A | 2 |
| BP | GO:2000677 | | regulation of transcription regulatory region DNA binding | 2/43 | 52/18670 | 0.006387 | 0.020775 | 0.009396 | RB1/PARP1 | 2 |
| BP | GO:0050680 | | negative regulation of epithelial cell proliferation | 3/43 | 165/18670 | 0.006456 | 0.020948 | 0.009474 | PPARG/RB1/CAV1 | 3 |
| BP | GO:0050954 | | sensory perception of mechanical stimulus | 3/43 | 165/18670 | 0.006456 | 0.020948 | 0.009474 | CASP3/ICAM1/BIRC5 | 3 |
| BP | GO:0000186 | | activation of MAPKK activity | 2/43 | 53/18670 | 0.006628 | 0.021349 | 0.009655 | EGFR/RAF1 | 2 |
| BP | GO:0010043 | | response to zinc ion | 2/43 | 53/18670 | 0.006628 | 0.021349 | 0.009655 | VCAM1/PARP1 | 2 |
| BP | GO:0031638 | | zymogen activation | 2/43 | 53/18670 | 0.006628 | 0.021349 | 0.009655 | PLAU/CASP8 | 2 |
| BP | GO:0032964 | | collagen biosynthetic process | 2/43 | 53/18670 | 0.006628 | 0.021349 | 0.009655 | PPARG/IL6 | 2 |
| BP | GO:1901185 | | negative regulation of ERBB signaling pathway | 2/43 | 53/18670 | 0.006628 | 0.021349 | 0.009655 | EGFR/ERBB2 | 2 |
| BP | GO:1902099 | | regulation of metaphase/anaphase transition of cell cycle | 2/43 | 53/18670 | 0.006628 | 0.021349 | 0.009655 | RB1/CCNB1 | 2 |
| BP | GO:0010506 | | regulation of autophagy | 4/43 | 327/18670 | 0.006661 | 0.021419 | 0.009687 | BCL2/CASP3/HIF1A/HSPB1 | 4 |
| BP | GO:0033209 | | tumor necrosis factor-mediated signaling pathway | 3/43 | 167/18670 | 0.006674 | 0.021419 | 0.009687 | RELA/CASP8/GSTP1 | 3 |
| BP | GO:1901568 | | fatty acid derivative metabolic process | 3/43 | 167/18670 | 0.006674 | 0.021419 | 0.009687 | PTGS1/CYP1A1/CYP1B1 | 3 |
| BP | GO:0016485 | | protein processing | 4/43 | 328/18670 | 0.006732 | 0.021554 | 0.009748 | PLAU/CASP3/CASP8/PARP1 | 4 |
| BP | GO:0019058 | | viral life cycle | 4/43 | 328/18670 | 0.006732 | 0.021554 | 0.009748 | EGFR/BCL2/CAV1/ICAM1 | 4 |
| BP | GO:0051302 | | regulation of cell division | 3/43 | 168/18670 | 0.006784 | 0.021695 | 0.009812 | VEGFA/TP63/MYC | 3 |
| BP | GO:0007091 | | metaphase/anaphase transition of mitotic cell cycle | 2/43 | 54/18670 | 0.006873 | 0.021951 | 0.009928 | RB1/CCNB1 | 2 |
| BP | GO:0050728 | | negative regulation of inflammatory response | 3/43 | 169/18670 | 0.006896 | 0.021999 | 0.009949 | PPARG/RB1/GSTP1 | 3 |
| BP | GO:0008202 | | steroid metabolic process | 4/43 | 331/18670 | 0.006948 | 0.02214 | 0.010013 | AKR1B1/CYP3A4/CYP1A1/CYP1B1 | 4 |
| BP | GO:0007051 | | spindle organization | 3/43 | 170/18670 | 0.007009 | 0.022305 | 0.010088 | BIRC5/CCNB1/CHEK2 | 3 |
| BP | GO:0051251 | | positive regulation of lymphocyte activation | 4/43 | 334/18670 | 0.007169 | 0.022789 | 0.010306 | BCL2/IL6/CAV1/VCAM1 | 4 |
| BP | GO:0055002 | | striated muscle cell development | 3/43 | 173/18670 | 0.007353 | 0.023302 | 0.010539 | VEGFA/BCL2/CCNB1 | 3 |
| BP | GO:0006977 | | DNA damage response, signal transduction by p53 class mediator resulting in cell cycle arrest | 2/43 | 56/18670 | 0.007375 | 0.023302 | 0.010539 | CCNB1/CHEK2 | 2 |
| BP | GO:0044784 | | metaphase/anaphase transition of cell cycle | 2/43 | 56/18670 | 0.007375 | 0.023302 | 0.010539 | RB1/CCNB1 | 2 |
| BP | GO:0090183 | | regulation of kidney development | 2/43 | 56/18670 | 0.007375 | 0.023302 | 0.010539 | VEGFA/MYC | 2 |
| BP | GO:0090307 | | mitotic spindle assembly | 2/43 | 56/18670 | 0.007375 | 0.023302 | 0.010539 | BIRC5/CHEK2 | 2 |
| BP | GO:2001252 | | positive regulation of chromosome organization | 3/43 | 174/18670 | 0.007471 | 0.023577 | 0.010663 | VEGFA/RB1/CCNB1 | 3 |
| BP | GO:0010965 | | regulation of mitotic sister chromatid separation | 2/43 | 57/18670 | 0.007632 | 0.023886 | 0.010803 | RB1/CCNB1 | 2 |
| BP | GO:0042743 | | hydrogen peroxide metabolic process | 2/43 | 57/18670 | 0.007632 | 0.023886 | 0.010803 | EGFR/CYP1A1 | 2 |
| BP | GO:0045981 | | positive regulation of nucleotide metabolic process | 2/43 | 57/18670 | 0.007632 | 0.023886 | 0.010803 | HIF1A/CCNB1 | 2 |
| BP | GO:0071398 | | cellular response to fatty acid | 2/43 | 57/18670 | 0.007632 | 0.023886 | 0.010803 | PPARG/CCNB1 | 2 |
| BP | GO:0072431 | | signal transduction involved in mitotic G1 DNA damage checkpoint | 2/43 | 57/18670 | 0.007632 | 0.023886 | 0.010803 | CCNB1/CHEK2 | 2 |
| BP | GO:1900544 | | positive regulation of purine nucleotide metabolic process | 2/43 | 57/18670 | 0.007632 | 0.023886 | 0.010803 | HIF1A/CCNB1 | 2 |
| BP | GO:1902400 | | intracellular signal transduction involved in G1 DNA damage checkpoint | 2/43 | 57/18670 | 0.007632 | 0.023886 | 0.010803 | CCNB1/CHEK2 | 2 |
| BP | GO:0006986 | | response to unfolded protein | 3/43 | 176/18670 | 0.007708 | 0.024097 | 0.010898 | CCND1/HSPB1/NFE2L2 | 3 |
| BP | GO:0072330 | | monocarboxylic acid biosynthetic process | 4/43 | 343/18670 | 0.00786 | 0.02453 | 0.011094 | PTGS1/HIF1A/CYP3A4/CYP1A1 | 4 |
| BP | GO:0030520 | | intracellular estrogen receptor signaling pathway | 2/43 | 58/18670 | 0.007893 | 0.02453 | 0.011094 | TP63/PARP1 | 2 |
| BP | GO:0032722 | | positive regulation of chemokine production | 2/43 | 58/18670 | 0.007893 | 0.02453 | 0.011094 | IL6/HIF1A | 2 |
| BP | GO:0061900 | | glial cell activation | 2/43 | 58/18670 | 0.007893 | 0.02453 | 0.011094 | EGFR/IL6 | 2 |
| BP | GO:0098930 | | axonal transport | 2/43 | 58/18670 | 0.007893 | 0.02453 | 0.011094 | HIF1A/HSPB1 | 2 |
| BP | GO:0010721 | | negative regulation of cell development | 4/43 | 344/18670 | 0.007939 | 0.024644 | 0.011146 | VEGFA/BCL2/IL6/COL3A1 | 4 |
| BP | GO:0001885 | | endothelial cell development | 2/43 | 59/18670 | 0.008158 | 0.025089 | 0.011347 | VEGFA/ICAM1 | 2 |
| BP | GO:0032732 | | positive regulation of interleukin-1 production | 2/43 | 59/18670 | 0.008158 | 0.025089 | 0.011347 | CASP8/HSPB1 | 2 |
| BP | GO:0042130 | | negative regulation of T cell proliferation | 2/43 | 59/18670 | 0.008158 | 0.025089 | 0.011347 | CASP3/ERBB2 | 2 |
| BP | GO:0070527 | | platelet aggregation | 2/43 | 59/18670 | 0.008158 | 0.025089 | 0.011347 | PRKCA/HSPB1 | 2 |
| BP | GO:0072413 | | signal transduction involved in mitotic cell cycle checkpoint | 2/43 | 59/18670 | 0.008158 | 0.025089 | 0.011347 | CCNB1/CHEK2 | 2 |
| BP | GO:1902402 | | signal transduction involved in mitotic DNA damage checkpoint | 2/43 | 59/18670 | 0.008158 | 0.025089 | 0.011347 | CCNB1/CHEK2 | 2 |
| BP | GO:1902403 | | signal transduction involved in mitotic DNA integrity checkpoint | 2/43 | 59/18670 | 0.008158 | 0.025089 | 0.011347 | CCNB1/CHEK2 | 2 |
| BP | GO:2000351 | | regulation of endothelial cell apoptotic process | 2/43 | 59/18670 | 0.008158 | 0.025089 | 0.011347 | ICAM1/NFE2L2 | 2 |
| BP | GO:0071346 | | cellular response to interferon-gamma | 3/43 | 180/18670 | 0.008197 | 0.02515 | 0.011374 | PPARG/ICAM1/VCAM1 | 3 |
| BP | GO:1901796 | | regulation of signal transduction by p53 class mediator | 3/43 | 180/18670 | 0.008197 | 0.02515 | 0.011374 | BCL2/TP63/CHEK2 | 3 |
| BP | GO:0010565 | | regulation of cellular ketone metabolic process | 3/43 | 181/18670 | 0.008322 | 0.025474 | 0.011521 | PPARG/CAV1/NQO1 | 3 |
| BP | GO:0022408 | | negative regulation of cell-cell adhesion | 3/43 | 181/18670 | 0.008322 | 0.025474 | 0.011521 | VEGFA/CASP3/ERBB2 | 3 |
| BP | GO:0051306 | | mitotic sister chromatid separation | 2/43 | 60/18670 | 0.008427 | 0.025767 | 0.011653 | RB1/CCNB1 | 2 |
| BP | GO:0006338 | | chromatin remodeling | 3/43 | 182/18670 | 0.008448 | 0.0258 | 0.011668 | RB1/TP63/MYC | 3 |
| BP | GO:0006879 | | cellular iron ion homeostasis | 2/43 | 61/18670 | 0.0087 | 0.026337 | 0.011911 | HIF1A/MYC | 2 |
| BP | GO:0007405 | | neuroblast proliferation | 2/43 | 61/18670 | 0.0087 | 0.026337 | 0.011911 | VEGFA/HIF1A | 2 |
| BP | GO:0010803 | | regulation of tumor necrosis factor-mediated signaling pathway | 2/43 | 61/18670 | 0.0087 | 0.026337 | 0.011911 | CASP8/GSTP1 | 2 |
| BP | GO:0045123 | | cellular extravasation | 2/43 | 61/18670 | 0.0087 | 0.026337 | 0.011911 | ICAM1/VCAM1 | 2 |
| BP | GO:1905953 | | negative regulation of lipid localization | 2/43 | 61/18670 | 0.0087 | 0.026337 | 0.011911 | PPARG/IL6 | 2 |
| BP | GO:2001244 | | positive regulation of intrinsic apoptotic signaling pathway | 2/43 | 61/18670 | 0.0087 | 0.026337 | 0.011911 | BCL2/CAV1 | 2 |
| BP | GO:0043409 | | negative regulation of MAPK cascade | 3/43 | 184/18670 | 0.008703 | 0.026337 | 0.011911 | CAV1/MYC/GSTP1 | 3 |
| BP | GO:0048639 | | positive regulation of developmental growth | 3/43 | 184/18670 | 0.008703 | 0.026337 | 0.011911 | VEGFA/BCL2/CCNB1 | 3 |
| BP | GO:0022900 | | electron transport chain | 3/43 | 186/18670 | 0.008963 | 0.027012 | 0.012217 | AKR1B1/CCNB1/NQO1 | 3 |
| BP | GO:0055001 | | muscle cell development | 3/43 | 186/18670 | 0.008963 | 0.027012 | 0.012217 | VEGFA/BCL2/CCNB1 | 3 |
| BP | GO:0002753 | | cytoplasmic pattern recognition receptor signaling pathway | 2/43 | 62/18670 | 0.008977 | 0.027012 | 0.012217 | RELA/CASP8 | 2 |
| BP | GO:0070265 | | necrotic cell death | 2/43 | 62/18670 | 0.008977 | 0.027012 | 0.012217 | CASP8/CAV1 | 2 |
| BP | GO:1905818 | | regulation of chromosome separation | 2/43 | 62/18670 | 0.008977 | 0.027012 | 0.012217 | RB1/CCNB1 | 2 |
| BP | GO:0019933 | | cAMP-mediated signaling | 3/43 | 187/18670 | 0.009095 | 0.027336 | 0.012363 | PRKCA/PTGER3/AHR | 3 |
| BP | GO:0042982 | | amyloid precursor protein metabolic process | 2/43 | 63/18670 | 0.009258 | 0.027763 | 0.012556 | ACHE/CASP3 | 2 |
| BP | GO:0046686 | | response to cadmium ion | 2/43 | 63/18670 | 0.009258 | 0.027763 | 0.012556 | EGFR/FOS | 2 |
| BP | GO:0031331 | | positive regulation of cellular catabolic process | 4/43 | 361/18670 | 0.009369 | 0.028065 | 0.012693 | IL6/HIF1A/CAV1/NFE2L2 | 4 |
| BP | GO:0035773 | | insulin secretion involved in cellular response to glucose stimulus | 2/43 | 64/18670 | 0.009543 | 0.028552 | 0.012913 | RAF1/HIF1A | 2 |
| BP | GO:0042594 | | response to starvation | 3/43 | 191/18670 | 0.009633 | 0.028773 | 0.013013 | PPARG/BCL2/NFE2L2 | 3 |
| BP | GO:0006066 | | alcohol metabolic process | 4/43 | 364/18670 | 0.009638 | 0.028773 | 0.013013 | AKR1B1/CYP3A4/CYP1A1/CYP1B1 | 4 |
| BP | GO:0032102 | | negative regulation of response to external stimulus | 4/43 | 365/18670 | 0.009729 | 0.029011 | 0.013121 | PPARG/PLAU/RB1/GSTP1 | 4 |
| BP | GO:0007565 | | female pregnancy | 3/43 | 192/18670 | 0.00977 | 0.029037 | 0.013132 | AKR1B1/BCL2/FOS | 3 |
| BP | GO:0044272 | | sulfur compound biosynthetic process | 3/43 | 192/18670 | 0.00977 | 0.029037 | 0.013132 | GSTP1/NFE2L2/GSTM1 | 3 |
| BP | GO:0050731 | | positive regulation of peptidyl-tyrosine phosphorylation | 3/43 | 192/18670 | 0.00977 | 0.029037 | 0.013132 | VEGFA/IL6/ICAM1 | 3 |
| BP | GO:0045739 | | positive regulation of DNA repair | 2/43 | 65/18670 | 0.009831 | 0.029153 | 0.013185 | EGFR/PARP1 | 2 |
| BP | GO:0072577 | | endothelial cell apoptotic process | 2/43 | 65/18670 | 0.009831 | 0.029153 | 0.013185 | ICAM1/NFE2L2 | 2 |
| BP | GO:0007219 | | Notch signaling pathway | 3/43 | 193/18670 | 0.009909 | 0.02935 | 0.013274 | EGFR/TP63/MYC | 3 |
| BP | GO:0045428 | | regulation of nitric oxide biosynthetic process | 2/43 | 66/18670 | 0.010124 | 0.029886 | 0.013516 | CAV1/ICAM1 | 2 |
| BP | GO:0051926 | | negative regulation of calcium ion transport | 2/43 | 66/18670 | 0.010124 | 0.029886 | 0.013516 | BCL2/ICAM1 | 2 |
| BP | GO:1904888 | | cranial skeletal system development | 2/43 | 66/18670 | 0.010124 | 0.029886 | 0.013516 | TP63/RUNX2 | 2 |
| BP | GO:0002685 | | regulation of leukocyte migration | 3/43 | 196/18670 | 0.010331 | 0.030465 | 0.013778 | VEGFA/IL6/ICAM1 | 3 |
| BP | GO:0048747 | | muscle fiber development | 2/43 | 67/18670 | 0.01042 | 0.030693 | 0.013881 | VEGFA/BCL2 | 2 |
| BP | GO:0002221 | | pattern recognition receptor signaling pathway | 3/43 | 197/18670 | 0.010474 | 0.030818 | 0.013938 | RELA/CASP8/CAV1 | 3 |
| BP | GO:0033047 | | regulation of mitotic sister chromatid segregation | 2/43 | 68/18670 | 0.01072 | 0.031403 | 0.014202 | RB1/CCNB1 | 2 |
| BP | GO:0035914 | | skeletal muscle cell differentiation | 2/43 | 68/18670 | 0.01072 | 0.031403 | 0.014202 | FOS/RB1 | 2 |
| BP | GO:0050891 | | multicellular organismal water homeostasis | 2/43 | 68/18670 | 0.01072 | 0.031403 | 0.014202 | AKR1B1/TP63 | 2 |
| BP | GO:0071230 | | cellular response to amino acid stimulus | 2/43 | 68/18670 | 0.01072 | 0.031403 | 0.014202 | EGFR/COL3A1 | 2 |
| BP | GO:0034341 | | response to interferon-gamma | 3/43 | 199/18670 | 0.010764 | 0.031461 | 0.014229 | PPARG/ICAM1/VCAM1 | 3 |
| BP | GO:0035966 | | response to topologically incorrect protein | 3/43 | 199/18670 | 0.010764 | 0.031461 | 0.014229 | CCND1/HSPB1/NFE2L2 | 3 |
| BP | GO:0008088 | | axo-dendritic transport | 2/43 | 69/18670 | 0.011024 | 0.032151 | 0.014541 | HIF1A/HSPB1 | 2 |
| BP | GO:0042698 | | ovulation cycle | 2/43 | 69/18670 | 0.011024 | 0.032151 | 0.014541 | EGFR/CASP3 | 2 |
| BP | GO:0002696 | | positive regulation of leukocyte activation | 4/43 | 380/18670 | 0.011157 | 0.032501 | 0.014699 | BCL2/IL6/CAV1/VCAM1 | 4 |
| BP | GO:0006575 | | cellular modified amino acid metabolic process | 3/43 | 202/18670 | 0.011207 | 0.032577 | 0.014733 | GSTP1/NFE2L2/GSTM1 | 3 |
| BP | GO:0050870 | | positive regulation of T cell activation | 3/43 | 202/18670 | 0.011207 | 0.032577 | 0.014733 | IL6/CAV1/VCAM1 | 3 |
| BP | GO:0030900 | | forebrain development | 4/43 | 381/18670 | 0.011256 | 0.032648 | 0.014766 | EGFR/CASP3/HIF1A/COL3A1 | 4 |
| BP | GO:0045089 | | positive regulation of innate immune response | 4/43 | 381/18670 | 0.011256 | 0.032648 | 0.014766 | RELA/CASP8/RAF1/CAV1 | 4 |
| BP | GO:0033077 | | T cell differentiation in thymus | 2/43 | 70/18670 | 0.011332 | 0.032725 | 0.0148 | BCL2/ERBB2 | 2 |
| BP | GO:0060395 | | SMAD protein signal transduction | 2/43 | 70/18670 | 0.011332 | 0.032725 | 0.0148 | FOS/PARP1 | 2 |
| BP | GO:0070988 | | demethylation | 2/43 | 70/18670 | 0.011332 | 0.032725 | 0.0148 | CYP3A4/CYP1A1 | 2 |
| BP | GO:0072091 | | regulation of stem cell proliferation | 2/43 | 70/18670 | 0.011332 | 0.032725 | 0.0148 | VEGFA/HIF1A | 2 |
| BP | GO:0035019 | | somatic stem cell population maintenance | 2/43 | 72/18670 | 0.011959 | 0.034378 | 0.015548 | TP63/RAF1 | 2 |
| BP | GO:0051881 | | regulation of mitochondrial membrane potential | 2/43 | 72/18670 | 0.011959 | 0.034378 | 0.015548 | BCL2/PARP1 | 2 |
| BP | GO:0061035 | | regulation of cartilage development | 2/43 | 72/18670 | 0.011959 | 0.034378 | 0.015548 | RELA/RUNX2 | 2 |
| BP | GO:0061180 | | mammary gland epithelium development | 2/43 | 72/18670 | 0.011959 | 0.034378 | 0.015548 | CCND1/HIF1A | 2 |
| BP | GO:0030073 | | insulin secretion | 3/43 | 207/18670 | 0.011969 | 0.034378 | 0.015548 | RAF1/PRKCA/HIF1A | 3 |
| BP | GO:0007623 | | circadian rhythm | 3/43 | 208/18670 | 0.012125 | 0.03475 | 0.015716 | PPARG/EGFR/AHR | 3 |
| BP | GO:0090276 | | regulation of peptide hormone secretion | 3/43 | 208/18670 | 0.012125 | 0.03475 | 0.015716 | EGFR/PRKCA/HIF1A | 3 |
| BP | GO:0032945 | | negative regulation of mononuclear cell proliferation | 2/43 | 73/18670 | 0.012278 | 0.035011 | 0.015834 | CASP3/ERBB2 | 2 |
| BP | GO:0050672 | | negative regulation of lymphocyte proliferation | 2/43 | 73/18670 | 0.012278 | 0.035011 | 0.015834 | CASP3/ERBB2 | 2 |
| BP | GO:0072401 | | signal transduction involved in DNA integrity checkpoint | 2/43 | 73/18670 | 0.012278 | 0.035011 | 0.015834 | CCNB1/CHEK2 | 2 |
| BP | GO:0072422 | | signal transduction involved in DNA damage checkpoint | 2/43 | 73/18670 | 0.012278 | 0.035011 | 0.015834 | CCNB1/CHEK2 | 2 |
| BP | GO:0051216 | | cartilage development | 3/43 | 209/18670 | 0.012282 | 0.035011 | 0.015834 | RELA/HIF1A/RUNX2 | 3 |
| BP | GO:0006081 | | cellular aldehyde metabolic process | 2/43 | 74/18670 | 0.012601 | 0.03578 | 0.016182 | RELA/CYP1B1 | 2 |
| BP | GO:0030104 | | water homeostasis | 2/43 | 74/18670 | 0.012601 | 0.03578 | 0.016182 | AKR1B1/TP63 | 2 |
| BP | GO:0072395 | | signal transduction involved in cell cycle checkpoint | 2/43 | 74/18670 | 0.012601 | 0.03578 | 0.016182 | CCNB1/CHEK2 | 2 |
| BP | GO:0050867 | | positive regulation of cell activation | 4/43 | 394/18670 | 0.012606 | 0.03578 | 0.016182 | BCL2/IL6/CAV1/VCAM1 | 4 |
| BP | GO:0007422 | | peripheral nervous system development | 2/43 | 75/18670 | 0.012927 | 0.036509 | 0.016512 | RELA/ERBB2 | 2 |
| BP | GO:0010611 | | regulation of cardiac muscle hypertrophy | 2/43 | 75/18670 | 0.012927 | 0.036509 | 0.016512 | PRKCA/PARP1 | 2 |
| BP | GO:0046173 | | polyol biosynthetic process | 2/43 | 75/18670 | 0.012927 | 0.036509 | 0.016512 | AKR1B1/CYP3A4 | 2 |
| BP | GO:0150076 | | neuroinflammatory response | 2/43 | 75/18670 | 0.012927 | 0.036509 | 0.016512 | EGFR/IL6 | 2 |
| BP | GO:0051604 | | protein maturation | 4/43 | 397/18670 | 0.012932 | 0.036509 | 0.016512 | PLAU/CASP3/CASP8/PARP1 | 4 |
| BP | GO:1903532 | | positive regulation of secretion by cell | 4/43 | 399/18670 | 0.013152 | 0.037091 | 0.016775 | ACHE/EGFR/IL6/HIF1A | 4 |
| BP | GO:0010810 | | regulation of cell-substrate adhesion | 3/43 | 215/18670 | 0.01325 | 0.037191 | 0.01682 | VEGFA/BCL2/PLAU | 3 |
| BP | GO:0019935 | | cyclic-nucleotide-mediated signaling | 3/43 | 215/18670 | 0.01325 | 0.037191 | 0.01682 | PRKCA/PTGER3/AHR | 3 |
| BP | GO:0070374 | | positive regulation of ERK1 and ERK2 cascade | 3/43 | 215/18670 | 0.01325 | 0.037191 | 0.01682 | EGFR/PRKCA/ICAM1 | 3 |
| BP | GO:0001937 | | negative regulation of endothelial cell proliferation | 2/43 | 76/18670 | 0.013257 | 0.037191 | 0.01682 | PPARG/CAV1 | 2 |
| BP | GO:0048844 | | artery morphogenesis | 2/43 | 76/18670 | 0.013257 | 0.037191 | 0.01682 | VEGFA/COL3A1 | 2 |
| BP | GO:1901224 | | positive regulation of NIK/NF-kappaB signaling | 2/43 | 77/18670 | 0.013591 | 0.038087 | 0.017225 | RELA/EGFR | 2 |
| BP | GO:0048762 | | mesenchymal cell differentiation | 3/43 | 219/18670 | 0.013918 | 0.038746 | 0.017523 | BCL2/IL6/HIF1A | 3 |
| BP | GO:0008306 | | associative learning | 2/43 | 78/18670 | 0.013929 | 0.038746 | 0.017523 | FOS/HIF1A | 2 |
| BP | GO:0008589 | | regulation of smoothened signaling pathway | 2/43 | 78/18670 | 0.013929 | 0.038746 | 0.017523 | RB1/RUNX2 | 2 |
| BP | GO:0014743 | | regulation of muscle hypertrophy | 2/43 | 78/18670 | 0.013929 | 0.038746 | 0.017523 | PRKCA/PARP1 | 2 |
| BP | GO:0030433 | | ubiquitin-dependent ERAD pathway | 2/43 | 78/18670 | 0.013929 | 0.038746 | 0.017523 | CAV1/NFE2L2 | 2 |
| BP | GO:0033143 | | regulation of intracellular steroid hormone receptor signaling pathway | 2/43 | 78/18670 | 0.013929 | 0.038746 | 0.017523 | TP63/PARP1 | 2 |
| BP | GO:0043407 | | negative regulation of MAP kinase activity | 2/43 | 78/18670 | 0.013929 | 0.038746 | 0.017523 | CAV1/GSTP1 | 2 |
| BP | GO:0000280 | | nuclear division | 4/43 | 407/18670 | 0.014056 | 0.03906 | 0.017665 | RB1/BIRC5/CCNB1/CHEK2 | 4 |
| BP | GO:0001570 | | vasculogenesis | 2/43 | 79/18670 | 0.01427 | 0.039488 | 0.017859 | VEGFA/CAV1 | 2 |
| BP | GO:0003151 | | outflow tract morphogenesis | 2/43 | 79/18670 | 0.01427 | 0.039488 | 0.017859 | VEGFA/HIF1A | 2 |
| BP | GO:0055072 | | iron ion homeostasis | 2/43 | 79/18670 | 0.01427 | 0.039488 | 0.017859 | HIF1A/MYC | 2 |
| BP | GO:0071260 | | cellular response to mechanical stimulus | 2/43 | 79/18670 | 0.01427 | 0.039488 | 0.017859 | EGFR/CASP8 | 2 |
| BP | GO:0019216 | | regulation of lipid metabolic process | 4/43 | 410/18670 | 0.014405 | 0.039821 | 0.018009 | PPARG/RB1/CAV1/CYP1A1 | 4 |
| BP | GO:0045444 | | fat cell differentiation | 3/43 | 223/18670 | 0.014606 | 0.040315 | 0.018233 | PPARG/CCND1/IL6 | 3 |
| BP | GO:0033045 | | regulation of sister chromatid segregation | 2/43 | 80/18670 | 0.014614 | 0.040315 | 0.018233 | RB1/CCNB1 | 2 |
| BP | GO:0007045 | | cell-substrate adherens junction assembly | 2/43 | 81/18670 | 0.014963 | 0.040935 | 0.018513 | VEGFA/BCL2 | 2 |
| BP | GO:0032204 | | regulation of telomere maintenance | 2/43 | 81/18670 | 0.014963 | 0.040935 | 0.018513 | MYC/PARP1 | 2 |
| BP | GO:0032436 | | positive regulation of proteasomal ubiquitin-dependent protein catabolic process | 2/43 | 81/18670 | 0.014963 | 0.040935 | 0.018513 | CAV1/NFE2L2 | 2 |
| BP | GO:0034109 | | homotypic cell-cell adhesion | 2/43 | 81/18670 | 0.014963 | 0.040935 | 0.018513 | PRKCA/HSPB1 | 2 |
| BP | GO:0048041 | | focal adhesion assembly | 2/43 | 81/18670 | 0.014963 | 0.040935 | 0.018513 | VEGFA/BCL2 | 2 |
| BP | GO:0048708 | | astrocyte differentiation | 2/43 | 81/18670 | 0.014963 | 0.040935 | 0.018513 | EGFR/IL6 | 2 |
| BP | GO:0110110 | | positive regulation of animal organ morphogenesis | 2/43 | 81/18670 | 0.014963 | 0.040935 | 0.018513 | VEGFA/MYC | 2 |
| BP | GO:2001021 | | negative regulation of response to DNA damage stimulus | 2/43 | 81/18670 | 0.014963 | 0.040935 | 0.018513 | BCL2/CHEK2 | 2 |
| BP | GO:0030279 | | negative regulation of ossification | 2/43 | 82/18670 | 0.015315 | 0.041812 | 0.01891 | BCL2/HIF1A | 2 |
| BP | GO:0071158 | | positive regulation of cell cycle arrest | 2/43 | 82/18670 | 0.015315 | 0.041812 | 0.01891 | CCNB1/CHEK2 | 2 |
| BP | GO:0042509 | | regulation of tyrosine phosphorylation of STAT protein | 2/43 | 83/18670 | 0.01567 | 0.042738 | 0.019329 | IL6/CAV1 | 2 |
| BP | GO:0009896 | | positive regulation of catabolic process | 4/43 | 423/18670 | 0.015982 | 0.043544 | 0.019693 | IL6/HIF1A/CAV1/NFE2L2 | 4 |
| BP | GO:0045445 | | myoblast differentiation | 2/43 | 84/18670 | 0.016029 | 0.043582 | 0.019711 | RB1/IGFBP3 | 2 |
| BP | GO:1905897 | | regulation of response to endoplasmic reticulum stress | 2/43 | 84/18670 | 0.016029 | 0.043582 | 0.019711 | CAV1/NFE2L2 | 2 |
| BP | GO:0014031 | | mesenchymal cell development | 2/43 | 85/18670 | 0.016391 | 0.044431 | 0.020095 | BCL2/HIF1A | 2 |
| BP | GO:1904705 | | regulation of vascular smooth muscle cell proliferation | 2/43 | 85/18670 | 0.016391 | 0.044431 | 0.020095 | PPARG/GSTP1 | 2 |
| BP | GO:1990874 | | vascular smooth muscle cell proliferation | 2/43 | 85/18670 | 0.016391 | 0.044431 | 0.020095 | PPARG/GSTP1 | 2 |
| BP | GO:0051047 | | positive regulation of secretion | 4/43 | 428/18670 | 0.016616 | 0.044994 | 0.020349 | ACHE/EGFR/IL6/HIF1A | 4 |
| BP | GO:0007260 | | tyrosine phosphorylation of STAT protein | 2/43 | 86/18670 | 0.016757 | 0.045285 | 0.02048 | IL6/CAV1 | 2 |
| BP | GO:0070542 | | response to fatty acid | 2/43 | 86/18670 | 0.016757 | 0.045285 | 0.02048 | PPARG/CCNB1 | 2 |
| BP | GO:0002690 | | positive regulation of leukocyte chemotaxis | 2/43 | 87/18670 | 0.017127 | 0.046095 | 0.020847 | VEGFA/IL6 | 2 |
| BP | GO:0045844 | | positive regulation of striated muscle tissue development | 2/43 | 87/18670 | 0.017127 | 0.046095 | 0.020847 | BCL2/CCNB1 | 2 |
| BP | GO:0048477 | | oogenesis | 2/43 | 87/18670 | 0.017127 | 0.046095 | 0.020847 | BCL2/CCNB1 | 2 |
| BP | GO:0048636 | | positive regulation of muscle organ development | 2/43 | 87/18670 | 0.017127 | 0.046095 | 0.020847 | BCL2/CCNB1 | 2 |
| BP | GO:0030258 | | lipid modification | 3/43 | 238/18670 | 0.017354 | 0.046611 | 0.021081 | PPARG/CYP3A4/CYP1A1 | 3 |
| BP | GO:0051607 | | defense response to virus | 3/43 | 238/18670 | 0.017354 | 0.046611 | 0.021081 | RELA/BCL2/IL6 | 3 |
| BP | GO:0009791 | | post-embryonic development | 2/43 | 88/18670 | 0.0175 | 0.046766 | 0.021151 | VEGFA/BCL2 | 2 |
| BP | GO:1901863 | | positive regulation of muscle tissue development | 2/43 | 88/18670 | 0.0175 | 0.046766 | 0.021151 | BCL2/CCNB1 | 2 |
| BP | GO:1901888 | | regulation of cell junction assembly | 2/43 | 88/18670 | 0.0175 | 0.046766 | 0.021151 | VEGFA/CAV1 | 2 |
| BP | GO:1903321 | | negative regulation of protein modification by small protein conjugation or removal | 2/43 | 88/18670 | 0.0175 | 0.046766 | 0.021151 | RELA/CAV1 | 2 |
| BP | GO:2000177 | | regulation of neural precursor cell proliferation | 2/43 | 88/18670 | 0.0175 | 0.046766 | 0.021151 | VEGFA/HIF1A | 2 |
| BP | GO:0031348 | | negative regulation of defense response | 3/43 | 239/18670 | 0.017547 | 0.046844 | 0.021186 | PPARG/RB1/GSTP1 | 3 |
| BP | GO:0055013 | | cardiac muscle cell development | 2/43 | 89/18670 | 0.017876 | 0.047628 | 0.021541 | VEGFA/CCNB1 | 2 |
| BP | GO:0097306 | | cellular response to alcohol | 2/43 | 89/18670 | 0.017876 | 0.047628 | 0.021541 | PPARG/AHR | 2 |
| BP | GO:0031058 | | positive regulation of histone modification | 2/43 | 90/18670 | 0.018256 | 0.048446 | 0.02191 | VEGFA/CCNB1 | 2 |
| BP | GO:0034333 | | adherens junction assembly | 2/43 | 90/18670 | 0.018256 | 0.048446 | 0.02191 | VEGFA/BCL2 | 2 |
| BP | GO:0042475 | | odontogenesis of dentin-containing tooth | 2/43 | 90/18670 | 0.018256 | 0.048446 | 0.02191 | TP63/RUNX2 | 2 |
| BP | GO:0051304 | | chromosome separation | 2/43 | 90/18670 | 0.018256 | 0.048446 | 0.02191 | RB1/CCNB1 | 2 |
| BP | GO:0009266 | | response to temperature stimulus | 3/43 | 243/18670 | 0.01833 | 0.048593 | 0.021977 | PPARG/FOS/CASP8 | 3 |
| CC | GO:0005667 | | transcription factor complex | 8/43 | 365/19717 | 1.05E-06 | 0.000148 | 9.52E-05 | PPARG/RELA/FOS/RB1/HIF1A/PARP1/AHR/RUNX2 | 8 |
| CC | GO:0000307 | | cyclin-dependent protein kinase holoenzyme complex | 3/43 | 42/19717 | 0.000105 | 0.005851 | 0.003756 | CCND1/RB1/CCNB1 | 3 |
| CC | GO:0000790 | | nuclear chromatin | 6/43 | 377/19717 | 0.000157 | 0.005851 | 0.003756 | RELA/RB1/TP63/HIF1A/MYC/RUNX2 | 6 |
| CC | GO:0043209 | | myelin sheath | 3/43 | 49/19717 | 0.000166 | 0.005851 | 0.003756 | AKR1B1/BCL2/ERBB2 | 3 |
| CC | GO:0090575 | | RNA polymerase II transcription factor complex | 4/43 | 163/19717 | 0.000432 | 0.012056 | 0.007741 | PPARG/FOS/RB1/HIF1A | 4 |
| CC | GO:0045121 | | membrane raft | 5/43 | 315/19717 | 0.00059 | 0.012056 | 0.007741 | EGFR/CASP3/CASP8/CAV1/ICAM1 | 5 |
| CC | GO:0098857 | | membrane microdomain | 5/43 | 316/19717 | 0.000599 | 0.012056 | 0.007741 | EGFR/CASP3/CASP8/CAV1/ICAM1 | 5 |
| CC | GO:0098589 | | membrane region | 5/43 | 328/19717 | 0.000708 | 0.012148 | 0.0078 | EGFR/CASP3/CASP8/CAV1/ICAM1 | 5 |
| CC | GO:0005819 | | spindle | 5/43 | 347/19717 | 0.000912 | 0.012148 | 0.0078 | RB1/BIRC5/HSPB1/CCNB1/RASSF1 | 5 |
| CC | GO:1902554 | | serine/threonine protein kinase complex | 3/43 | 88/19717 | 0.000932 | 0.012148 | 0.0078 | CCND1/RB1/CCNB1 | 3 |
| CC | GO:0044798 | | nuclear transcription factor complex | 4/43 | 201/19717 | 0.000948 | 0.012148 | 0.0078 | PPARG/FOS/RB1/HIF1A | 4 |
| CC | GO:1902911 | | protein kinase complex | 3/43 | 109/19717 | 0.001727 | 0.018308 | 0.011754 | CCND1/RB1/CCNB1 | 3 |
| CC | GO:0005925 | | focal adhesion | 5/43 | 405/19717 | 0.001807 | 0.018308 | 0.011754 | EGFR/PLAU/CAV1/ICAM1/HSPB1 | 5 |
| CC | GO:0005924 | | cell-substrate adherens junction | 5/43 | 408/19717 | 0.001866 | 0.018308 | 0.011754 | EGFR/PLAU/CAV1/ICAM1/HSPB1 | 5 |
| CC | GO:0030055 | | cell-substrate junction | 5/43 | 412/19717 | 0.001948 | 0.018308 | 0.011754 | EGFR/PLAU/CAV1/ICAM1/HSPB1 | 5 |
| CC | GO:0009925 | | basal plasma membrane | 2/43 | 34/19717 | 0.002493 | 0.021973 | 0.014107 | EGFR/ERBB2 | 2 |
| CC | GO:0045178 | | basal part of cell | 2/43 | 51/19717 | 0.005535 | 0.04591 | 0.029476 | EGFR/ERBB2 | 2 |
| CC | GO:0005741 | | mitochondrial outer membrane | 3/43 | 178/19717 | 0.00685 | 0.048744 | 0.031295 | BCL2/CASP8/RAF1 | 3 |
| CC | GO:1904115 | | axon cytoplasm | 2/43 | 57/19717 | 0.006872 | 0.048744 | 0.031295 | HIF1A/HSPB1 | 2 |
| CC | GO:0098687 | | chromosomal region | 4/43 | 349/19717 | 0.006914 | 0.048744 | 0.031295 | BIRC5/CCNB1/PARP1/CHEK2 | 4 |
| MF | GO:0033613 | | activating transcription factor binding | 6/43 | 85/17697 | 5.43E-08 | 1.25E-05 | 6.34E-06 | PPARG/RELA/FOS/RB1/MYC/NFE2L2 | 6 |
| MF | GO:0044389 | | ubiquitin-like protein ligase binding | 8/43 | 308/17697 | 6.56E-07 | 7.58E-05 | 3.83E-05 | RELA/EGFR/BCL2/RB1/CASP8/HIF1A/CCNB1/CHEK2 | 8 |
| MF | GO:0031625 | | ubiquitin protein ligase binding | 7/43 | 290/17697 | 5.74E-06 | 0.000296 | 0.00015 | RELA/EGFR/BCL2/RB1/CASP8/HIF1A/CHEK2 | 7 |
| MF | GO:0097153 | | cysteine-type endopeptidase activity involved in apoptotic process | 3/43 | 15/17697 | 5.96E-06 | 0.000296 | 0.00015 | CASP9/CASP3/CASP8 | 3 |
| MF | GO:0070888 | | E-box binding | 4/43 | 50/17697 | 6.41E-06 | 0.000296 | 0.00015 | PPARG/HIF1A/MYC/AHR | 4 |
| MF | GO:0001228 | | DNA-binding transcription activator activity, RNA polymerase II-specific | 8/43 | 439/17697 | 9.10E-06 | 0.000351 | 0.000177 | RELA/FOS/TP63/HIF1A/MYC/NFE2L2/PARP1/RUNX2 | 8 |
| MF | GO:0005178 | | integrin binding | 5/43 | 132/17697 | 1.64E-05 | 0.000541 | 0.000274 | EGFR/PRKCA/ICAM1/VCAM1/COL3A1 | 5 |
| MF | GO:0070491 | | repressing transcription factor binding | 4/43 | 71/17697 | 2.61E-05 | 0.000753 | 0.000381 | PPARG/RELA/MYC/RUNX2 | 4 |
| MF | GO:0001085 | | RNA polymerase II transcription factor binding | 5/43 | 155/17697 | 3.56E-05 | 0.000913 | 0.000462 | PPARG/FOS/RB1/NFE2L2/AHR | 5 |
| MF | GO:0016712 | | oxidoreductase activity, acting on paired donors, with incorporation or reduction of molecular oxygen, reduced flavin or flavoprotein as one donor, and incorporation of one atom of oxygen | 3/43 | 32/17697 | 6.31E-05 | 0.001458 | 0.000737 | CYP3A4/CYP1A1/CYP1B1 | 3 |
| MF | GO:0019825 | | oxygen binding | 3/43 | 36/17697 | 9.02E-05 | 0.001895 | 0.000958 | CYP3A4/CYP1A1/CYP1B1 | 3 |
| MF | GO:0008395 | | steroid hydroxylase activity | 3/43 | 38/17697 | 0.000106 | 0.002045 | 0.001034 | CYP3A4/CYP1A1/CYP1B1 | 3 |
| MF | GO:0051879 | | Hsp90 protein binding | 3/43 | 41/17697 | 0.000134 | 0.002225 | 0.001125 | HIF1A/CYP1A1/AHR | 3 |
| MF | GO:0019207 | | kinase regulator activity | 5/43 | 207/17697 | 0.00014 | 0.002225 | 0.001125 | CCND1/CASP3/HSPB1/CCNB1/GSTP1 | 5 |
| MF | GO:0042826 | | histone deacetylase binding | 4/43 | 111/17697 | 0.00015 | 0.002225 | 0.001125 | RELA/CCND1/HIF1A/PARP1 | 4 |
| MF | GO:0001221 | | transcription cofactor binding | 3/43 | 43/17697 | 0.000154 | 0.002225 | 0.001125 | RELA/NFE2L2/AHR | 3 |
| MF | GO:0016538 | | cyclin-dependent protein serine/threonine kinase regulator activity | 3/43 | 49/17697 | 0.000228 | 0.003094 | 0.001565 | CCND1/CASP3/CCNB1 | 3 |
| MF | GO:0004955 | | prostaglandin receptor activity | 2/43 | 10/17697 | 0.000256 | 0.003289 | 0.001664 | PPARG/PTGER3 | 2 |
| MF | GO:0001102 | | RNA polymerase II activating transcription factor binding | 3/43 | 53/17697 | 0.000288 | 0.003332 | 0.001685 | FOS/RB1/NFE2L2 | 3 |
| MF | GO:0004954 | | prostanoid receptor activity | 2/43 | 11/17697 | 0.000313 | 0.003332 | 0.001685 | PPARG/PTGER3 | 2 |
| MF | GO:0043295 | | glutathione binding | 2/43 | 11/17697 | 0.000313 | 0.003332 | 0.001685 | GSTP1/GSTM1 | 2 |
| MF | GO:0020037 | | heme binding | 4/43 | 135/17697 | 0.000317 | 0.003332 | 0.001685 | PTGS1/CYP3A4/CYP1A1/CYP1B1 | 4 |
| MF | GO:0019838 | | growth factor binding | 4/43 | 137/17697 | 0.000336 | 0.003371 | 0.001705 | EGFR/ERBB2/COL3A1/IGFBP3 | 4 |
| MF | GO:0019903 | | protein phosphatase binding | 4/43 | 140/17697 | 0.000364 | 0.003463 | 0.001752 | PPARG/EGFR/BCL2/ERBB2 | 4 |
| MF | GO:1900750 | | oligopeptide binding | 2/43 | 12/17697 | 0.000375 | 0.003463 | 0.001752 | GSTP1/GSTM1 | 2 |
| MF | GO:0046906 | | tetrapyrrole binding | 4/43 | 145/17697 | 0.000416 | 0.003699 | 0.001871 | PTGS1/CYP3A4/CYP1A1/CYP1B1 | 4 |
| MF | GO:0035257 | | nuclear hormone receptor binding | 4/43 | 152/17697 | 0.000497 | 0.004256 | 0.002153 | PPARG/RB1/HIF1A/PARP1 | 4 |
| MF | GO:0016705 | | oxidoreductase activity, acting on paired donors, with incorporation or reduction of molecular oxygen | 4/43 | 159/17697 | 0.000589 | 0.004727 | 0.002391 | PTGS1/CYP3A4/CYP1A1/CYP1B1 | 4 |
| MF | GO:0004953 | | icosanoid receptor activity | 2/43 | 15/17697 | 0.000593 | 0.004727 | 0.002391 | PPARG/PTGER3 | 2 |
| MF | GO:0005123 | | death receptor binding | 2/43 | 17/17697 | 0.000766 | 0.00571 | 0.002888 | CASP3/CASP8 | 2 |
| MF | GO:0035173 | | histone kinase activity | 2/43 | 17/17697 | 0.000766 | 0.00571 | 0.002888 | PRKCA/CCNB1 | 2 |
| MF | GO:0019887 | | protein kinase regulator activity | 4/43 | 180/17697 | 0.000937 | 0.006746 | 0.003412 | CCND1/CASP3/HSPB1/CCNB1 | 4 |
| MF | GO:0046332 | | SMAD binding | 3/43 | 80/17697 | 0.000964 | 0.006746 | 0.003412 | FOS/PARP1/COL3A1 | 3 |
| MF | GO:0019902 | | phosphatase binding | 4/43 | 185/17697 | 0.001038 | 0.006848 | 0.003464 | PPARG/EGFR/BCL2/ERBB2 | 4 |
| MF | GO:0051427 | | hormone receptor binding | 4/43 | 185/17697 | 0.001038 | 0.006848 | 0.003464 | PPARG/RB1/HIF1A/PARP1 | 4 |
| MF | GO:0016209 | | antioxidant activity | 3/43 | 86/17697 | 0.001188 | 0.007625 | 0.003857 | PTGS1/GSTP1/NQO1 | 3 |
| MF | GO:0001223 | | transcription coactivator binding | 2/43 | 23/17697 | 0.001412 | 0.00855 | 0.004325 | RELA/AHR | 2 |
| MF | GO:0070412 | | R-SMAD binding | 2/43 | 23/17697 | 0.001412 | 0.00855 | 0.004325 | FOS/PARP1 | 2 |
| MF | GO:0035258 | | steroid hormone receptor binding | 3/43 | 92/17697 | 0.001443 | 0.00855 | 0.004325 | PPARG/RB1/PARP1 | 3 |
| MF | GO:0004497 | | monooxygenase activity | 3/43 | 99/17697 | 0.001782 | 0.010176 | 0.005147 | CYP3A4/CYP1A1/CYP1B1 | 3 |
| MF | GO:0004709 | | MAP kinase kinase kinase activity | 2/43 | 26/17697 | 0.001806 | 0.010176 | 0.005147 | EGFR/RAF1 | 2 |
| MF | GO:0001968 | | fibronectin binding | 2/43 | 27/17697 | 0.001948 | 0.010463 | 0.005292 | VEGFA/IGFBP3 | 2 |
| MF | GO:0004364 | | glutathione transferase activity | 2/43 | 27/17697 | 0.001948 | 0.010463 | 0.005292 | GSTP1/GSTM1 | 2 |
| MF | GO:0030291 | | protein serine/threonine kinase inhibitor activity | 2/43 | 32/17697 | 0.002731 | 0.014338 | 0.007252 | CASP3/HSPB1 | 2 |
| MF | GO:0004197 | | cysteine-type endopeptidase activity | 3/43 | 116/17697 | 0.002798 | 0.014363 | 0.007265 | CASP9/CASP3/CASP8 | 3 |
| MF | GO:0031072 | | heat shock protein binding | 3/43 | 119/17697 | 0.003007 | 0.015103 | 0.007639 | HIF1A/CYP1A1/AHR | 3 |
| MF | GO:0002020 | | protease binding | 3/43 | 128/17697 | 0.003693 | 0.018151 | 0.009181 | BCL2/CASP3/COL3A1 | 3 |
| MF | GO:0016248 | | channel inhibitor activity | 2/43 | 38/17697 | 0.003835 | 0.018457 | 0.009336 | BCL2/CAV1 | 2 |
| MF | GO:0035326 | | enhancer binding | 3/43 | 133/17697 | 0.004112 | 0.019385 | 0.009805 | RELA/NFE2L2/AHR | 3 |
| MF | GO:0030331 | | estrogen receptor binding | 2/43 | 42/17697 | 0.004668 | 0.021568 | 0.010909 | PPARG/PARP1 | 2 |
| MF | GO:0005126 | | cytokine receptor binding | 4/43 | 286/17697 | 0.005023 | 0.022752 | 0.011508 | VEGFA/IL6/CASP3/CASP8 | 4 |
| MF | GO:0032813 | | tumor necrosis factor receptor superfamily binding | 2/43 | 46/17697 | 0.005578 | 0.023959 | 0.012119 | CASP3/CASP8 | 2 |
| MF | GO:0042805 | | actinin binding | 2/43 | 46/17697 | 0.005578 | 0.023959 | 0.012119 | PPARG/RELA | 2 |
| MF | GO:0042277 | | peptide binding | 4/43 | 295/17697 | 0.005601 | 0.023959 | 0.012119 | PPARG/ACHE/GSTP1/GSTM1 | 4 |
| MF | GO:0004879 | | nuclear receptor activity | 2/43 | 47/17697 | 0.005816 | 0.023993 | 0.012136 | PPARG/AHR | 2 |
| MF | GO:0098531 | | transcription factor activity, direct ligand regulated sequence-specific DNA binding | 2/43 | 47/17697 | 0.005816 | 0.023993 | 0.012136 | PPARG/AHR | 2 |
| MF | GO:0005506 | | iron ion binding | 3/43 | 152/17697 | 0.005962 | 0.024163 | 0.012222 | CYP3A4/CYP1A1/CYP1B1 | 3 |
| MF | GO:0050839 | | cell adhesion molecule binding | 5/43 | 499/17697 | 0.006955 | 0.027702 | 0.014012 | EGFR/PRKCA/ICAM1/VCAM1/COL3A1 | 5 |
| MF | GO:0004601 | | peroxidase activity | 2/43 | 52/17697 | 0.00708 | 0.027721 | 0.014021 | PTGS1/GSTP1 | 2 |
| MF | GO:0001047 | | core promoter binding | 2/43 | 55/17697 | 0.007893 | 0.030387 | 0.01537 | FOS/MYC | 2 |
| MF | GO:0016684 | | oxidoreductase activity, acting on peroxide as acceptor | 2/43 | 56/17697 | 0.008173 | 0.030449 | 0.015402 | PTGS1/GSTP1 | 2 |
| MF | GO:0043621 | | protein self-association | 2/43 | 56/17697 | 0.008173 | 0.030449 | 0.015402 | PPARG/ACHE | 2 |
| MF | GO:0050840 | | extracellular matrix binding | 2/43 | 57/17697 | 0.008457 | 0.031008 | 0.015684 | ACHE/VEGFA | 2 |
| MF | GO:0016765 | | transferase activity, transferring alkyl or aryl (other than methyl) groups | 2/43 | 58/17697 | 0.008745 | 0.031565 | 0.015966 | GSTP1/GSTM1 | 2 |
| MF | GO:0004714 | | transmembrane receptor protein tyrosine kinase activity | 2/43 | 62/17697 | 0.009943 | 0.035205 | 0.017807 | EGFR/ERBB2 | 2 |
| MF | GO:0008234 | | cysteine-type peptidase activity | 3/43 | 184/17697 | 0.010059 | 0.035205 | 0.017807 | CASP9/CASP3/CASP8 | 3 |
| MF | GO:0004860 | | protein kinase inhibitor activity | 2/43 | 63/17697 | 0.010254 | 0.035352 | 0.017882 | CASP3/HSPB1 | 2 |
| MF | GO:0033218 | | amide binding | 4/43 | 356/17697 | 0.010716 | 0.036403 | 0.018413 | PPARG/ACHE/GSTP1/GSTM1 | 4 |
| MF | GO:0002039 | | p53 binding | 2/43 | 66/17697 | 0.01121 | 0.037529 | 0.018983 | TP63/HIF1A | 2 |
| MF | GO:0019210 | | kinase inhibitor activity | 2/43 | 67/17697 | 0.011537 | 0.038073 | 0.019258 | CASP3/HSPB1 | 2 |
| MF | GO:0001618 | | virus receptor activity | 2/43 | 74/17697 | 0.013944 | 0.044737 | 0.022629 | EGFR/ICAM1 | 2 |
| MF | GO:0104005 | | hijacked molecular function | 2/43 | 74/17697 | 0.013944 | 0.044737 | 0.022629 | EGFR/ICAM1 | 2 |
| MF | GO:0019199 | | transmembrane receptor protein kinase activity | 2/43 | 79/17697 | 0.015785 | 0.049949 | 0.025265 | EGFR/ERBB2 | 2 |

Supplemental table 5. KEGG analysis of 43 common target genes of quercetin, cisplatin, and cervical cancer.

| ID | Description | GeneRatio | BgRatio | pvalue | p.adjust | qvalue | geneID | Count |
| --- | --- | --- | --- | --- | --- | --- | --- | --- |
| hsa05167 | Kaposi sarcoma-associated herpesvirus infection | 13/43 | 193/8105 | 1.03E-11 | 1.83E-09 | 8.00E-10 | RELA/VEGFA/CCND1/FOS/CASP9/RB1/IL6/CASP3/CASP8/RAF1/HIF1A/MYC/ICAM1 | 13 |
| hsa05161 | Hepatitis B | 12/43 | 162/8105 | 2.43E-11 | 1.83E-09 | 8.00E-10 | RELA/BCL2/FOS/CASP9/RB1/IL6/CASP3/CASP8/RAF1/PRKCA/MYC/BIRC5 | 12 |
| hsa05219 | Bladder cancer | 8/43 | 41/8105 | 2.65E-11 | 1.83E-09 | 8.00E-10 | EGFR/VEGFA/CCND1/RB1/RAF1/ERBB2/MYC/RASSF1 | 8 |
| hsa05417 | Lipid and atherosclerosis | 13/43 | 215/8105 | 4.07E-11 | 2.10E-09 | 9.20E-10 | PPARG/RELA/BCL2/FOS/CASP9/IL6/CASP3/CASP8/PRKCA/CYP1A1/ICAM1/VCAM1/NFE2L2 | 13 |
| hsa05215 | Prostate cancer | 10/43 | 97/8105 | 5.20E-11 | 2.13E-09 | 9.32E-10 | RELA/EGFR/CCND1/BCL2/CASP9/PLAU/RB1/RAF1/ERBB2/GSTP1 | 10 |
| hsa04933 | AGE-RAGE signaling pathway in diabetic complications | 10/43 | 100/8105 | 7.08E-11 | 2.13E-09 | 9.32E-10 | RELA/VEGFA/CCND1/BCL2/IL6/CASP3/PRKCA/ICAM1/VCAM1/COL3A1 | 10 |
| hsa05163 | Human cytomegalovirus infection | 13/43 | 225/8105 | 7.21E-11 | 2.13E-09 | 9.32E-10 | RELA/EGFR/VEGFA/CCND1/CASP9/RB1/IL6/CASP3/CASP8/RAF1/PRKCA/MYC/PTGER3 | 13 |
| hsa05418 | Fluid shear stress and atherosclerosis | 11/43 | 139/8105 | 9.13E-11 | 2.36E-09 | 1.03E-09 | RELA/VEGFA/BCL2/FOS/CAV1/ICAM1/VCAM1/GSTP1/NFE2L2/NQO1/GSTM1 | 11 |
| hsa05210 | Colorectal cancer | 9/43 | 86/8105 | 4.67E-10 | 1.08E-08 | 4.70E-09 | EGFR/CCND1/BCL2/FOS/CASP9/CASP3/RAF1/MYC/BIRC5 | 9 |
| hsa05223 | Non-small cell lung cancer | 8/43 | 72/8105 | 2.95E-09 | 5.69E-08 | 2.49E-08 | EGFR/CCND1/CASP9/RB1/RAF1/PRKCA/ERBB2/RASSF1 | 8 |
| hsa01524 | Platinum drug resistance | 8/43 | 73/8105 | 3.30E-09 | 5.69E-08 | 2.49E-08 | BCL2/CASP9/CASP3/CASP8/ERBB2/BIRC5/GSTP1/GSTM1 | 8 |
| hsa04115 | p53 signaling pathway | 8/43 | 73/8105 | 3.30E-09 | 5.69E-08 | 2.49E-08 | CCND1/BCL2/CASP9/CASP3/CASP8/CCNB1/CHEK2/IGFBP3 | 8 |
| hsa05206 | MicroRNAs in cancer | 13/43 | 310/8105 | 3.80E-09 | 6.04E-08 | 2.64E-08 | EGFR/VEGFA/CCND1/BCL2/PLAU/CASP3/TP63/RAF1/PRKCA/ERBB2/MYC/CYP1B1/RASSF1 | 13 |
| hsa05212 | Pancreatic cancer | 8/43 | 76/8105 | 4.57E-09 | 6.76E-08 | 2.96E-08 | RELA/EGFR/VEGFA/CCND1/CASP9/RB1/RAF1/ERBB2 | 8 |
| hsa05205 | Proteoglycans in cancer | 11/43 | 205/8105 | 5.87E-09 | 8.10E-08 | 3.54E-08 | EGFR/VEGFA/CCND1/PLAU/CASP3/RAF1/PRKCA/HIF1A/ERBB2/CAV1/MYC | 11 |
| hsa05225 | Hepatocellular carcinoma | 10/43 | 168/8105 | 1.19E-08 | 1.54E-07 | 6.73E-08 | EGFR/CCND1/RB1/RAF1/PRKCA/MYC/GSTP1/NFE2L2/NQO1/GSTM1 | 10 |
| hsa04210 | Apoptosis | 9/43 | 136/8105 | 2.81E-08 | 3.42E-07 | 1.50E-07 | RELA/BCL2/FOS/CASP9/CASP3/CASP8/RAF1/BIRC5/PARP1 | 9 |
| hsa05169 | Epstein-Barr virus infection | 10/43 | 202/8105 | 6.93E-08 | 7.97E-07 | 3.49E-07 | RELA/CCND1/BCL2/CASP9/RB1/IL6/CASP3/CASP8/MYC/ICAM1 | 10 |
| hsa04066 | HIF-1 signaling pathway | 8/43 | 109/8105 | 8.10E-08 | 8.83E-07 | 3.86E-07 | RELA/EGFR/VEGFA/BCL2/IL6/PRKCA/HIF1A/ERBB2 | 8 |
| hsa04218 | Cellular senescence | 9/43 | 156/8105 | 9.28E-08 | 9.60E-07 | 4.20E-07 | RELA/CCND1/RB1/IL6/RAF1/MYC/CCNB1/CHEK2/IGFBP3 | 9 |
| hsa05160 | Hepatitis C | 9/43 | 157/8105 | 9.80E-08 | 9.66E-07 | 4.23E-07 | RELA/EGFR/CCND1/CASP9/RB1/CASP3/CASP8/RAF1/MYC | 9 |
| hsa01521 | EGFR tyrosine kinase inhibitor resistance | 7/43 | 79/8105 | 1.55E-07 | 1.46E-06 | 6.38E-07 | EGFR/VEGFA/BCL2/IL6/RAF1/PRKCA/ERBB2 | 7 |
| hsa05222 | Small cell lung cancer | 7/43 | 92/8105 | 4.45E-07 | 4.01E-06 | 1.75E-06 | RELA/CCND1/BCL2/CASP9/RB1/CASP3/MYC | 7 |
| hsa05213 | Endometrial cancer | 6/43 | 58/8105 | 5.12E-07 | 4.42E-06 | 1.93E-06 | EGFR/CCND1/CASP9/RAF1/ERBB2/MYC | 6 |
| hsa05162 | Measles | 8/43 | 139/8105 | 5.35E-07 | 4.43E-06 | 1.94E-06 | RELA/CCND1/BCL2/FOS/CASP9/IL6/CASP3/CASP8 | 8 |
| hsa04215 | Apoptosis - multiple species | 5/43 | 32/8105 | 5.99E-07 | 4.77E-06 | 2.09E-06 | BCL2/CASP9/CASP3/CASP8/BIRC5 | 5 |
| hsa05416 | Viral myocarditis | 6/43 | 60/8105 | 6.28E-07 | 4.82E-06 | 2.11E-06 | CCND1/CASP9/CASP3/CASP8/CAV1/ICAM1 | 6 |
| hsa01522 | Endocrine resistance | 7/43 | 98/8105 | 6.87E-07 | 5.08E-06 | 2.22E-06 | EGFR/CCND1/BCL2/FOS/RB1/RAF1/ERBB2 | 7 |
| hsa05170 | Human immunodeficiency virus 1 infection | 9/43 | 212/8105 | 1.26E-06 | 8.99E-06 | 3.93E-06 | RELA/BCL2/FOS/CASP9/CASP3/CASP8/RAF1/PRKCA/CCNB1 | 9 |
| hsa04151 | PI3K-Akt signaling pathway | 11/43 | 354/8105 | 1.54E-06 | 1.06E-05 | 4.66E-06 | RELA/EGFR/VEGFA/CCND1/BCL2/CASP9/IL6/RAF1/PRKCA/ERBB2/MYC | 11 |
| hsa04668 | TNF signaling pathway | 7/43 | 112/8105 | 1.70E-06 | 1.14E-05 | 4.97E-06 | RELA/FOS/IL6/CASP3/CASP8/ICAM1/VCAM1 | 7 |
| hsa04010 | MAPK signaling pathway | 10/43 | 294/8105 | 2.24E-06 | 1.45E-05 | 6.34E-06 | RELA/EGFR/VEGFA/FOS/CASP3/RAF1/PRKCA/ERBB2/MYC/HSPB1 | 10 |
| hsa05164 | Influenza A | 8/43 | 172/8105 | 2.69E-06 | 1.69E-05 | 7.38E-06 | RELA/CASP9/IL6/CASP3/CASP8/RAF1/PRKCA/ICAM1 | 8 |
| hsa04926 | Relaxin signaling pathway | 7/43 | 129/8105 | 4.40E-06 | 2.68E-05 | 1.17E-05 | RELA/EGFR/VEGFA/FOS/RAF1/PRKCA/COL3A1 | 7 |
| hsa04510 | Focal adhesion | 8/43 | 201/8105 | 8.56E-06 | 5.06E-05 | 2.21E-05 | EGFR/VEGFA/CCND1/BCL2/RAF1/PRKCA/ERBB2/CAV1 | 8 |
| hsa05224 | Breast cancer | 7/43 | 147/8105 | 1.04E-05 | 6.00E-05 | 2.62E-05 | EGFR/CCND1/FOS/RB1/RAF1/ERBB2/MYC | 7 |
| hsa05134 | Legionellosis | 5/43 | 57/8105 | 1.13E-05 | 6.21E-05 | 2.72E-05 | RELA/CASP9/IL6/CASP3/CASP8 | 5 |
| hsa05226 | Gastric cancer | 7/43 | 149/8105 | 1.14E-05 | 6.21E-05 | 2.72E-05 | EGFR/CCND1/BCL2/RB1/RAF1/ERBB2/MYC | 7 |
| hsa04370 | VEGF signaling pathway | 5/43 | 59/8105 | 1.34E-05 | 7.11E-05 | 3.11E-05 | VEGFA/CASP9/RAF1/PRKCA/HSPB1 | 5 |
| hsa05146 | Amoebiasis | 6/43 | 102/8105 | 1.43E-05 | 7.41E-05 | 3.24E-05 | RELA/IL6/CASP3/PRKCA/HSPB1/COL3A1 | 6 |
| hsa04064 | NF-kappa B signaling pathway | 6/43 | 104/8105 | 1.60E-05 | 7.91E-05 | 3.46E-05 | RELA/BCL2/PLAU/ICAM1/VCAM1/PARP1 | 6 |
| hsa05166 | Human T-cell leukemia virus 1 infection | 8/43 | 219/8105 | 1.60E-05 | 7.91E-05 | 3.46E-05 | RELA/CCND1/FOS/RB1/IL6/MYC/ICAM1/CHEK2 | 8 |
| hsa04928 | Parathyroid hormone synthesis, secretion and action | 6/43 | 106/8105 | 1.79E-05 | 8.60E-05 | 3.76E-05 | EGFR/BCL2/FOS/RAF1/PRKCA/RUNX2 | 6 |
| hsa05230 | Central carbon metabolism in cancer | 5/43 | 70/8105 | 3.10E-05 | 0.000146 | 6.38E-05 | EGFR/RAF1/HIF1A/ERBB2/MYC | 5 |
| hsa04919 | Thyroid hormone signaling pathway | 6/43 | 121/8105 | 3.79E-05 | 0.000175 | 7.63E-05 | CCND1/CASP9/RAF1/PRKCA/HIF1A/MYC | 6 |
| hsa05152 | Tuberculosis | 7/43 | 180/8105 | 3.89E-05 | 0.000175 | 7.64E-05 | RELA/BCL2/CASP9/IL6/CASP3/CASP8/RAF1 | 7 |
| hsa05143 | African trypanosomiasis | 4/43 | 37/8105 | 3.99E-05 | 0.000175 | 7.64E-05 | IL6/PRKCA/ICAM1/VCAM1 | 4 |
| hsa05132 | Salmonella infection | 8/43 | 249/8105 | 4.05E-05 | 0.000175 | 7.64E-05 | RELA/BCL2/FOS/IL6/CASP3/CASP8/RAF1/MYC | 8 |
| hsa05214 | Glioma | 5/43 | 75/8105 | 4.34E-05 | 0.000183 | 8.02E-05 | EGFR/CCND1/RB1/RAF1/PRKCA | 5 |
| hsa05220 | Chronic myeloid leukemia | 5/43 | 76/8105 | 4.63E-05 | 0.000192 | 8.38E-05 | RELA/CCND1/RB1/RAF1/MYC | 5 |
| hsa00980 | Metabolism of xenobiotics by cytochrome P450 | 5/43 | 78/8105 | 5.25E-05 | 0.000213 | 9.31E-05 | CYP3A4/CYP1A1/CYP1B1/GSTP1/GSTM1 | 5 |
| hsa05202 | Transcriptional misregulation in cancer | 7/43 | 192/8105 | 5.87E-05 | 0.000234 | 0.000102 | PPARG/RELA/PLAU/IL6/MYC/RUNX2/IGFBP3 | 7 |
| hsa05204 | Chemical carcinogenesis | 5/43 | 83/8105 | 7.07E-05 | 0.000276 | 0.000121 | CYP3A4/CYP1A1/CYP1B1/GSTP1/GSTM1 | 5 |
| hsa04012 | ErbB signaling pathway | 5/43 | 85/8105 | 7.93E-05 | 0.000304 | 0.000133 | EGFR/RAF1/PRKCA/ERBB2/MYC | 5 |
| hsa05235 | PD-L1 expression and PD-1 checkpoint pathway in cancer | 5/43 | 89/8105 | 9.88E-05 | 0.000372 | 0.000163 | RELA/EGFR/FOS/RAF1/HIF1A | 5 |
| hsa04657 | IL-17 signaling pathway | 5/43 | 94/8105 | 0.000128 | 0.000474 | 0.000207 | RELA/FOS/IL6/CASP3/CASP8 | 5 |
| hsa05231 | Choline metabolism in cancer | 5/43 | 98/8105 | 0.000156 | 0.000567 | 0.000248 | EGFR/FOS/RAF1/PRKCA/HIF1A | 5 |
| hsa04630 | JAK-STAT signaling pathway | 6/43 | 162/8105 | 0.000192 | 0.000686 | 0.0003 | EGFR/CCND1/BCL2/IL6/RAF1/MYC | 6 |
| hsa04659 | Th17 cell differentiation | 5/43 | 107/8105 | 0.000236 | 0.000827 | 0.000362 | RELA/FOS/IL6/HIF1A/AHR | 5 |
| hsa05145 | Toxoplasmosis | 5/43 | 112/8105 | 0.000292 | 0.001 | 0.000437 | RELA/BCL2/CASP9/CASP3/CASP8 | 5 |
| hsa05165 | Human papillomavirus infection | 8/43 | 331/8105 | 0.000295 | 0.001 | 0.000437 | RELA/EGFR/VEGFA/CCND1/RB1/CASP3/CASP8/RAF1 | 8 |
| hsa05221 | Acute myeloid leukemia | 4/43 | 67/8105 | 0.000413 | 0.001379 | 0.000603 | RELA/CCND1/RAF1/MYC | 4 |
| hsa04110 | Cell cycle | 5/43 | 124/8105 | 0.000467 | 0.001535 | 0.000671 | CCND1/RB1/MYC/CCNB1/CHEK2 | 5 |
| hsa04917 | Prolactin signaling pathway | 4/43 | 70/8105 | 0.000488 | 0.00158 | 0.000691 | RELA/CCND1/FOS/RAF1 | 4 |
| hsa05218 | Melanoma | 4/43 | 72/8105 | 0.000544 | 0.001726 | 0.000755 | EGFR/CCND1/RB1/RAF1 | 4 |
| hsa05130 | Pathogenic Escherichia coli infection | 6/43 | 197/8105 | 0.00055 | 0.001726 | 0.000755 | RELA/FOS/CASP9/IL6/CASP3/CASP8 | 6 |
| hsa04068 | FoxO signaling pathway | 5/43 | 131/8105 | 0.000601 | 0.001857 | 0.000812 | EGFR/CCND1/IL6/RAF1/CCNB1 | 5 |
| hsa05133 | Pertussis | 4/43 | 76/8105 | 0.000668 | 0.002033 | 0.000889 | RELA/FOS/IL6/CASP3 | 4 |
| hsa05216 | Thyroid cancer | 3/43 | 37/8105 | 0.000953 | 0.00286 | 0.001251 | PPARG/CCND1/MYC | 3 |
| hsa04932 | Non-alcoholic fatty liver disease | 5/43 | 150/8105 | 0.001109 | 0.00328 | 0.001434 | RELA/FOS/IL6/CASP3/CASP8 | 5 |
| hsa04921 | Oxytocin signaling pathway | 5/43 | 154/8105 | 0.001248 | 0.003638 | 0.001591 | EGFR/CCND1/FOS/RAF1/PRKCA | 5 |
| hsa04014 | Ras signaling pathway | 6/43 | 232/8105 | 0.001294 | 0.003721 | 0.001627 | RELA/EGFR/VEGFA/RAF1/PRKCA/RASSF1 | 6 |
| hsa05323 | Rheumatoid arthritis | 4/43 | 93/8105 | 0.001424 | 0.004038 | 0.001766 | VEGFA/FOS/IL6/ICAM1 | 4 |
| hsa05142 | Chagas disease | 4/43 | 102/8105 | 0.002002 | 0.005601 | 0.002449 | RELA/FOS/IL6/CASP8 | 4 |
| hsa04620 | Toll-like receptor signaling pathway | 4/43 | 104/8105 | 0.00215 | 0.005855 | 0.002561 | RELA/FOS/IL6/CASP8 | 4 |
| hsa04625 | C-type lectin receptor signaling pathway | 4/43 | 104/8105 | 0.00215 | 0.005855 | 0.002561 | RELA/IL6/CASP8/RAF1 | 4 |
| hsa05144 | Malaria | 3/43 | 50/8105 | 0.002292 | 0.006161 | 0.002695 | IL6/ICAM1/VCAM1 | 3 |
| hsa04725 | Cholinergic synapse | 4/43 | 113/8105 | 0.002908 | 0.007718 | 0.003375 | ACHE/BCL2/FOS/PRKCA | 4 |
| hsa05022 | Pathways of neurodegeneration - multiple diseases | 8/43 | 475/8105 | 0.003088 | 0.008018 | 0.003507 | RELA/BCL2/CASP9/IL6/CASP3/CASP8/RAF1/PRKCA | 8 |
| hsa04726 | Serotonergic synapse | 4/43 | 115/8105 | 0.003099 | 0.008018 | 0.003507 | PTGS1/CASP3/RAF1/PRKCA | 4 |
| hsa04071 | Sphingolipid signaling pathway | 4/43 | 119/8105 | 0.003505 | 0.008849 | 0.00387 | RELA/BCL2/RAF1/PRKCA | 4 |
| hsa04935 | Growth hormone synthesis, secretion and action | 4/43 | 119/8105 | 0.003505 | 0.008849 | 0.00387 | FOS/RAF1/PRKCA/IGFBP3 | 4 |
| hsa00140 | Steroid hormone biosynthesis | 3/43 | 61/8105 | 0.004042 | 0.01008 | 0.004408 | CYP3A4/CYP1A1/CYP1B1 | 3 |
| hsa05203 | Viral carcinogenesis | 5/43 | 204/8105 | 0.004255 | 0.010485 | 0.004586 | RELA/CCND1/RB1/CASP3/CASP8 | 5 |
| hsa04650 | Natural killer cell mediated cytotoxicity | 4/43 | 131/8105 | 0.00494 | 0.012031 | 0.005261 | CASP3/RAF1/PRKCA/ICAM1 | 4 |
| hsa05211 | Renal cell carcinoma | 3/43 | 69/8105 | 0.005713 | 0.013752 | 0.006014 | VEGFA/RAF1/HIF1A | 3 |
| hsa04915 | Estrogen signaling pathway | 4/43 | 138/8105 | 0.005937 | 0.01399 | 0.006118 | EGFR/BCL2/FOS/RAF1 | 4 |
| hsa05120 | Epithelial cell signaling in Helicobacter pylori infection | 3/43 | 70/8105 | 0.005947 | 0.01399 | 0.006118 | RELA/EGFR/CASP3 | 3 |
| hsa00982 | Drug metabolism - cytochrome P450 | 3/43 | 72/8105 | 0.006432 | 0.014961 | 0.006543 | CYP3A4/GSTP1/GSTM1 | 3 |
| hsa05171 | Coronavirus disease - COVID-19 | 5/43 | 232/8105 | 0.007301 | 0.016792 | 0.007344 | RELA/EGFR/FOS/IL6/PRKCA | 5 |
| hsa04020 | Calcium signaling pathway | 5/43 | 240/8105 | 0.008396 | 0.019099 | 0.008353 | EGFR/VEGFA/PRKCA/ERBB2/PTGER3 | 5 |
| hsa00983 | Drug metabolism - other enzymes | 3/43 | 80/8105 | 0.008605 | 0.019361 | 0.008467 | CYP3A4/GSTP1/GSTM1 | 3 |
| hsa04934 | Cushing syndrome | 4/43 | 155/8105 | 0.008898 | 0.019805 | 0.008661 | EGFR/CCND1/RB1/AHR | 4 |
| hsa04662 | B cell receptor signaling pathway | 3/43 | 82/8105 | 0.009208 | 0.020264 | 0.008862 | RELA/FOS/RAF1 | 3 |
| hsa04390 | Hippo signaling pathway | 4/43 | 157/8105 | 0.0093 | 0.020264 | 0.008862 | CCND1/MYC/BIRC5/RASSF1 | 4 |
| hsa04540 | Gap junction | 3/43 | 88/8105 | 0.011161 | 0.024067 | 0.010525 | EGFR/RAF1/PRKCA | 3 |
| hsa01523 | Antifolate resistance | 2/43 | 31/8105 | 0.011597 | 0.024749 | 0.010823 | RELA/IL6 | 2 |
| hsa05010 | Alzheimer disease | 6/43 | 369/8105 | 0.01252 | 0.026445 | 0.011565 | RELA/CASP9/IL6/CASP3/CASP8/RAF1 | 6 |
| hsa04912 | GnRH signaling pathway | 3/43 | 93/8105 | 0.01296 | 0.027097 | 0.01185 | EGFR/RAF1/PRKCA | 3 |
| hsa04621 | NOD-like receptor signaling pathway | 4/43 | 181/8105 | 0.015069 | 0.031194 | 0.013642 | RELA/BCL2/IL6/CASP8 | 4 |
| hsa04660 | T cell receptor signaling pathway | 3/43 | 104/8105 | 0.01747 | 0.035806 | 0.015659 | RELA/FOS/RAF1 | 3 |
| hsa00380 | Tryptophan metabolism | 2/43 | 42/8105 | 0.020699 | 0.042007 | 0.018371 | CYP1A1/CYP1B1 | 2 |
| hsa05415 | Diabetic cardiomyopathy | 4/43 | 203/8105 | 0.02201 | 0.044233 | 0.019344 | RELA/PRKCA/PARP1/COL3A1 | 4 |
| hsa04670 | Leukocyte transendothelial migration | 3/43 | 114/8105 | 0.022244 | 0.044273 | 0.019362 | PRKCA/ICAM1/VCAM1 | 3 |
| hsa04015 | Rap1 signaling pathway | 4/43 | 210/8105 | 0.02457 | 0.048438 | 0.021183 | EGFR/VEGFA/RAF1/PRKCA | 4 |
| hsa04722 | Neurotrophin signaling pathway | 3/43 | 119/8105 | 0.024872 | 0.048571 | 0.021241 | RELA/BCL2/RAF1 | 3 |
